# Supplementary material for: Eczema Care Online: development and qualitative optimisation of an online behavioural intervention to support self-management in young people with eczema
Source: BMJ Open. 2022 Apr 19;12(4):e056867. doi: 10.1136/bmjopen-2021-056867 (PMC9021764; doi:10.1136/bmjopen-2021-056867)
Supplement: Supplementary data [file bmjopen-2021-056867supp001.pdf]

Supplementary Material 1: Behavioural analysis for the Eczema Care Online (ECO) intervention for young people using the Behaviour Change Wheel (BCW), Theoretical Domains Framework (TDF) and Behaviour Change Techniques Taxonomy (BCTv1)

Key: TCS = Topical Corticosteroids; YP = Young People; EO = Barrier emerged from expert opinion; QSR = Barrier/facilitator emerged from systematic review of qualitative literature with adults with eczema and parents/carers of children with eczema; QI = Barrier/facilitator emerged from qualitative interview research with young people.

**Barriers/*facilitator* to target behaviour**	**Intervention components**	**Target construct (BCW)**	**Theoretical domain (TDF)**	**Intervention function (BCW)**	**Behaviour Change Technique (using BCTv1)**
**Target behaviour: Increased emollient use**					
Belief that emollients (in general or a specific brand) <u>do little</u> to control eczema [QSR; QI]<br><br>*Belief that emollients are useful for preventing dry/cracking skin [QI]*	• Provide persuasive and credible information about the effectiveness of emollients, including scientific evidence, user quotes, and videos<br>• Provide rationale for how emollients control eczema	Psychological capability; Reflective motivation; Social opportunity	Beliefs about consequences; Knowledge; Social Influences	Education; Persuasion; Modelling	5.1 Information about health consequences<br>6.2 Social comparison<br>6.3 Information about others' approval<br>9.1 Credible source
	The 2-week challenge:<br>• Provide an emollient chart to allow YP to record how their skin is after applying emollients	Reflective Motivation	Beliefs about consequences	Education; Persuasion	5.1 Information about health consequences<br>2.4 Self-monitoring of outcomes of behaviour
	• Provide advice on how to choose an effective emollient (e.g. list of available emollients, disadvantages of using cosmetic moisturisers)	Psychological capability	Skills	Training	4.1 Instructions on how to perform the behaviour
Concerns about the safety and side effects (e.g. stinging, dependency) of emollients smell, feel, and appearance of emollients [QSR; QI QI]	• Provide persuasive and credible information about the safety of emollients and risk of side effects (including their flammability), including scientific evidence, user quotes and videos	Psychological capability; Reflective motivation; Social opportunity	Beliefs about consequences; Knowledge; Social Influences	Education; Persuasion; Modelling	5.1 Information about health consequences<br>6.2 Social comparison<br>6.3 Information about others' approval<br>9.1 Credible source
	• Provide advice on how to choose the right emollient (e.g. using different emollients at different times of the day, highlighting that different	Psychological capability	Skills	Training	4.1 Instructions on how to perform the behaviour

Greenwell K, *et al. BMJ Open* 2022; 12:e056867. doi: 10.1136/bmjopen-2021-056867

**Barriers/*facilitator* to target behaviour**	**Intervention components**	**Target construct (BCW)**	**Theoretical domain (TDF)**	**Intervention function (BCW)**	**Behaviour Change Technique (using BCTv1)**
	emollients have different constituents, smells, and feel)<br>• Provide advice on disguising emollients when around others/away from home (e.g. putting emollient in smaller containers)				
	The 2-week challenge:<br>• Provide an emollient chart to allow YP to record how their skin is each day after applying emollients and how they have found the emollients (e.g. side effects, texture)	Reflective Motivation	Beliefs about consequences	Education; Persuasion	5.1 Information about health consequences<br>2.4 Self-monitoring of outcomes of behaviour
Concerns about the psychosocial impact of emollients (e.g. feeling self-conscious) [QSR; QI]	• Provide strategies to reduce the psychosocial impact of emollients (e.g. feeling less self-conscious)<br>• Provide quotes from YPs explaining how they overcame some of the psychosocial consequences of emollients	Psychological capability; Reflective Motivation; Social opportunity	Beliefs about consequences; Beliefs about capabilities; Knowledge; Skills; Social influences	Education; Persuasion; Training; Modelling	5.3 Information about social and environmental consequences<br>5.6 Information about emotional consequences<br>6.2 Social comparison<br>6.3 Information about others' approval<br>9.1 Credible source
Inconvenience of using emollients when away from home [QSR; QI]	• Provide advice on using emollients when away from home (e.g. requesting smaller tubes from health professional)	Physical Opportunity	Environmental context and resources	Environmental restructuring	12.1 Restructuring the physical environment
Inconvenience of emollients rubbing off (e.g. on clothes and bed sheets) [QSR; QI]	• Provide advice on how to choose the right emollient and avoiding them rubbing off (e.g. put on loose clothing, allowing adequate time for absorption, leaving it to dry in a warm room)	Psychological capability	Skills	Training	4.1 Instructions on how to perform the behaviour

**Barriers/*facilitator* to target behaviour**	**Intervention components**	**Target construct (BCW)**	**Theoretical domain (TDF)**	**Intervention function (BCW)**	**Behaviour Change Technique (using BCTv1)**
Belief that emollients are time-consuming to apply and to find the right one/competing time pressures [QSR; QI]<br><br>Forgetting to apply emollients [QI]<br><br>*Having an emollient routine [QI]*	• Provide information on how to integrate emollient use into everyday life<br>• Reassure YP that applying emollients should not be time-consuming<br>• Advise YP to plan when they will apply their emollients (i.e. ensure they allocate time)	Psychological capability; Reflective Motivation	Knowledge; Skills; Goals	Education; Training; Enablement	1.4 Action planning<br>4.1 Instructions on how to perform the behaviour<br>5.3 Information about social and environmental consequences
	The 2-week challenge:<br>• Suggest YP to apply their emollient daily<br>• Suggest YP plan when they will apply their emollients<br>• Suggest YP apply their creams at the same time in the same context each day<br>• Allow YP to choose how many times per day they aim to apply their emollients and suggest they choose a particular time of the day or situation (e.g. after showering) to apply.<br>• Provide an emollient chart to record whether they have used their emollients at their agreed times.	Reflective motivation; Psychological capability	Goals; Skills; Behavioural regulation	Enablement; Training	1.1 Goal setting (behaviour)<br>1.4 Action planning<br>2.3 Self-monitoring of behaviour<br>8.1 Behavioural practice/rehearsal<br>8.3 Habit formation
	• Allow users to set up regular reminders to apply emollients by email or text messages and decide on the frequency of these reminders<br>• Provide an emollient chart that acts as a reminder by suggesting YP record whether they have used their	Physical opportunity	Environmental context and resources	Environmental structuring	7.1 Prompts/cues

**Barriers/*facilitator* to target behaviour**	**Intervention components**	**Target construct (BCW)**	**Theoretical domain (TDF)**	**Intervention function (BCW)**	**Behaviour Change Technique (using BCTv1)**
	emollients at their agreed times [2-week challenge]				
Belief that finding an emollient that works best for you is inconvenient, confusing and time-consuming [QSR; QI]	• Acknowledge how frustrating and time-consuming this process can be<br>• Provide quotes from YP emphasising the importance of finding the right emollient	Psychological capability; Reflective motivation; Social opportunity	Beliefs about consequences; Beliefs about capabilities; Knowledge; Social Influences	Education; Persuasion; Modelling	5.1 Information about health consequences<br>6.2 Social comparison<br>6.3 Information about others' approval<br>9.1 Credible source
Belief that you don't need to apply the emollient every day or only when you need it/eczema is bad [QI]	• Provide information on how often to apply emollients and the rationale for doing this	Psychological capability; Reflective Motivation	Knowledge; Skills	Education; Training	4.1 Instructions on how to perform the behaviour<br>5.1 Information about health consequences
Lack of skills regarding how to apply emollients/ low self-efficacy [QSR]	• Provide instructions on how to correctly applying emollients, including how much emollient to apply	Psychological capability	Skills	Training	4.1 Instructions on how to perform the behaviour
Ran out of emollients [EO]<br><br>*Stocking up on emollient [QI]*	• Provide advice on how to obtain more emollients/avoid running out	Physical Opportunity	Environmental context and resources	Environmental structuring	12.5 Adding objects to the environment
Cost of emollients [QI]	• Provide advice about financial benefits YP can apply for or strategies for reducing the cost of emollients	Physical Opportunity; Psychological Capability; Reflective Motivation	Environmental context and resources; Knowledge; Beliefs about consequences	Environmental structuring; Education; Persuasion	5.3 Information about social and environmental consequences<br>12.5 Adding objects to the environment
Difficulties in getting health professionals to	• Provide advice on how to prepare for appointments with health professionals	Psychological Capability	Skills	Training	4.1 Instructions on how to perform the behaviour

**Barriers/*facilitator* to target behaviour**	**Intervention components**	**Target construct (BCW)**	**Theoretical domain (TDF)**	**Intervention function (BCW)**	**Behaviour Change Technique (using BCTv1)**
prescribe different emollients [QI]<br><br>*Preparing and researching for consultations [QI]*					
**Target behaviour: Improved use of topical corticosteroids (TCS) or Topical Calcineurin Inhibitors (TCI)**					
Belief that TCSs/TCIs are not effective enough for managing flare-ups (e.g. provide only temporary relief before their eczema returned) [QI]	• Provide persuasive and credible information about the effectiveness of TCS/TCIs, including scientific evidence, user quotes and videos<br>• Provide rationale for how TCS/TCIs control eczema<br>• Provide advice on whether emollients are also needed	Psychological capability; Reflective motivation; Social opportunity	Beliefs about consequences; Knowledge; Social Influences	Education; Persuasion; Modelling	5.1 Information about health consequences<br>6.2 Social comparison<br>6.3 Information about others' approval<br>9.1 Credible source
	• Provide advice on how to choose an effective TCS/TCIs	Psychological capability	Skills	Training	4.1 Instructions on how to perform the behaviour
Concerns about the long-term safety of TCSs [QSR; QI; EO]	• Provide persuasive and credible information about the safety of TCSs/TCIs (e.g. skin thinning, wrinkling, dependency), including scientific evidence, user quotes and videos	Psychological capability; Reflective motivation; Social opportunity	Beliefs about consequences; Knowledge; Social Influences	Education; Persuasion; Modelling	5.1 Information about health consequences<br>6.2 Social comparison<br>6.3 Information about others' approval<br>9.1 Credible source
Concerns about the immediate side effects (e.g. stinging) of TCSs/TCIs [QI]	• Provide reassuring advice about the temporary nature of side effects (e.g. stinging) and how to choose the right TCS/TCIs (i.e. no side effects)	Psychological capability; Reflective motivation;	Skills; Beliefs about consequences; Knowledge	Education; Persuasion; Training	4.1 Instructions on how to perform the behaviour<br>5.1 Information about health consequences
Uncertainty regarding when to start and finish TCSs/TCIs [QI]	• Provide information on when to apply TCSs/TCIs and for how long	Psychological capability	Skills	Training	4.1 Instructions on how to perform the behaviour

Greenwell K, *et al. BMJ Open* 2022; 12:e056867. doi: 10.1136/bmjopen-2021-056867

**Barriers/*facilitator* to target behaviour**	**Intervention components**	**Target construct (BCW)**	**Theoretical domain (TDF)**	**Intervention function (BCW)**	**Behaviour Change Technique (using BCTv1)**
Lack of skills regarding how to apply TCSs/TCIs / low self-efficacy [EO]	• Provide instructions on how to correctly apply TCSs/TCIs, including how much to apply	Psychological capability	Skills	Training	4.1 Instructions on how to perform the behaviour
Belief that using more TCS than prescribed will conceal their eczema or make their eczema flare-up pass quicker [QI]	• Provide information on how much TCS/TCIs to apply	Psychological capability	Skills	Training	4.1 Instructions on how to perform the behaviour
Uncertainty regarding the difference between steroids [QI]	• Provide information on what type of steroids are available and which ones they should use on different body parts and why	Psychological capability	Skills	Training	4.1 Instructions on how to perform the behaviour
Ran out of TCS/TCIs /not being able to get hold of their preferred TCS (e.g. out of stock) [QI]<br><br>*Stocking up on TCS/TCIs [QI]*	• Provide information on how to obtain more TCS/TCIs and avoid running out	Physical Opportunity	Environmental context and resources	Environmental structuring	12.5 Adding objects to the environment
Belief that health professionals (including pharmacists) are reluctant or hesitant to prescribe TCSs/the right potency [QI]	• Provide advice on how to talk to health professionals<br>• Provide advice on how to choose an effective TC/TCIs S (e.g. list of available TCSs/TCIs)	Psychological capability	Skills	Training	4.1 Instructions on how to perform the behaviour

Greenwell K, *et al. BMJ Open* 2022; 12:e056867. doi: 10.1136/bmjopen-2021-056867

**Barriers/*facilitator* to target behaviour**	**Intervention components**	**Target construct (BCW)**	**Theoretical domain (TDF)**	**Intervention function (BCW)**	**Behaviour Change Technique (using BCTv1)**
	• Provide explanation of how the health professional decides on the TCS/TCI prescription				
Cost of TCSs/TCIs [QI]	• Provide advice about financial benefits YP can apply for or strategies for reducing the cost of TCSs/TCIs	Psychological capability; Reflective motivation	Beliefs about consequences; Knowledge	Education; Persuasion	5.3 Information about social and environmental consequences
**Target behaviour: Management of irritants and triggers**					
Lack of knowledge regarding common irritants and triggers [EO]	• Provide information on common irritants and triggers<br>• Provide information on misconceptions (e.g. food allergies)	Psychological capability; Reflective Motivation	Beliefs about consequences; Knowledge; Skills	Education; Persuasion; Training	4.1 Instructions on how to perform the behaviour<br>5.1 Information about health consequences
Belief that you can't avoid some triggers (e.g. stress)/Belief that the benefits of avoidance (e.g. avoiding a brief flare-up) do not outweigh the costs (e.g. not being able to go swimming) [QI]	• Where appropriate, provide advice on how to minimise the effects of irritants/triggers (e.g. apply emollients before and/or afterwards)<br>• Where appropriate, provide advice on how to avoid or reduce contact with certain irritants/triggers (e.g. soaps, high temperatures, sweat), including when away from home	Psychological capability; Reflective Motivation	Beliefs about consequences; Knowledge; Skills	Education; Persuasion; Training	4.1 Instructions on how to perform the behaviour<br>5.1 Information about health consequences

Greenwell K, *et al. BMJ Open* 2022; 12:e056867. doi: 10.1136/bmjopen-2021-056867

**Barriers/*facilitator* to target behaviour**	**Intervention components**	**Target construct (BCW)**	**Theoretical domain (TDF)**	**Intervention function (BCW)**	**Behaviour Change Technique (using BCTv1)**
**Target behaviour: Reduce scratching**					
Belief that scratching is not that bad [EO]	• Explain itch-scratch cycle and that scratching makes itch/eczema worse	Psychological capability; Reflective Motivation	Beliefs about consequences; Knowledge	Education; Persuasion	5.1 Information about health consequences<br>5.6 Information about emotional consequences
Feeling of itchiness and desire for instant relief [QI]<br><br>*Wearing appropriate clothes [QI]; Keeping cool [QI]; Emollient use [QI]; Antihistamine [QI]; Tapping [QI]; Keeping nails short/wear gloves [QI]*	• Provide information on the factors that make scratching more likely (e.g. hot temperature, lack of sleep)<br>• Provide advice on reducing itchiness (e.g. keeping cool, emollient use, tapping the area, clench fist, wearing clothes with long arms and legs, put emollient in fridge to keep cool).<br>• Provide advice on sleep management.<br>• Provide techniques to prevent the negative consequences of scratching (e.g. keep nails short, wear gloves) and how to deal with the annoyance of others telling them to stop scratching (e.g. ask people to suggest things they can do, like tap skin, instead).	Physical Capability; Psychological Capability	Environmental context and resources; Skills; Behavioural Regulation	Training; Education; Enablement; Environmental restructuring	4.1 Information on how to perform a behaviour<br>4.2 Information about antecedents<br>12.3 Avoidance/reducing exposure to cues for behaviour<br>12.5 Adding objects to the environment<br>12.6 Body changes
Scratching is a habit [QI]<br><br>*Distraction techniques [QI]*	• Provide techniques to raise awareness of scratching (e.g. record instances of scratching)<br>• Provide techniques to stop scratching (e.g. distraction, relaxation techniques, replace scratching with an alternative behaviour such as clenching fists).	Psychological Capability; Automatic Motivation; Physical Opportunity	Environmental context and resources; Skills; Behavioural Regulation	Training; Enablement; Environmental restructuring	2.3 Self-monitoring of behaviour<br>4.1 Information on how to perform a behaviour<br>8.2 Behaviour substitution<br>8.4 Habit reversal<br>12.4 Distraction<br>12.5 Adding objects to the environment
**Target behaviour: Engaging in emotional management techniques**					

Greenwell K, *et al. BMJ Open* 2022; 12:e056867. doi: 10.1136/bmjopen-2021-056867

**Barriers/*facilitator* to target behaviour**	**Intervention components**	**Target construct (BCW)**	**Theoretical domain (TDF)**	**Intervention function (BCW)**	**Behaviour Change Technique (using BCTv1)**
Belief that emotional management techniques will do little to control their eczema or help with difficult emotions [EO]/ Belief that stress (e.g. during exam time) can't be avoided [QI]<br><br>*Belief that emotions affect eczema [QI]*	• Explain the necessity of emotional management techniques for promoting engagement with the other behaviours and provide evidence that they are effective for dealing with difficult emotions	Psychological capability; Reflective Motivation	Beliefs about consequences; Knowledge	Education; Persuasion	5.1 Information about health consequences<br>5.6 Information about emotional consequences
	• Provide user quotes demonstrating how emotional management techniques helped other users to take control of their eczema	Psychological capability; Reflective motivation; Social opportunity	Beliefs about consequences; Knowledge; Social Influences	Education; Persuasion; Modelling	5.1 Information about health consequences<br>6.2 Social comparison<br>6.3 Information about others' approval<br>9.1 Credible source
Lack of understanding regarding how to do the emotional management techniques [EO]	• Provide guidance on how to do the emotional management techniques	Psychological capability	Skills	Training	4.1 Instructions on how to perform the behaviour
	• Provide guided audio recordings of emotional management exercises	Physical opportunity	Environmental context and resources	Environmental structuring	12.5 Adding objects to the environment
Lack of confidence in ability to practice emotional management techniques [EO]	• Provide YP quotes demonstrating how easy it was for other users to practice the emotional management techniques	Psychological capability; Reflective motivation; Social opportunity	Beliefs about consequences; Beliefs about capabilities; Knowledge; Social Influences	Education; Persuasion; Modelling	6.2 Social comparison<br>6.3 Information about others' approval<br>9.1 Credible source

Greenwell K, *et al. BMJ Open* 2022; 12:e056867. doi: 10.1136/bmjopen-2021-056867
