# Supplementary material for: Eczema Care Online: development and qualitative optimisation of an online behavioural intervention to support self-management in young people with eczema
Source: BMJ Open. 2022 Apr 19;12(4):e056867. doi: 10.1136/bmjopen-2021-056867 (PMC9021764; doi:10.1136/bmjopen-2021-056867)

Supplementary Material 2: Screenshot of page in introductory module providing key behavioural messages on topical treatments

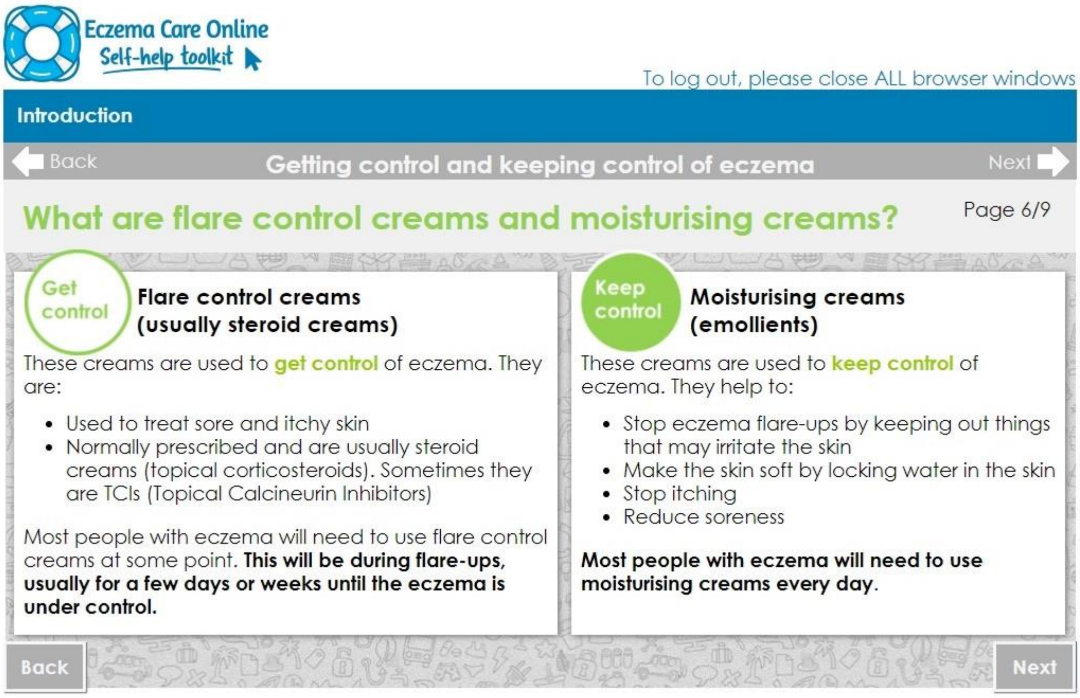

Supplement: Supplementary data [file bmjopen-2021-056867supp002.pdf]
