# Supplementary material for: Eczema Care Online: development and qualitative optimisation of an online behavioural intervention to support self-management in young people with eczema
Source: BMJ Open. 2022 Apr 19;12(4):e056867. doi: 10.1136/bmjopen-2021-056867 (PMC9021764; doi:10.1136/bmjopen-2021-056867)
Supplement: Supplementary data [file bmjopen-2021-056867supp003.pdf]

Supplementary Material 3: Screenshot of video explaining how eczema affects the skin barrier to provide a better understanding of eczema and a rationale for how topical treatments work

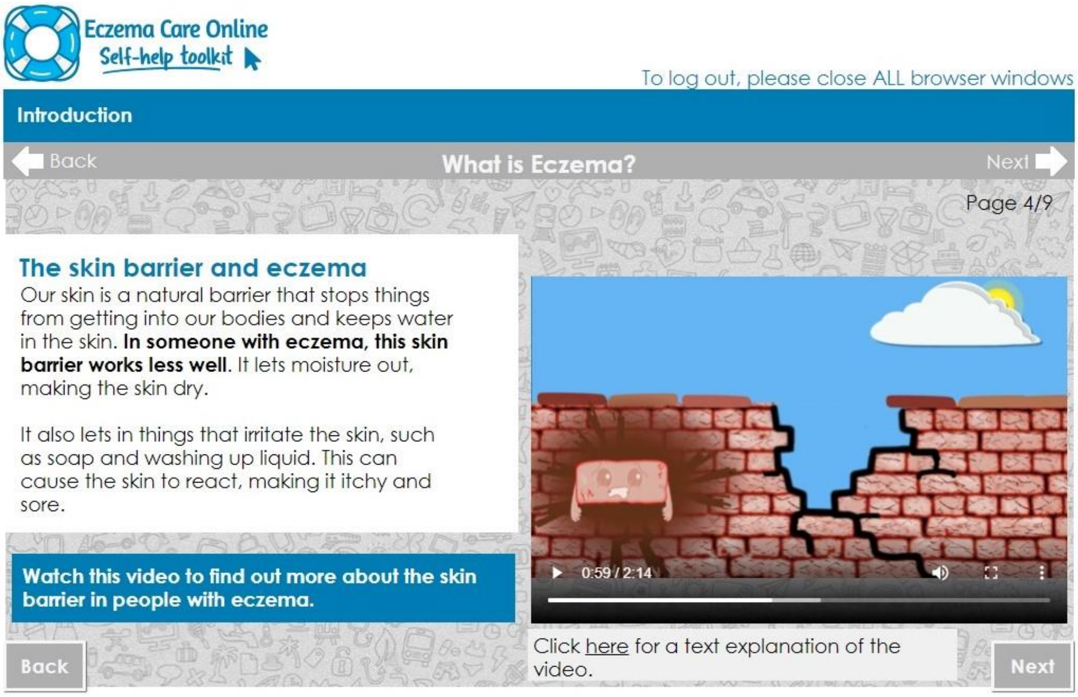

Greenwell K, *et al. BMJ Open* 2022; 12:e056867. doi: 10.1136/bmjopen-2021-056867
