# Supplementary material for: Eczema Care Online: development and qualitative optimisation of an online behavioural intervention to support self-management in young people with eczema
Source: BMJ Open. 2022 Apr 19;12(4):e056867. doi: 10.1136/bmjopen-2021-056867 (PMC9021764; doi:10.1136/bmjopen-2021-056867)
Supplement: Supplementary data [file bmjopen-2021-056867supp004.pdf]

Supplementary Material 4: Screenshot of simple eczema assessment and subsequent feedback on which of the core treatment modules (emollients or topical corticosteroids) would be most relevant depending on whether they were currently experiencing an eczema flare-up

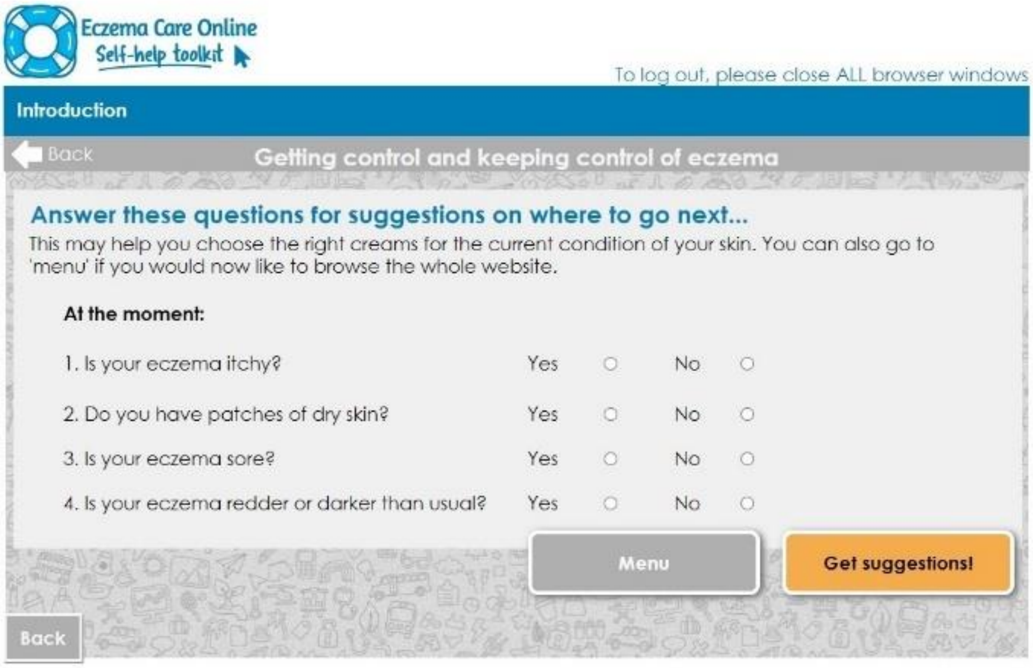

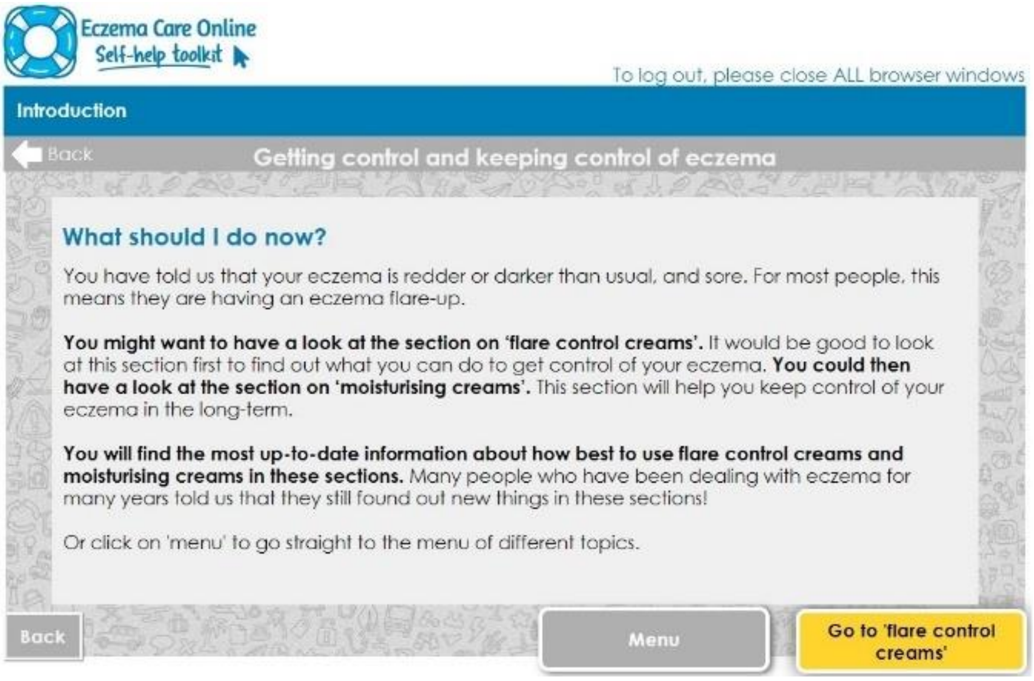

Greenwell K, *et al. BMJ Open* 2022; 12:e056867. doi: 10.1136/bmjopen-2021-056867
