# Supplementary material for: Eczema Care Online: development and qualitative optimisation of an online behavioural intervention to support self-management in young people with eczema
Source: BMJ Open. 2022 Apr 19;12(4):e056867. doi: 10.1136/bmjopen-2021-056867 (PMC9021764; doi:10.1136/bmjopen-2021-056867)

Supplementary Material 5: Screenshot of page explaining the menu of different information topics and quotes from other young people with eczema

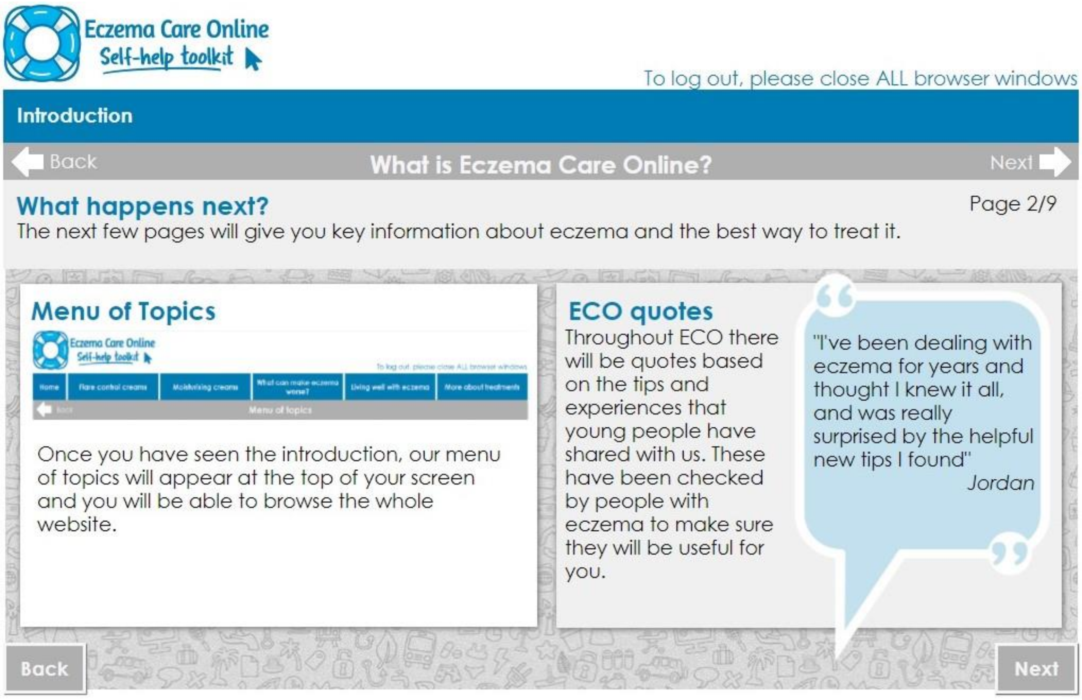

Supplement: Supplementary data [file bmjopen-2021-056867supp005.pdf]
