# Supplementary material for: Eczema Care Online: development and qualitative optimisation of an online behavioural intervention to support self-management in young people with eczema
Source: BMJ Open. 2022 Apr 19;12(4):e056867. doi: 10.1136/bmjopen-2021-056867 (PMC9021764; doi:10.1136/bmjopen-2021-056867)
Supplement: Supplementary data [file bmjopen-2021-056867supp006.pdf]

Supplementary Material 6: Description of Eczema Care Online (ECO) for Young People in accordance with the Template for Intervention Description and Replication (TIDieR) checklist

- 1. BRIEF NAME: Eczema Care Online (ECO) for Young People
- 2. WHY: Online interventions could potentially be used to support young people with eczema; however, research developing such interventions has been limited and systematic reviews show the need for systematically developed evidence-based interventions to support self-management of eczema.
- 3 & 4. WHAT (materials and procedures): ECO for Young People is an online behavioural intervention for young people with eczema aged 13-25 years with mild to severe eczema. It aims to reduce eczema severity, targeting 5 key behaviours: (1) Increase their use of emollients to maintain skin hydration and prevent flare-ups; (2) Improve their use of TCS or TCIs through reactive applications of these treatments in response to flare-ups or, where appropriate, regular intermittent ("weekend") preventative applications of TCS/TCI if emollients are insufficient as maintenance therapy; (3) Improve their management of irritants and triggers; (4) Improve their emotional management; and (5) Reduce scratching. See the table below for an overview of the modules included in ECO for Young People. For more detail about specific intervention techniques, see the behavioural analysis in Supplementary Material 1.

Table 1: Overview of the key intervention components of ECO for Young People

Menu	Modules	Description
Home Page	N/A	• Signposting to key modules that young people haven't accessed yet<br>• Quick links to interactive content e.g. videos
Flare control creams	• Flare control cream video<br>• What are they?<br>• Are they safe?<br>• Common questions<br>• When and how do I use them?<br>• How can I find the right cream?<br>• Golden rules	• Evidence-based education and messages to increase motivation and competence to use topical corticosteroids (TCS), including; evidence of safety and support for skills development e.g. videos/photos showing use of TCS
Moisturising creams	• Moisturising creams video<br>• What are they and how to they help?<br>• Are they safe?	• Evidence-based education and messages to increase motivation and competence to use emollients, including; evidence of safety and

	• Common Concerns<br>• When and how do I use them?<br>• How do I find the right cream?<br>• Take the two-week challenge<br>• Golden rules	support for skills development e.g. videos/photos showing how to use emollients
What can make eczema worse	• Living with eczema video<br>• Bathing, showering, and washing clothes<br>• Cosmetics, make-up and shaving<br>• Diet and allergies<br>• Weather and holidays<br>• Swimming and physical activity	• Evidence-based education and messages to increase motivation and competence in identifying key eczema triggers and managing them, including practical tips
Living well with eczema	• Beat the itch<br>• Sleep<br>• Stress and eczema<br>• Studying and work<br>• Eczema and money	• Evidence-based education and messages to increase motivation and competence to manage some of the symptoms and psychosocial impacts of eczema e.g. itching and scratching, disturbed sleep, stress, including techniques to manage them
More about treatments	• Talking to health professionals<br>• Infections<br>• Topical Calcineurin Inhibitors<br>• Other treatments<br>• Other resources	• Evidence-based education and messages to increase motivation and competence to manage other aspects of treatments e.g. consulting with health professionals and other treatments

- 5. WHO PROVIDED: ECO for Young People is provided direct to patients via their GP surgeries.
- 6. HOW: ECO for Young People is entirely online, although users are encouraged to contact their GP, pharmacist or nurse, for example, to obtain or change their treatments, or to treat potential infections.
- 7. WHERE: ECO for Young People is intended for any young person (aged 13-25) with mild to severe eczema. It can be implemented in primary or secondary care. The intervention is online and designed to be accessed using computers, tablets and phones via a web-browser.
- 8. WHEN and HOW MUCH: For the randomised controlled trial study, young people are given access to the intervention if their GP identifies that they have eczema that has required treatment in the last 12 months. Once registered young people can use the online intervention as much or as little as they like.

- 9. TAILORING: Young people have access to all the modules on ECO, however the website uses signposting based on four questions about the current state of their eczema. They will then be recommended either the module on emollients or flare control creams module depending on whether it appears they are currently experiencing an eczema flare-up. Key modules will also be recommended, one or two at a time, on the intervention home page if they haven't been viewed yet. These modules/menus are: 1) moisturising creams; 2) flare control creams, 3) bathing, showering and washing clothes; 4) beat the itch, 5) stress and eczema.
- 10. MODIFICATIONS: An additional email was circulated in response to COVID-19 that provided further advice about handwashing.
- 11. HOW WELL: The intervention is currently being tested in a randomised control trial with young people with eczema (ISRCTN79282252). Information about the results of the randomised controlled trial, including both quantitative and qualitative data, will be presented in future publications.
