# Supplementary material for: Eczema Care Online: development and qualitative optimisation of an online behavioural intervention to support self-management in young people with eczema
Source: BMJ Open. 2022 Apr 19;12(4):e056867. doi: 10.1136/bmjopen-2021-056867 (PMC9021764; doi:10.1136/bmjopen-2021-056867)
Supplement: Author's manuscript [file bmjopen-2021-056867.draft_revisions.pdf]

BMJ Open is committed to open peer review. As part of this commitment we make the peer review history of every article we publish publicly available.

When an article is published we post the peer reviewers' comments and the authors' responses online. We also post the versions of the paper that were used during peer review. These are the versions that the peer review comments apply to.

The versions of the paper that follow are the versions that were submitted during the peer review process. They are not the versions of record or the final published versions. They should not be cited or distributed as the published version of this manuscript.

BMJ Open is an open access journal and the full, final, typeset and author-corrected version of record of the manuscript is available on our site with no access controls, subscription charges or pay-per-view fees (http://bmjopen.bmj.com).

If you have any questions on BMJ Open's open peer review process please email info.bmjopen@bmj.com

BMJ Open

Eczema Care Online: development and qualitative optimisation of an online behavioural intervention to support self-management in young people with eczema

Journal:	*BMJ Open*
Manuscript ID	bmjopen-2021-056867
Article Type:	Original research
Date Submitted by the Author:	30-Aug-2021
Complete List of Authors:	Greenwell, Kate; University of Southampton<br>Roberts, Amanda; University of Nottingham<br>Le Roux, Emma; University of Hertfordshire<br>Jones, Hannah; University of Southampton<br>Whittaker, Emilia; University of Southampton<br>Ghio, Daniela; University of Salford<br>Sivyer, Katy; University of Southampton School of Psychology; University of Portsmouth, Department of Psychology<br>Steele, Mary; University of Southampton School of Psychology<br>Teasdale, Emma; University of Southampton<br>Ridd, Matthew; University of Bristol Faculty of Health Sciences, Population Health Sciences<br>Chalmers, Joanne; University of Nottingham, Centre of Evidence Based Dermatology<br>Lawton, Sandra; Rotherham NHS Foundation Trust, Dermatology Department<br>Langan, Sinead; London School of Hygiene & Tropical Medicine<br>Cowdell, Fiona; Birmingham City University, Faculty of Health Education and Life Sciences<br>Wilczynska, Sylvia; University of Southampton, School of Primary Care, Population Health and Medical Education,<br>Williams, HC; University of Nottingham<br>Thomas, Kim; University of Nottingham, Dermatology<br>Yardley, Lucy; University of Southampton, Psychology; University of Bristol, School of Health Sciences<br>Muller, Ingrid; University of Southampton, School of Primary Care, Population Health and Medical Education<br>Santer, Miriam; University of Southampton, Primary Care & Population Sciences
Keywords:	Eczema < DERMATOLOGY, QUALITATIVE RESEARCH, Paediatric dermatology < DERMATOLOGY, PRIMARY CARE

SCHOLARONE™
Manuscripts

I, the Submitting Author has the right to grant and does grant on behalf of all authors of the Work (as defined in the below author licence), an exclusive licence and/or a non-exclusive licence for contributions from authors who are: i) UK Crown employees; ii) where BMJ has agreed a CC-BY licence shall apply, and/or iii) in accordance with the terms applicable for US Federal Government officers or employees acting as part of their official duties; on a worldwide, perpetual, irrevocable, royalty-free basis to BMJ Publishing Group Ltd ("BMJ") its licensees and where the relevant Journal is co-owned by BMJ to the co-owners of the Journal, to publish the Work in this journal and any other BMJ products and to exploit all rights, as set out in our licence.

The Submitting Author accepts and understands that any supply made under these terms is made by BMJ to the Submitting Author unless you are acting as an employee on behalf of your employer or a postgraduate student of an affiliated institution which is paying any applicable article publishing charge ("APC") for Open Access articles. Where the Submitting Author wishes to make the Work available on an Open Access basis (and intends to pay the relevant APC), the terms of reuse of such Open Access shall be governed by a Creative Commons licence – details of these licences and which Creative Commons licence will apply to this Work are set out in our licence referred to above.

Other than as permitted in any relevant BMJ Author's Self Archiving Policies, I confirm this Work has not been accepted for publication elsewhere, is not being considered for publication elsewhere and does not duplicate material already published. I confirm all authors consent to publication of this Work and authorise the granting of this licence.

For peer review only

Title: Eczema Care Online: development and qualitative optimisation of an online behavioural intervention to support self-management in young people with eczema

Kate Greenwell[1*], Daniela Ghio[2], Katy Sivyer[1,3], Mary Steele[1], Emma Teasdale[4], Matthew J. Ridd[5], Amanda Roberts[6], Joanne Chalmers[6], Sandra Lawton[7], Sinéad M Langan[8], Fiona Cowdell[9], Emma Le Roux[10], Sylvia Wilczynska[4], Hannah Jones[11], Emilia Whittaker[11], Hywel C Williams[6], Kim S Thomas[6], Lucy Yardley[1,12], Miriam Santer[4], Ingrid Muller[4]

[1]Centre for Clinical and Community Applications of Health Psychology, Faculty of Environmental and Life Sciences, University of Southampton, Southampton, UK; [2]Psychology, School of Health and Society, University of Salford, Manchester, UK [3]Department of Psychology, Faculty of Science and Health, University of Portsmouth, Portsmouth, UK; [4]School of Primary Care, Population Sciences and Medical Education, Faculty of Medicine, University of Southampton, Southampton, UK; [5]Population Health Sciences, University of Bristol, Bristol, UK; [6]Centre of Evidence Based Dermatology, School of Medicine, University of Nottingham, Nottingham, UK; [7]Department of Dermatology, Rotherham NHS Foundation Trust, Rotherham, UK; [8]Faculty of Epidemiology and Population Health, London School of Hygiene and Tropical Medicine, London, UK; [9]Faculty of Health, Education and Life Sciences, Birmingham City University, Birmingham, UK; [10]School of Life and Medical Sciences, University of Hertfordshire, Hatfield, UK; [11]Faculty of Medicine, University of Southampton, Southampton, UK; [12]School of Experimental Psychology, University of Bristol, Bristol, UK.

***Corresponding author**: Kate Greenwell, University of Southampton, Highfield Campus, Southampton, SO17 1BJ, UK; Email: K.Greenwell@soton.ac.uk

For peer review only

Abstract

Objectives: To describe the development of Eczema Care Online (ECO), an online behaviour change intervention for young people with eczema (Phase 1); and explore and optimise the acceptability of ECO among this target group using think-aloud interviews (Phase 2).

Methods: Theory-, evidence-, and person-based approaches to intervention development were used. In Phase 1, a qualitative systematic review and qualitative interviews developed an in-depth understanding of the needs and challenges of young people with eczema. Guiding principles highlighted key intervention design objectives and features to address needs of this target group to maximise user engagement. Behavioural analysis and logic modelling developed ECO's hypothesised programme theory. In Phase 2, qualitative think-aloud interviews were carried out with 28 young people with eczema and the intervention was optimised based on their feedback.

Results: The final intervention aimed to reduce eczema severity by supporting treatment use (emollients, topical corticosteroids/topical calcineurin inhibitors), management of irritants/triggers, emotional management; and reducing scratching. Generally, young people expressed positive views of intervention content and design in think-aloud interviews. Quotes and stories from other young people with eczema and ECO's focus on living with eczema (not just topical treatments) were valuable for normalising eczema. Young people believed ECO addressed knowledge gaps they had from childhood and the safety information about topical corticosteroids was reassuring. Negative feedback was used to modify ECO.

Conclusions: A prototype of the ECO intervention was developed using rigorous and complementary intervention development approaches. Subsequent think-aloud interviews helped optimise the intervention and demonstrated ECO is likely to be acceptable to this

For peer review only - http://bmjopen.bmj.com/site/about/guidelines.xhtml

target group and provided support for our guiding principles including key design objectives and features to consider when developing interventions for this population. A randomised controlled trial and process evaluation of the intervention is underway to assess effectiveness and explore user engagement with the intervention's behavioural goals.

Article Summary

Strengths and limitations of this study

- Our rigorous development using complementary theory-, evidence-, and person-based approaches to intervention development helped ensure the intervention was acceptable and engaging to our sample of young people with eczema.
- Our multidisciplinary intervention development group, including Patient and Public Involvement, ensured that the content was evidence-based, that advice was feasible, and that the perspectives of people living with eczema were considered throughout the whole development process.
- Although we were able to recruit a good range of ages, genders and eczema severities for the think-aloud interview study, a large proportion of the sample were university students, so were likely to have high levels of health and computer literacy.
- As think-aloud interviews explore participants' immediate reactions to the intervention, they cannot tell us how people would use the intervention over time or explore engagement with the wider behavioural goals.

Manuscript word count: 4133

For peer review only - http://bmjopen.bmj.com/site/about/guidelines.xhtml

For peer review only

Introduction

Eczema is a common skin condition that usually begins in childhood,[1] but for many the symptoms (dry, sore, itchy skin) can persist into adolescence and adulthood.[2] Eczema management focuses on identification and avoidance of irritants/triggers that may exacerbate eczema symptoms; the regular use of emollients to restore the skin's barrier function; and topical corticosteroids (or Topical Calcineurin Inhibitors [TCIs] for those with moderate to severe eczema) use to treat flare-ups.[3]

Eczema management can be particularly challenging in adolescence and early adulthood. Young people report a lack of knowledge regarding their eczema and treatments, and advice provided can often conflict with their own eczema experiences and a competing desire to maintain a 'normal' adolescent life.[4–8] These factors may explain why adherence to topical treatments presents challenges for this age group and many report not using their treatments as prescribed.[6]

Systematic reviews of self-management interventions for people with eczema[9,10] identified that, although a few studies evaluated interventions have been developed for parents/carers of children with eczema, only two studies evaluated interventions for children and adolescents.[11,12] Both interventions were delivered face-to-face and only one reported tailoring their intervention to this age group.[11] To address this research gap and the need to improve self-management support for people living with eczema, the Eczema Care Online (ECO) programme aimed to develop two online behavioural interventions: one for young people with eczema (13-25 years) and one for parents and carers of children (0-12 years) with eczema (www.nottingham.ac.uk/eco).[13,14]

First, we aimed to describe the development of the ECO intervention for young people with eczema using complementary person-, theory-, and evidence-based approaches (Phase 1).

For peer review only - http://bmjopen.bmj.com/site/about/guidelines.xhtml

Guidelines for developing complex interventions emphasise that interventions should be informed by reviews of the current evidence-base, appropriate theory, and an in-depth understanding of the context in which the intervention will be implemented.[15] This article highlights key psychosocial needs of young people with eczema and intervention features to consider when developing behavioural interventions for this group. Second, we aimed to carry out think-aloud interviews to explore and optimise the acceptability of the ECO intervention among young people with eczema (Phase 2).

Intervention development methodology

We used theory-, evidence-, and person-based approaches to develop the online intervention.[16–18] The Person-Based Approach to intervention development uses iterative qualitative research to understand and accommodate the perspectives of the intervention's target group.[19]

Intervention development was carried out in two phases. In Phase 1, we collated and synthesised evidence relating to patient behaviours that are likely to reduce eczema severity and the perspectives of young people with eczema (person- and evidence-based approach). This evidence guided decisions regarding the intervention's target behaviours and provided us with an in-depth understanding of the key issues, needs, and behavioural challenges of this target group. Theory-based approaches (behavioural analysis and logic modelling) were used to develop and illustrate the intervention's hypothesised programme theory, that is, the hypothesised mechanisms of action by which the intervention components exerts their effects.[15,20] In Phase 2, we carried out iterative qualitative think-aloud interviews to gather user feedback on the intervention prototype and optimise it based on this feedback.[21] The methods and results for each phase are reported below.

For peer review only - http://bmjopen.bmj.com/site/about/guidelines.xhtml

For peer review only

Both phases were guided by a multidisciplinary intervention development group, which comprised 18 members including Patient and Public Involvement (PPI), dermatologists, a nurse consultant, researchers with an interest in eczema, GPs, health psychologists, and experts in intervention development and long-term conditions in adolescents. Through regular meetings and reviewing documents, this group guided the design of the research, helped with the interpretation of the research findings, and provided detailed feedback on the intervention plan, written content, and prototype. The intervention development process is illustrated in Figure 1. Ethical approval for this research was granted by Wales REC 7 Ethics Committee (REC 17/WA/0329).

Patient and Public Involvement

Two mothers of children and young people with eczema (one of whom had eczema herself) were part of our multidisciplinary intervention development group. Both were co-applicants on the research grant application, helping to identify the research topic and develop research questions. We also sought additional PPI feedback on the intervention content from two young people with eczema and a panel of PPI contributors with an interest in skin research, most of whom had experience of eczema, and some were aged 18-25. We discuss the specific contributions of the PPI to intervention development throughout this manuscript. Two young people reviewed the study participant information sheet to check comprehension. AR discussed and provided feedback on our interpretations of the findings and this manuscript. She continues to help us to disseminate our research findings among her wide-reaching patient networks and via social media.

Phase 1: Intervention planning

Phase 1 Methods

For peer review only - http://bmjopen.bmj.com/site/about/guidelines.xhtml

Phase 1 comprised of three steps: (1) Defining the intervention target behaviours; (2) Collating and synthesising evidence relating to the perspectives of young people with eczema; and (3) Creating an intervention plan.

1.1 Defining the intervention target behaviours

The multidisciplinary intervention development group agreed the intervention's key target behaviours through consideration of the evidence-base for effective eczema treatments, clinical guidance on eczema management and recommendations around what would be feasible and acceptable to implement through an online intervention.[3,22,23]

1.2 Collating and synthesising evidence relating to the perspectives of young people with eczema

First, we undertook a systematic review and thematic synthesis of the views and experiences of eczema and/or eczema self-care of people with eczema and parents or carers of children with eczema.[7] Qualitative studies focusing on the views and experiences of eczema and eczema treatments, and barriers and facilitators to eczema self-management were included. The review identified 39 papers (reporting 32 studies; 9 including young people in the sample).

Second, we carried out a secondary analysis of interview data from 25 young people (17-25 years) with eczema.[4,5,24] The data came from the SKINS project, which explored young people's experiences of living with common skin conditions (eczema, acne, psoriasis, alopecia). To gather additional views from younger adolescents, the ECO study added to this data set by carrying out interviews with five young people with eczema aged 13-16 years. Both interview studies explored young people's views about eczema treatment and management, and information and support needs. Interviews were analysed using inductive thematic analysis.

For peer review only - http://bmjopen.bmj.com/site/about/guidelines.xhtml

The methods and findings of these studies have been described in detail elsewhere.[4,5,7,24] Only key findings relevant to intervention development are summarised in this paper, with the focus of this paper being on intervention development and the novel findings from Phase 2.

1.3 Developing an intervention plan

Consistent with the person-based approach, our in-depth understanding of young people with eczema informed the development of *guiding principles*, which outlined key intervention objectives and design features that will address these.[19] A list of potential barriers and facilitators to the target behaviours were also identified from this evidence-base and from consultation with the multidisciplinary intervention development group and additional PPI representatives. A *behavioural analysis* outlined the intervention components that were added to address each of the identified barriers and facilitators for each target behaviour. Consistent with the approach taken by Band et al. and Greenwell et al.,[17,18] these components were mapped onto behaviour change theoretical frameworks to describe the planned intervention content and identify hypothesised mechanisms of action. The Behaviour Change Techniques Taxonomy classifies intervention content by their behaviour change techniques, the smallest component for changing behaviour.[25] The Behaviour Change Wheel was used to classify the source (component of the COM-B model hypothesised to influence behaviour; Capability, Opportunity, Motivation) and function (e.g. 'Education', 'Persuasion') of each individual or group of behaviour change techniques.[26] We also mapped the behaviour change techniques onto their theoretical constructs (e.g. 'Knowledge', 'Skills') using the Theoretical Domains Framework,[27] which is recommended for use alongside the Behaviour Change Wheel.

To illustrate key elements of the intervention's programme theory, a *logic model* was developed to illustrate how the intervention components, theoretical constructs (intervention

For peer review only - http://bmjopen.bmj.com/site/about/guidelines.xhtml

processes), and key behaviours (purported mediators) influence the intervention outcomes (eczema severity).

Phase 1 Results

1.1 Defining the intervention target behaviours

The multidisciplinary intervention development group agreed that ECO would aim to reduce eczema severity by supporting young people with eczema to: (1) Increase their use of emollients to maintain skin hydration and prevent flare-ups; (2) Improve their use of topical corticosteroids or topical calcineurin inhibitors through reactive applications of these treatments in response to flare-ups or, where appropriate, regular intermittent ("weekend") preventative applications of topical corticosteroids or topical calcineurin inhibitors if emollients are insufficient as maintenance therapy; (3) Improve their management of irritants and triggers; (4) Improve their emotional management; and (5) Reduce scratching. Use of emollients and topical corticosteroids/topical calcineurin inhibitors were identified as core behaviours that would likely have the greatest effect on eczema severity. Therefore, intervention content relating to these behaviours was deemed the most important.

1.2 Collating and synthesising evidence relating to the perspectives of young people with eczema

The qualitative evidence helped us to develop the following understanding of our target group. Young people are developing a sense of independent identity and have an increasing desire for autonomy. Specifically, young people with eczema are keen to take on more responsibility with their eczema management.[24] However, these young people may feel apprehensive about their new roles and responsibilities, with some finding interacting with health professionals and negotiating health care systems daunting.[24] Young people have gaps in their knowledge of eczema, specifically understanding of the causes/triggers of

For peer review only - http://bmjopen.bmj.com/site/about/guidelines.xhtml

eczema and the rationale behind their treatment (e.g. difference between emollients and topical corticosteroids/topical calcineurin inhibitors and how to use them).[4,7] Health professionals do not always revisit such information, assuming that young people were told this information in childhood.[4,7] In general, young people perceive topical treatments to be effective, but they also have doubts about their long-term effectiveness, and concerns around their safety and becoming over-reliant on topical corticosteroids.[7,24] This group perceive several barriers to applying topical treatments, including using treatments when outside of the home (e.g. when in class or working in a public-facing job) and cost of treatments.[7,24] These treatment barriers are not unique to this age group,[7] but are nonetheless important to address for any eczema behavioural intervention.

Many young people were told in childhood that they would 'grow out of' eczema, but this information is often at odds with their own experiences.[4] Young people have a desire to 'fit in' with their peers and feel like a 'normal' young person.[5] They welcome the opportunity to share experiences with other young people with eczema to normalise their experience.[5]

The multidisciplinary intervention development group discussed what specific website design needs young people may have. Usability research has shown that this group are likely to dislike reading large amounts of text, like concepts to be illustrated visually, and relate to content created by peers (e.g. stories, images, examples from other young people).[28,29] This evidence was supported by discussions with our young people PPI who also suggested that young people may prefer videos over reading lots of text and recommended having brief 'top tips' with suggestions from other young people for how they manage eczema. They also felt that it was important that the intervention was accessible via a mobile device or computer.

1.3 Developing an intervention plan

For peer review only - http://bmjopen.bmj.com/site/about/guidelines.xhtml

We developed a set of guiding principles to address the issues identified in the evidence synthesis stage (1.2) (Table 1). The behavioural analysis table is presented in Supplementary Material 1 and the logic model in Figure 2.

Phase 2: Intervention optimisation

Phase 2 Methods

2.1 Creating the intervention prototype and videos

Creating the intervention prototype was done in several stages. First, guided by our target behaviours, guiding principles and qualitative research, the multidisciplinary intervention development group agreed the topics of the intervention modules and videos. Second, we wrote page content and video scripts, and circulated this to the multidisciplinary intervention development group for comment to ensure it was evidence-based and medically accurate, and the advice was clear and feasible. Positive and negative feedback was entered into the Person-Based Approach Table of Changes,[16] and potential changes were discussed, agreed, and prioritised.

We tested either the video scripts, audio recordings of the scripts, or a storyboard or prototype of the video with young people using think-aloud interviews, and these were also reviewed by a PPI panel. Once the written content and videos were finalised, we created a working prototype of the intervention using the LifeGuide software,[30] which was reviewed by our two young people PPI and further optimised through think-aloud interviews with young people with eczema. The final videos were created by an external video creator.

For peer review only

Table 1 Guiding principles for ECO for young people

User context	Intervention design objectives	Key intervention features
• Young people (YP) with eczema have an increasing desire for autonomy regarding their eczema management, but may feel apprehensive about their new roles and responsibilities.<br>• YP may have gaps in their understanding of eczema.<br>• YP may perceive barriers to using topical treatments.	To support YP to gain autonomy and competence in their eczema management.	• Ensure YP have a complete understanding of eczema and the rationale behind their treatment.<br>• To build YP's self-efficacy for the target behaviours (e.g. information on how to apply treatments, avoid irritants/triggers, reduce scratching).<br>• Stories and tips from other YP on what helped them take control of their own eczema and how to overcome barriers to treatments.<br>• Use autonomy-promoting language, provide choice wherever possible, and avoid condescending or 'child-like' language/graphics.<br>• Provide advice on how to communicate with health professionals and make the most out of appointments.
• YP have a desire to live as 'normal' a life as possible.<br>• YPs may be told in childhood that they would 'grow out of' eczema, which is at odds with their own experiences.	To enable YP to maintain a sense of normalcy when managing their eczema	• Provide age-appropriate advice on living with eczema (e.g. shaving, wearing make-up, and managing eczema at work/university/school).<br>• Provide relatable stories and advice from other YP with eczema.<br>• Acknowledge that, for some, eczema persists into adolescence and adulthood.<br>• Provide images and descriptions of eczema for different skin-types.<br>• Avoid providing overly restrictive advice on irritants/triggers, instead offering advice on how to minimise the negative consequences of exposure irritants and triggers or provide alternatives (e.g. using emollients in place of soap).

For peer review only - http://bmjopen.bmj.com/site/about/guidelines.xhtml

• YPs may have doubts about the long-term effectiveness of topical treatments, and concerns around their safety and becoming over-reliant on topical corticosteroids<br>• YP may find topical treatments unpleasant in texture and/or smell and they worried about applying treatments in public in case others 'found out' that they had eczema.	To build YP's beliefs in the positive effects of their topical treatments	• Provide information to address topical treatment concerns and barriers, and persuade YPs of the long-term effectiveness of these treatments.
• YPs may dislike reading large amounts of text, preferring content that is easy to scan, visual and peer-created.<br>• YPs want interventions that are accessible on their smartphones and computers.	To provide engaging and accessible intervention content	• Provide interactive content (e.g. quizzes), videos and pictures, and reduce reading burden by keeping the amount of text per page to a minimum.<br>• Break the content down into lots of short sections/modules.<br>• Intervention to be mobile-friendly.<br>• Provide peer-created content (e.g. stories, videos)

For peer review only - http://bmjopen.bmj.com/site/about/guidelines.xhtml

2.2 Think-aloud interviews

We carried out 30 think-aloud interviews with 28 young people with eczema (2 people took part in two interviews, viewing a later intervention version) who were purposively sampled based on age, gender and eczema severity (Table 2). Twenty-three of these participants were newly recruited for this study and five participants were from the qualitative study in Phase 1. Participants were recruited via an invitation letter from their GP practice or advertising, opportunistic and snowball sampling of students at the University of Southampton. During the think-aloud interviews, participants were asked to use sections of the intervention while sharing their thoughts and reactions to the content aloud. Interviews were facilitated by a researcher who observed the participant using the intervention and asked prompts when needed to elicit participant reactions.

To maximise participant time during the interviews, participants testing optional modules were sent the core intervention content to look through before the interview and they were asked about it during the interview. Interviews were carried out at participants' preferred location (e.g. at home, the university) and relatives were present for nine interviews. Interviews lasted 45-90 minutes, took place between October 2018 to April 2019, and were carried out by DG and two medical students (HJ, EW) and one postdoctoral student were trained and supervised by DG and ET (postdoctoral experienced qualitative researchers) (all females). Interviews were audio-recorded and transcribed verbatim.

Optimisation was iterative, moving between data collection, analysis, and intervention modification. We considered data saturation to be reached once no further important changes were required.[16] For each interview, DG completed a feedback table, highlighting positive and negative comments about the intervention, based on the interviewer field notes and listening back to the audio recordings.

For peer review only - http://bmjopen.bmj.com/site/about/guidelines.xhtml

Table 2 Think-aloud participant demographics

Variable	Statistic
**Age**	
Mean (SD)	17.82 (3.41)
Range	13-23
**Gender**	
Female N (%)	13 (46.43)
Male N (%)	15 (53.57)
**Eczema severity (self-defined)***	
Mild N (%)	10 (38.46)
Mild/Moderate N (%)	2 (7.69)
Moderate N (%)	7 (26.92)
Moderate/Severe N (%)	3 (11.54)
Severe N (%)	4 (15.38)

Key: *n=26

MSa and IM read through the transcripts to ensure important issues were captured. The feedback table was reviewed by a sub-group of the intervention development group (KG, IM, MSa, KS, MSt, ET) at weekly meetings. KG transferred negative comments into the Table of Changes and potential changes were agreed and prioritised by this sub-group.[16]

Phase 2 Results

2.1 Creating the intervention prototype and videos

In the final intervention prototype, users first progressed through a brief (nine pages) introductory section containing the key behavioural messages necessary for facilitating a basic understanding of eczema and its management (Supplementary Material 2). Some key behavioural messages were also summarised in short (2 minute) videos (Supplementary Material 3). Users then had the option of completing a simple eczema assessment that provided advice on which of the core treatment modules (emollients or topical

For peer review only - http://bmjopen.bmj.com/site/about/guidelines.xhtml

corticosteroids) would be most relevant to them, depending on whether they were currently experiencing an eczema flare-up (Supplementary Material 4). Users had access to a menu where they could choose the topic modules that were most relevant to them (Supplementary Material 5). On each login, users were given suggestions for which modules to look at next, based on what they had looked at previously.

A key design feature highlighted in the guiding principles was the use of quotes from other young people with eczema sharing their experiences of eczema and eczema management advice (Supplementary Material 4). We also introduced new terminology to help young people better understand the function of each type of topical treatment and to mirror how this group already referred to these treatments (e.g. ‘creams’ for all treatments, regardless of whether they were actually a cream or an ointment). Emollients were termed ‘moisturising creams’ and topical corticosteroids/topical calcineurin inhibitors were termed ‘flare control creams’.

Our multidisciplinary intervention development group and PPI representatives felt that it was important that the intervention is accessible and relevant to all ethnic groups. Therefore, ECO provided images and descriptions of eczema for different skin-types and our videos included cartoon characters from different ethnic groups. The full intervention content is outlined in Supplementary Material 6 using the TIDieR checklist.[31]

2.2 Think-aloud interviews

Generally, young people expressed positive views of the intervention’s content and design. They found the information and advice clear, easy to follow, helpful, and relatable, and they liked the videos and brief eczema assessment. Specifically, they found the quotes and advice from other young people with eczema, and the facts about how common it is to still have symptoms at their age reassuring, personal, and it made them feel less alone.

For peer review only - http://bmjopen.bmj.com/site/about/guidelines.xhtml

> *"I think it's quite comforting to actually know that it's normal for people with eczema. As in like this is, it's not just me, because even though you get told loads of people have eczema, it is just quite nice to be like, 'This is what you're going through, it's okay, this is what you're going to do about it,' and just have the information all there." (P10, 19-21 years old)*

> *"I like this page [content on prevalence of eczema among young people]...it's good to know that, like, I don't know, I always feel quite weird, because I'm 21 and still have eczema, so, it's good to know that." (P17, 19-21 years old)*

Young people valued that ECO provided advice on living well with eczema, rather than focusing solely on medical treatments.

> *"Sleep problem, emotion, diet and how much you eat - that's good because it's saying that it's not just eczema like using creams, it's also got other stuff, other aspects to it as well...I think you can't just control eczema with creams, you've got to control the whole lot. Yes, this is useful. (P13, 19-21 years old)*

> *"In lighter skin eczema may look red, in darker skin eczema may look grey, purple or brown'. I feel like saying that is actually good, because a lot of the time I'm just like, why does my eczema look grey? But I didn't even know that, now I can see that's a common thing in darker skin. I feel like having that is actually really good. (P22, 16-18 years old)*

> *"I think that [module on itching] was the most helpful because it said apart from using the creams and things, other things you can do to help you. (P19, 13-15 years old)*

For peer review only - http://bmjopen.bmj.com/site/about/guidelines.xhtml

Most young people explained how they learned something new about eczema and its management from the part of the website they used, with some explaining that ECO helped addressed the knowledge gaps from childhood.

> *"[ECO is] brilliant. It's given me a lot more information than I've ever had in the past…I've learnt a lot of new things today about eczema that I didn't know over the last 22 years of having eczema, it's amazing!...It's amazing, I love it." (P9, 22-25 years old)*

> *"If I was prescribed stuff by my doctor I would be like, 'Well, it's probably good for me.'...I'd be like why am I even using this and then not bother. I think if you knew that, okay this one's going to stop you itching and reduce soreness then you might be more likely to carry on using it and also, yes if you're someone that doesn't really ask questions it's nice to have a place that will tell you the information anyway without you having to ask someone. (P18, 16-18 years old)*

Young people also talked about how they found the information about the safety of topical corticosteroids reassuring:

> *"That's good to know, that it [topical corticosteroids] doesn't affect growth or development…because I've been using it for so many years now. It's always been on my mind, and not being able to find out any information about it, it's worried me…but this helps a lot. It kind of puts your mind at ease, knowing that it doesn't do anything to your growth or development." (P9, 22-25 years old)*

The positive feedback provided support for our guiding principles, which emphasised the importance of maintaining a sense of normalcy when managing eczema, addressing knowledge gaps to support young people to gain autonomy and competence in eczema management, and providing engaging and accessible intervention content (Table 1). The

For peer review only - http://bmjopen.bmj.com/site/about/guidelines.xhtml

negative feedback was used to modify the intervention (Table 3 outlines some example negative comments and the changes implemented).

Discussion

This manuscript provides a description of the rigorous development process for a behavioural intervention for young people with eczema; a target group that has been largely ignored in eczema research.[7,9,10] It is essential to provide self-management support to this age group as they begin to take on a more active role in their eczema management; a role which was previously taken on by their families.[6] The person-based approach to intervention development allowed us to understand and accommodate the perspectives of young people with eczema, resulting in an online intervention that was engaging and acceptable to our sample of this target group.[19]

The behavioural analysis maintained a focus on the behaviours most likely to influence eczema severity (topical treatment use, managing irritants/triggers, scratching, emotional management) and suggested acceptable behaviour change techniques that can help young people with eczema overcome behavioural barriers relevant to them. Logic modelling offered a programme theory that can be tested and refined in future process analyses. Our final guiding principles outlined some of the key behavioural issues, intervention design objectives, and design features that those developing behavioural interventions for young people with eczema, or other long-term conditions, may wish to consider to maximise user engagement. The think-aloud interviews provided support for the relevance of these guiding principles to this target group and the acceptability of the design features we implemented to engage this group. Specifically, the ECO intervention's use of quotes and tips from other young people with eczema and its focus on living with eczema (not just topical treatments) were valuable for normalising eczema.

For peer review only - http://bmjopen.bmj.com/site/about/guidelines.xhtml

Table 3 Example issues identified from the think-aloud interviews and the changes implemented to address these

**Summary of issue identified**	**Example quote**	**Change implemented**
Some found the questions in the brief eczema assessment confusing.	*"'Does your skin feel dry?' Well, everywhere or in general where you've got…eczema? …I don't know if that's [the question] very clear." (P12, 22-25 year old)*<br><br>*"'Is your skin or redder or darker than usual?'…to me it sounds like 'is your eczema darker or less dark than your eczema normally is?', but I think it's a bit ambiguous whether it means that or their usual skin?...you could just say 'is your eczema red or darker than usual?'" (P18, 16-18 years old)*<br><br>*'Is your skin redder or darker [than usual]?'...maybe it's the 'than usual' bit that needs to be more clearer, because I could interpret that as this being my usual skin, rather than the usual colour of the eczema…maybe, 'Is your eczema redder or darker than usual?' or something (P5, 19-21 years old)*	The assessment questions were reworded to clarify that we are asking about their eczema at present (rather than in the past) and their eczema skin (rather than skin in general).
The feedback for the brief eczema assessment did not always match their experience (e.g. feedback suggested they may have an eczema flare-up when they didn't).	*"[Is your skin itchy or sore?] it's not really sore but it is itchy…I can have itchy dry skin but I only have sore skin when it's red and inflamed…[recommended the flare control creams module]… it said that I'm having a flare-up now, but I wouldn't class this as a flare-up. This is kind of just*	We separately asked whether eczema is 'itchy' and 'sore' so that people do not receive feedback that they are having a flare-up if they just have itchy and not sore skin. Soften the feedback on current eczema severity to avoid disengaging those who don't agree that they are

For peer review only - http://bmjopen.bmj.com/site/about/guidelines.xhtml

Summary of issue identified	Example quote	Change implemented
	my - how my eczema kind of bobbles along, as in my flare-ups would be much more aggressive than what they were suggesting it is." (P10, 19-21 years old)	experiencing an eczema flare-up ('For most people, this means they are having an eczema flare-up').
Some thought the introductory section was too long.	*"Personally, if I see there's 21 pages, I'm just going to try and get through them." (P18, 16-18 years old)*	Reduce the introductory section significantly with optional click-outs to additional information if needed.
Some young people commented that they already knew a lot of the information as they have had eczema for a long time.	*"[ECO] was really good…because I've had it for so long I feel like a lot of the information there I've already had drilled into me my whole life, but I feel like for people that either have children with eczema or people with eczema, I don't know, I think it would be really helpful... personally, I would probably want the more information about the lifestyle stuff and diet" (P10, 19-21 years old)*<br><br>*"I think a lot of stuff I probably kind of already knew from having eczema but it's good to get a few things that help yes" (P19, 13-15 years old)*	Added in a quote from another young person who had eczema for a long time saying how they were surprised that the website contained new helpful tips. Emphasise that the information is based on the most up-to-date research evidence (so they may find new information) and that there is information on 'living with eczema' (e.g. diet advice).

The value of behaviour change interventions that make individuals feel they are 'not alone' is supported by other qualitative research with people with long-term conditions and parents/carers of children with eczema.[22,32] Although this intervention focused mainly on treatment adherence as this was deemed to have the biggest influence on eczema severity, providing age-appropriate advice and support on how young people can better live with

For peer review only - http://bmjopen.bmj.com/site/about/guidelines.xhtml

eczema was valuable for ensuring the content was engaging and relatable to this target group. Young people explained how valuable the explanations of eczema and rationale behind the topical treatments for addressing gaps in their knowledge, with most participants reporting that they learned something new from ECO. This provided further support for the need for health services to revisit eczema education with young people, avoiding assumptions that they have already been told this information.[4] Online information can be confusing and of variable quality,[33,34] therefore, it is important to signpost young people to high quality evidence-based online information so they are empowered to take an active role in their own healthcare.[35]

A strength of this research was our rigorous development using complementary theory-, evidence-, and person-based approaches to ensure the intervention is acceptable and engaging to its target group, as well as effective. Our multidisciplinary intervention development group and PPI ensured that the content was evidence-based, that advice was feasible, and that the perspectives of people living with eczema were considered throughout the whole development process.[36] It was helpful to gain iterative feedback from PPI and young people with eczema on early versions of the videos (i.e. scripts, audio recordings of the video voiceover, prototype) to ensure that they were as acceptable as possible to the target group before it was finalised by the external video creator. One limitation of the think-aloud interview study is that a large proportion of the sample were university students, so are likely to have high levels of health and computer literacy. Furthermore, as think-aloud interviews explore participants' immediate reactions to the intervention, they cannot tell us how people would use the intervention over time or explore engagement with the target behaviours.

In conclusion, the think-aloud interview study demonstrated ECO is likely to be acceptable to young people with eczema and provided support for our guiding principles, including key design objectives and features to consider when developing interventions for this population.

For peer review only - http://bmjopen.bmj.com/site/about/guidelines.xhtml

A randomised controlled trial of ECO to explore effectiveness and cost-effectiveness is underway (ISRCTN79282252).[13] This includes a nested mixed-methods process evaluation to explore young people's experiences of using the intervention over time and how the intervention was used, and test and further refine the intervention's programme theory. For the qualitative interview study, we purposively recruited participants across a range of ages, ethnicities, socioeconomic status, eczema severities, and intervention usage. It is important to recruit diverse samples to ensure digital interventions do not further facilitate healthcare inequalities.

Acknowledgments

We would like to thank our young people PPI Taheeya and Tahmid Ahmed and the panel of public contributors for their valuable feedback on the Eczema Care Online intervention.

Funding

This article presents independent research funded by the National Institute for Health Research (NIHR) under its Programme Grants for Applied Research (PGfAR) Programme (Grant Reference Number RP-PG-0216-20007). Eczema Care Online (ECO) was developed using the LifeGuide software, which was partly funded by the NIHR Biomedical Research Centre (BRC), Southampton. The views expressed are those of the authors and not necessarily those of the NIHR or the Department of Health and Social Care. SML is funded by a Wellcome Senior Clinical Research fellowship [205039/Z/16/Z]. This research was funded in whole or in part by the Wellcome Trust [205039/Z/16/Z]. For the purpose of Open Access, the author has applied a CC BY public copyright licence to any Author Accepted Manuscript (AAM) version arising from this submission.

For peer review only - http://bmjopen.bmj.com/site/about/guidelines.xhtml

Competing interests

SML is a co-investigator on the IMI Horizon 2020 project BIOMAP, but is not in receipt of industry funding. The other authors have no competing interests to declare.

Author Contributions

All authors (except HJ, EW, ELR) designed the study. KG, KS, DG, MSt IM, MSa led the intervention development with input from the other authors. DG, ET, SW, HJ, EW were responsible for recruitment and data collection. KG and DG led on the data analysis, with support from MSa and IM. KG drafted the manuscript, with initial support from DG, MSa and IM. All authors critically reviewed the manuscript, contributing important intellectual content and approved the final manuscript.

Data sharing statement

The data that support the findings of this study are available from the corresponding author upon reasonable request.

References

1 Bylund S, Von Kobyletzki LB, Svalstedt M, *et al.* Prevalence and incidence of atopic dermatitis: A systematic review. *Acta Derm Venereol* 2020;**100**:320–9. doi:10.2340/00015555-3510

2 Margolis JS, Abuabara K, Bilker W, *et al.* Persistence of mild to moderate atopic dermatitis. *JAMA Dermatology* 2014;**150**:593–600. doi:10.1001/jamadermatol.2013.10271

3 National Institute for Health and Care Excellence (NICE). Atopic eczema in under 12s: diagnosis and management (Clinical guideline 57). 2007. https://www.nice.org.uk/guidance/cg57 (accessed 27 Aug 2021).

For peer review only - http://bmjopen.bmj.com/site/about/guidelines.xhtml

4 Ghio D, Muller I, Greenwell K, *et al.* 'It's like the bad guy in a movie who just doesn't die': A qualitative exploration of young people's adaptation to eczema and implications for self-care. *Br J Dermatol* 2020;**182**:112–8. doi:10.1111/bjd.18046

5 Ghio D, Greenwell K, Muller I, *et al.* Psychosocial needs of adolescents and young adults with eczema: A secondary analysis of qualitative data to inform a behaviour change intervention. *Br J Health Psychol* 2020;**26**:214–23. doi:10.1111/bjhp.12467

6 Kosse RC, Bouvy ML, Daanen M, *et al.* Adolescents' perspectives on atopic dermatitis treatment-experiences, preferences, and beliefs. *JAMA Dermatology* 2018;**154**:824–7. doi:10.1001/jamadermatol.2018.1096

7 Teasdale E, Muller I, Sivyer K, *et al.* Views and experiences of managing eczema: systematic review and thematic synthesis of qualitative studies. *Br J Dermatol* 2021;**184**:627–37. doi:10.1111/bjd.19299

8 De Vere Hunt IJ, McNiven A, Roberts A, *et al.* 'Not just a piece of skin in front of you' - A qualitative exploration of the experiences of adolescents with eczema and psoriasis with healthcare professionals. *BMJ Open* 2021;**11**. doi:10.1136/bmjopen-2020-041108

9 Ridd MJ, King AJL, Le Roux E, *et al.* Systematic review of self-management interventions for people with eczema. *Br J Dermatol* 2017;**177**:719–34. doi:10.1111/bjd.15601

10 Pickett K, Frampton G, Loveman E. Education to improve quality of life of people with chronic inflammatory skin conditions: a systematic review of the evidence. *Br J Dermatol* 2016;**174**:1228–41. doi:10.1111/bjd.14435

11 Staab D, Diepgen TL, Fartasch M, *et al.* Age related, structured educational

For peer review only - http://bmjopen.bmj.com/site/about/guidelines.xhtml

programmes for the management of atopic dermatitis in children and adolescents: Multicentre, randomised controlled trial. *Br Med J* 2006;**332**:933–6. doi:10.1136/bmj.332.7547.933

12 Moore EJ, Williams A, Manias E, *et al.* Eczema workshops reduce severity of childhood atopic eczema. *Australas J Dermatol* 2009;**50**:100–6. doi:10.1111/j.1440-0960.2009.00515.x

13 Muller I, Stuart B, Sach T, *et al.* Supporting self-care for eczema: Protocol for two randomised controlled trials of ECO (Eczema Care Online) interventions for young people and parents/carers. *BMJ Open* 2021;**11**. doi:10.1136/bmjopen-2020-045583

14 Sivyer K, Teasdale E, Greenwell K, *et al.* Supporting parents and carers to manage and co-manage their child's eczema: Intervention planning and optimisation for Eczema Care Online for Families. In review.

15 O'Cathain A, Croot L, Duncan E, *et al.* Guidance on how to develop complex interventions to improve health and healthcare. *BMJ Open* 2019;**9**:e029954. doi:10.1136/bmjopen-2019-029954

16 Bradbury K, Morton K, Band R, *et al.* Using the person-based approach to optimise a digital intervention for the management of hypertension. *PLoS One* 2018;**13**:1–18. doi:10.1371/journal.pone.0196868

17 Band R, Bradbury K, Morton K, *et al.* Intervention planning for a digital intervention for self-management of hypertension: a theory-, evidence- and person-based approach. *Implement Sci* 2017;**12**:25. doi:10.1186/s13012-017-0553-4

18 Greenwell K, Sivyer K, Vedhara K, *et al.* Intervention planning for the REDUCE maintenance intervention: a digital intervention to reduce reulceration risk among

For peer review only - http://bmjopen.bmj.com/site/about/guidelines.xhtml

patients with a history of diabetic foot ulcers. *BMJ Open* 2018;**8**:e019865. doi:10.1136/bmjopen-2017-019865

19 Yardley L, Morrison L, Bradbury K, *et al.* The person-based approach to intervention development: Application to digital health-related behavior change interventions. *J Med Internet Res* 2015;**17**:e30. doi:10.2196/jmir.4055

20 Moore G, Audrey S, Barker M, *et al.* Process evaluation of complex interventions: UK medical research council (MRC) guidance. MRC 2014. https://www.mrc.ac.uk/documents/pdf/mrc-phsrn-process-evaluation-guidance-final/ (accessed 27 Aug 2021).

21 Yardley L, Ainsworth B, Arden-Close E, *et al.* The person-based approach to enhancing the acceptability and feasibility of interventions. *Pilot Feasibility Stud* 2015;**1**:37. doi:10.1186/s40814-015-0033-z

22 Santer M, Muller I, Yardley L, *et al.* Supporting self-care for families of children with eczema with a web-based intervention plus health care professional support: Pilot randomized controlled trial. *J Med Internet Res* 2014;**16**:1–27. doi:10.2196/jmir.3035

23 van Zuuren EJ, Fedorowicz Z, Christensen R, *et al.* Emollients and moisturisers for eczema. *Cochrane Database Syst Rev* 2017; Issue 2. Art. No.: CD012119. doi:10.1002/14651858.CD012119.pub2

24 Greenwell K, Ghio D, Muller I, *et al.* Taking charge of eczema self-management: a qualitative interview study with young people with eczema. *BMJ Open* 2021;**11**:e044005. doi:10.1136/bmjopen-2020-044005

25 Michie S, Richardson M, Johnston M, *et al.* The Behavior Change Technique Taxonomy (v1) of 93 Hierarchically Clustered Techniques: Building an International

For peer review only - http://bmjopen.bmj.com/site/about/guidelines.xhtml

Consensus for the Reporting of Behavior Change Interventions. *Ann Behav Med* 2013;**46**:81–95. doi:10.1007/s12160-013-9486-6

26 Michie S, Atkins L, West R. *The Behaviour Change Wheel: A Guide to Designing Interventions*. London: Silverback Publishing 2014.

27 Cane J, O'Connor D, Michie S. Validation of the theoretical domains framework for use in behaviour change and implementation research. *Implement Sci* 2012;**7**:37. doi:10.1186/1748-5908-7-37

28 Mccloskey BM, Loranger H, Nielsen J. *Teenagers (Ages 13-17) on the Web*. Nielsen Norman Group 2013.

29 Nielsen J. *Designing for Young Adults (Ages 18-25)*. Nielsen Norman Group 2016.

30 Williams S, Yardley L, Weal M. Introduction to LifeGuide: opensource software for creating online interventions for health care, health promotion and training. *Knowl Resour* 2010;**187**:90.

31 Hoffmann TC, Glasziou PP, Boutron I, *et al.* Better reporting of interventions: template for intervention description and replication (TIDieR) checklist and guide. *BMJ* 2014;**348**:g1687. doi:10.1136/bmj.g1687

32 Greenwell K, Sereda M, Coulson NS, *et al.* 'That's just how I am': a qualitative interview study to identify factors influencing engagement with a digital intervention for tinnitus self-management. *Br J Health Psychol* 2020;:bjhp.12486. doi:10.1111/bjhp.12486

33 Halls A, Nunes D, Muller I, *et al.* 'Hope you find your "eureka" moment soon': A qualitative study of parents/carers' online discussions around allergy, allergy tests and eczema. *BMJ Open* 2018;**8**. doi:10.1136/bmjopen-2018-022861

For peer review only - http://bmjopen.bmj.com/site/about/guidelines.xhtml

34 Santer M, Chandler D, Lown M, *et al.* Views of oral antibiotics and advice seeking about acne: a qualitative study of online discussion forums. *Br J Dermatol* 2017;**177**:751–7. doi:10.1111/bjd.15398

35 National Institute for Health and Care Excellence (NICE). Babies, children and young people's experience of healthcare [NG204]. 2021. https://www.nice.org.uk/guidance/indevelopment/gid-ng10119 (accessed 27 Aug 2021).

36 Muller I, Santer M, Morrison L, *et al.* Combining qualitative research with PPI: reflections on using the person-based approach for developing behavioural interventions. *Res Involv Engagem* 2019;**5**:1–8. doi:10.1186/s40900-019-0169-8

Figure legends

Figure 1 Intervention development process for ECO intervention. ***Key:*** *PBA = Person-Based Approach; EBA = Evidence-based approach; TBA = Theory-based approach.*

Figure 2 Logic model for Eczema Care Online (ECO) for young people

For peer review only - http://bmjopen.bmj.com/site/about/guidelines.xhtml
For peer review only

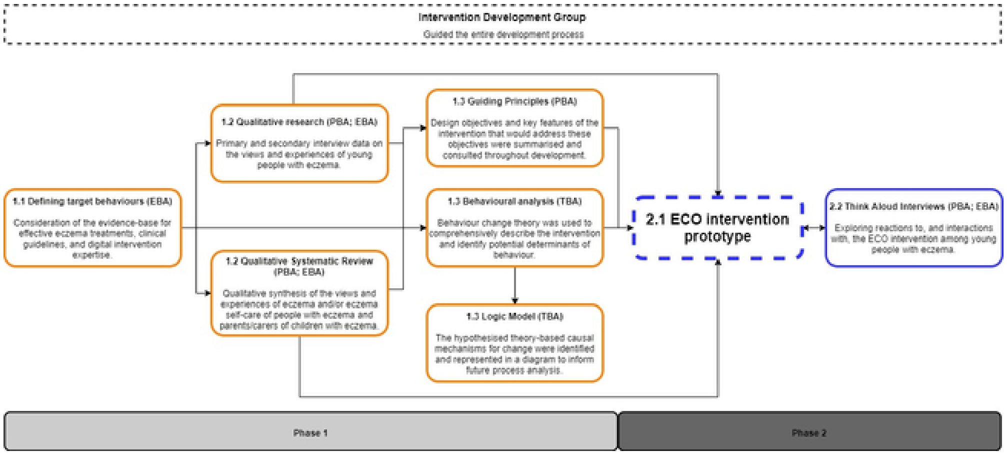

Intervention development process for ECO intervention. Key: PBA = Person-Based Approach; EBA = Evidence-based approach; TBA = Theory-based approach.

55x24mm (300 x 300 DPI)

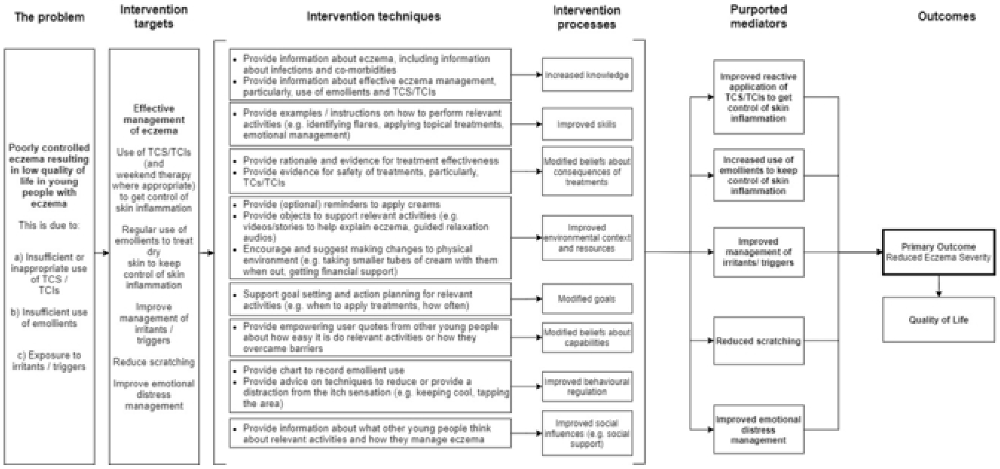

Logic model for Eczema Care Online (ECO) for young people

56x26mm (300 x 300 DPI)

Supplementary Material 1: Behavioural analysis for the Eczema Care Online (ECO) intervention for young people using the Behaviour Change Wheel (BCW), Theoretical Domains Framework (TDF) and Behaviour Change Techniques Taxonomy (BCTv1)

Key: TCS = Topical Corticosteroids; YP = Young People; EO = Barrier emerged from expert opinion; QSR = Barrier/facilitator emerged from systematic review of qualitative literature with adults with eczema and parents/carers of children with eczema; QI = Barrier/facilitator emerged from qualitative interview research with young people.

**Barriers/*facilitator* to target behaviour**	**Intervention components**	**Target construct (BCW)**	**Theoretical domain (TDF)**	**Intervention function (BCW)**	**Behaviour Change Technique (using BCTv1)**
**Target behaviour: Increased emollient use**					
Belief that emollients (in general or a specific brand) do little to control eczema [QSR; QI]<br><br>*Belief that emollients are useful for preventing dry/cracking skin [QI]*	• Provide persuasive and credible information about the effectiveness of emollients, including scientific evidence, user quotes, and videos<br>• Provide rationale for how emollients control eczema	Psychological capability; Reflective motivation; Social opportunity	Beliefs about consequences; Knowledge; Social Influences	Education; Persuasion; Modelling	5.1 Information about health consequences<br>6.2 Social comparison<br>6.3 Information about others' approval<br>9.1 Credible source
	The 2-week challenge:<br>• Provide an emollient chart to allow YP to record how their skin is after applying emollients	Reflective Motivation	Beliefs about consequences	Education; Persuasion	5.1 Information about health consequences<br>2.4 Self-monitoring of outcomes of behaviour
	• Provide advice on how to choose an effective emollient (e.g. list of available emollients, disadvantages of using cosmetic moisturisers)	Psychological capability	Skills	Training	4.1 Instructions on how to perform the behaviour
Concerns about the safety and side effects (e.g. stinging, dependency) of emollients smell, feel, and appearance of emollients [QSR; QI QI]	• Provide persuasive and credible information about the safety of emollients and risk of side effects (including their flammability), including scientific evidence, user quotes and videos	Psychological capability; Reflective motivation; Social opportunity	Beliefs about consequences; Knowledge; Social Influences	Education; Persuasion; Modelling	5.1 Information about health consequences<br>6.2 Social comparison<br>6.3 Information about others' approval<br>9.1 Credible source
	• Provide advice on how to choose the right emollient (e.g. using different emollients at different times of the day, highlighting that different	Psychological capability	Skills	Training	4.1 Instructions on how to perform the behaviour

For peer review only - http://bmjopen.bmj.com/site/about/guidelines.xhtml

**Barriers/*facilitator* to target behaviour**	**Intervention components**	**Target construct (BCW)**	**Theoretical domain (TDF)**	**Intervention function (BCW)**	**Behaviour Change Technique (using BCTv1)**
	emollients have different constituents, smells, and feel)<br>• Provide advice on disguising emollients when around others/away from home (e.g. putting emollient in smaller containers)				
	The 2-week challenge:<br>• Provide an emollient chart to allow YP to record how their skin is each day after applying emollients and how they have found the emollients (e.g. side effects, texture)	Reflective Motivation	Beliefs about consequences	Education; Persuasion	5.1 Information about health consequences<br>2.4 Self-monitoring of outcomes of behaviour
Concerns about the psychosocial impact of emollients (e.g. feeling self-conscious) [QSR; QI]	• Provide strategies to reduce the psychosocial impact of emollients (e.g. feeling less self-conscious)<br>• Provide quotes from YPs explaining how they overcame some of the psychosocial consequences of emollients	Psychological capability; Reflective Motivation; Social opportunity	Beliefs about consequences; Beliefs about capabilities; Knowledge; Skills; Social influences	Education; Persuasion; Training; Modelling	5.3 Information about social and environmental consequences<br>5.6 Information about emotional consequences<br>6.2 Social comparison<br>6.3 Information about others' approval<br>9.1 Credible source
Inconvenience of using emollients when away from home [QSR; QI]	• Provide advice on using emollients when away from home (e.g. requesting smaller tubes from health professional)	Physical Opportunity	Environmental context and resources	Environmental restructuring	12.1 Restructuring the physical environment
Inconvenience of emollients rubbing off (e.g. on clothes and bed sheets) [QSR; QI]	• Provide advice on how to choose the right emollient and avoiding them rubbing off (e.g. put on loose clothing, allowing adequate time for absorption, leaving it to dry in a warm room)	Psychological capability	Skills	Training	4.1 Instructions on how to perform the behaviour

For peer review only - http://bmjopen.bmj.com/site/about/guidelines.xhtml

**Barriers/*facilitator* to target behaviour**	**Intervention components**	**Target construct (BCW)**	**Theoretical domain (TDF)**	**Intervention function (BCW)**	**Behaviour Change Technique (using BCTv1)**
Belief that emollients are time-consuming to apply and to find the right one/competing time pressures [QSR; QI]<br><br>Forgetting to apply emollients [QI]<br><br>*Having an emollient routine [QI]*	• Provide information on how to integrate emollient use into everyday life<br>• Reassure YP that applying emollients should not be time-consuming<br>• Advise YP to plan when they will apply their emollients (i.e. ensure they allocate time)	Psychological capability; Reflective Motivation	Knowledge; Skills; Goals	Education; Training; Enablement	1.4 Action planning<br>4.1 Instructions on how to perform the behaviour<br>5.3 Information about social and environmental consequences
	The 2-week challenge:<br>• Suggest YP to apply their emollient daily<br>• Suggest YP plan when they will apply their emollients<br>• Suggest YP apply their creams at the same time in the same context each day<br>• Allow YP to choose how many times per day they aim to apply their emollients and suggest they choose a particular time of the day or situation (e.g. after showering) to apply.<br>• Provide an emollient chart to record whether they have used their emollients at their agreed times.	Reflective motivation; Psychological capability	Goals; Skills; Behavioural regulation	Enablement; Training	1.1 Goal setting (behaviour)<br>1.4 Action planning<br>2.3 Self-monitoring of behaviour<br>8.1 Behavioural practice/rehearsal<br>8.3 Habit formation
	• Allow users to set up regular reminders to apply emollients by email or text messages and decide on the frequency of these reminders<br>• Provide an emollient chart that acts as a reminder by suggesting YP record whether they have used their	Physical opportunity	Environmental context and resources	Environmental structuring	7.1 Prompts/cues

**Barriers/*facilitator* to target behaviour**	**Intervention components**	**Target construct (BCW)**	**Theoretical domain (TDF)**	**Intervention function (BCW)**	**Behaviour Change Technique (using BCTv1)**
	emollients at their agreed times [2-week challenge]				
Belief that finding an emollient that works best for you is inconvenient, confusing and time-consuming [QSR; QI]	• Acknowledge how frustrating and time-consuming this process can be<br>• Provide quotes from YP emphasising the importance of finding the right emollient	Psychological capability; Reflective motivation; Social opportunity	Beliefs about consequences; Beliefs about capabilities; Knowledge; Social Influences	Education; Persuasion; Modelling	5.1 Information about health consequences<br>6.2 Social comparison<br>6.3 Information about others' approval<br>9.1 Credible source
Belief that you don't need to apply the emollient every day or only when you need it/eczema is bad [QI]	• Provide information on how often to apply emollients and the rationale for doing this	Psychological capability; Reflective Motivation	Knowledge; Skills	Education; Training	4.1 Instructions on how to perform the behaviour<br>5.1 Information about health consequences
Lack of skills regarding how to apply emollients/ low self-efficacy [QSR]	• Provide instructions on how to correctly applying emollients, including how much emollient to apply	Psychological capability	Skills	Training	4.1 Instructions on how to perform the behaviour
Ran out of emollients [EO]<br><br>*Stocking up on emollient [QI]*	• Provide advice on how to obtain more emollients/avoid running out	Physical Opportunity	Environmental context and resources	Environmental structuring	12.5 Adding objects to the environment
Cost of emollients [QI]	• Provide advice about financial benefits YP can apply for or strategies for reducing the cost of emollients	Physical Opportunity; Psychological Capability; Reflective Motivation	Environmental context and resources; Knowledge; Beliefs about consequences	Environmental structuring; Education; Persuasion	5.3 Information about social and environmental consequences<br>12.5 Adding objects to the environment
Difficulties in getting health professionals to	• Provide advice on how to prepare for appointments with health professionals	Psychological Capability	Skills	Training	4.1 Instructions on how to perform the behaviour

For peer review only - http://bmjopen.bmj.com/site/about/guidelines.xhtml

**Barriers/*facilitator* to target behaviour**	**Intervention components**	**Target construct (BCW)**	**Theoretical domain (TDF)**	**Intervention function (BCW)**	**Behaviour Change Technique (using BCTv1)**
prescribe different emollients [QI]<br><br>*Preparing and researching for consultations [QI]*					
**Target behaviour: Improved use of topical corticosteroids (TCS) or Topical Calcineurin Inhibitors (TCI)**					
Belief that TCSs/TCIs are not effective enough for managing flare-ups (e.g. provide only temporary relief before their eczema returned) [QI]	• Provide persuasive and credible information about the effectiveness of TCS/TCIs, including scientific evidence, user quotes and videos<br>• Provide rationale for how TCS/TCIs control eczema<br>• Provide advice on whether emollients are also needed	Psychological capability; Reflective motivation; Social opportunity	Beliefs about consequences; Knowledge; Social Influences	Education; Persuasion; Modelling	5.1 Information about health consequences<br>6.2 Social comparison<br>6.3 Information about others' approval<br>9.1 Credible source
	• Provide advice on how to choose an effective TCS/TCIs	Psychological capability	Skills	Training	4.1 Instructions on how to perform the behaviour
Concerns about the long-term safety of TCSs [QSR; QI; EO]	• Provide persuasive and credible information about the safety of TCSs/TCIs (e.g. skin thinning, wrinkling, dependency), including scientific evidence, user quotes and videos	Psychological capability; Reflective motivation; Social opportunity	Beliefs about consequences; Knowledge; Social Influences	Education; Persuasion; Modelling	5.1 Information about health consequences<br>6.2 Social comparison<br>6.3 Information about others' approval<br>9.1 Credible source
Concerns about the immediate side effects (e.g. stinging) of TCSs/TCIs [QI]	• Provide reassuring advice about the temporary nature of side effects (e.g. stinging) and how to choose the right TCS/TCIs (i.e. no side effects)	Psychological capability; Reflective motivation;	Skills; Beliefs about consequences; Knowledge	Education; Persuasion; Training	4.1 Instructions on how to perform the behaviour<br>5.1 Information about health consequences
Uncertainty regarding when to start and finish TCSs/TCIs [QI]	• Provide information on when to apply TCSs/TCIs and for how long	Psychological capability	Skills	Training	4.1 Instructions on how to perform the behaviour

For peer review only - http://bmjopen.bmj.com/site/about/guidelines.xhtml

**Barriers/*facilitator* to target behaviour**	**Intervention components**	**Target construct (BCW)**	**Theoretical domain (TDF)**	**Intervention function (BCW)**	**Behaviour Change Technique (using BCTv1)**
Lack of skills regarding how to apply TCSs/TCIs / low self-efficacy [EO]	• Provide instructions on how to correctly apply TCSs/TCIs, including how much to apply	Psychological capability	Skills	Training	4.1 Instructions on how to perform the behaviour
Belief that using more TCS than prescribed will conceal their eczema or make their eczema flare-up pass quicker [QI]	• Provide information on how much TCS/TCIs to apply	Psychological capability	Skills	Training	4.1 Instructions on how to perform the behaviour
Uncertainty regarding the difference between steroids [QI]	• Provide information on what type of steroids are available and which ones they should use on different body parts and why	Psychological capability	Skills	Training	4.1 Instructions on how to perform the behaviour
Ran out of TCS/TCIs /not being able to get hold of their preferred TCS (e.g. out of stock) [QI]<br><br>*Stocking up on TCS/TCIs [QI]*	• Provide information on how to obtain more TCS/TCIs and avoid running out	Physical Opportunity	Environmental context and resources	Environmental structuring	12.5 Adding objects to the environment
Belief that health professionals (including pharmacists) are reluctant or hesitant to prescribe TCSs/the right potency [QI]	• Provide advice on how to talk to health professionals<br>• Provide advice on how to choose an effective TC/TCIs S (e.g. list of available TCSs/TCIs)	Psychological capability	Skills	Training	4.1 Instructions on how to perform the behaviour

For peer review only - http://bmjopen.bmj.com/site/about/guidelines.xhtml

**Barriers/*facilitator* to target behaviour**	**Intervention components**	**Target construct (BCW)**	**Theoretical domain (TDF)**	**Intervention function (BCW)**	**Behaviour Change Technique (using BCTv1)**
	• Provide explanation of how the health professional decides on the TCS/TCI prescription				
Cost of TCSs/TCIs [QI]	• Provide advice about financial benefits YP can apply for or strategies for reducing the cost of TCSs/TCIs	Psychological capability; Reflective motivation	Beliefs about consequences; Knowledge	Education; Persuasion	5.3 Information about social and environmental consequences
**Target behaviour: Management of irritants and triggers**					
Lack of knowledge regarding common irritants and triggers [EO]	• Provide information on common irritants and triggers<br>• Provide information on misconceptions (e.g. food allergies)	Psychological capability; Reflective Motivation	Beliefs about consequences; Knowledge; Skills	Education; Persuasion; Training	4.1 Instructions on how to perform the behaviour<br>5.1 Information about health consequences
Belief that you can't avoid some triggers (e.g. stress)/Belief that the benefits of avoidance (e.g. avoiding a brief flare-up) do not outweigh the costs (e.g. not being able to go swimming) [QI]	• Where appropriate, provide advice on how to minimise the effects of irritants/triggers (e.g. apply emollients before and/or afterwards)<br>• Where appropriate, provide advice on how to avoid or reduce contact with certain irritants/triggers (e.g. soaps, high temperatures, sweat), including when away from home	Psychological capability; Reflective Motivation	Beliefs about consequences; Knowledge; Skills	Education; Persuasion; Training	4.1 Instructions on how to perform the behaviour<br>5.1 Information about health consequences

For peer review only - http://bmjopen.bmj.com/site/about/guidelines.xhtml

**Barriers/*facilitator* to target behaviour**	**Intervention components**	**Target construct (BCW)**	**Theoretical domain (TDF)**	**Intervention function (BCW)**	**Behaviour Change Technique (using BCTv1)**
**Target behaviour: Reduce scratching**					
Belief that scratching is not that bad [EO]	• Explain itch-scratch cycle and that scratching makes itch/eczema worse	Psychological capability; Reflective Motivation	Beliefs about consequences; Knowledge	Education; Persuasion	5.1 Information about health consequences<br>5.6 Information about emotional consequences
Feeling of itchiness and desire for instant relief [QI]<br><br>*Wearing appropriate clothes [QI]; Keeping cool [QI]; Emollient use [QI]; Antihistamine [QI]; Tapping [QI]; Keeping nails short/wear gloves [QI]*	• Provide information on the factors that make scratching more likely (e.g. hot temperature, lack of sleep)<br>• Provide advice on reducing itchiness (e.g. keeping cool, emollient use, tapping the area, clench fist, wearing clothes with long arms and legs, put emollient in fridge to keep cool).<br>• Provide advice on sleep management.<br>• Provide techniques to prevent the negative consequences of scratching (e.g. keep nails short, wear gloves) and how to deal with the annoyance of others telling them to stop scratching (e.g. ask people to suggest things they can do, like tap skin, instead).	Physical Capability; Psychological Capability	Environmental context and resources; Skills; Behavioural Regulation	Training; Education; Enablement; Environmental restructuring	4.1 Information on how to perform a behaviour<br>4.2 Information about antecedents<br>12.3 Avoidance/reducing exposure to cues for behaviour<br>12.5 Adding objects to the environment<br>12.6 Body changes
Scratching is a habit [QI]<br><br>*Distraction techniques [QI]*	• Provide techniques to raise awareness of scratching (e.g. record instances of scratching)<br>• Provide techniques to stop scratching (e.g. distraction, relaxation techniques, replace scratching with an alternative behaviour such as clenching fists).	Psychological Capability; Automatic Motivation; Physical Opportunity	Environmental context and resources; Skills; Behavioural Regulation	Training; Enablement; Environmental restructuring	2.3 Self-monitoring of behaviour<br>4.1 Information on how to perform a behaviour<br>8.2 Behaviour substitution<br>8.4 Habit reversal<br>12.4 Distraction<br>12.5 Adding objects to the environment
**Target behaviour: Engaging in emotional management techniques**					

For peer review only - http://bmjopen.bmj.com/site/about/guidelines.xhtml

**Barriers/*facilitator* to target behaviour**	**Intervention components**	**Target construct (BCW)**	**Theoretical domain (TDF)**	**Intervention function (BCW)**	**Behaviour Change Technique (using BCTv1)**
Belief that emotional management techniques will do little to control their eczema or help with difficult emotions [EO]/ Belief that stress (e.g. during exam time) can't be avoided [QI]<br><br>*Belief that emotions affect eczema [QI]*	• Explain the necessity of emotional management techniques for promoting engagement with the other behaviours and provide evidence that they are effective for dealing with difficult emotions	Psychological capability; Reflective Motivation	Beliefs about consequences; Knowledge	Education; Persuasion	5.1 Information about health consequences<br>5.6 Information about emotional consequences
	• Provide user quotes demonstrating how emotional management techniques helped other users to take control of their eczema	Psychological capability; Reflective motivation; Social opportunity	Beliefs about consequences; Knowledge; Social Influences	Education; Persuasion; Modelling	5.1 Information about health consequences<br>6.2 Social comparison<br>6.3 Information about others' approval<br>9.1 Credible source
Lack of understanding regarding how to do the emotional management techniques [EO]	• Provide guidance on how to do the emotional management techniques	Psychological capability	Skills	Training	4.1 Instructions on how to perform the behaviour
	• Provide guided audio recordings of emotional management exercises	Physical opportunity	Environmental context and resources	Environmental structuring	12.5 Adding objects to the environment
Lack of confidence in ability to practice emotional management techniques [EO]	• Provide YP quotes demonstrating how easy it was for other users to practice the emotional management techniques	Psychological capability; Reflective motivation; Social opportunity	Beliefs about consequences; Beliefs about capabilities; Knowledge; Social Influences	Education; Persuasion; Modelling	6.2 Social comparison<br>6.3 Information about others' approval<br>9.1 Credible source

For peer review only - http://bmjopen.bmj.com/site/about/guidelines.xhtml

Supplementary Material 2: Screenshot of page in introductory module providing key behavioural messages on topical treatments

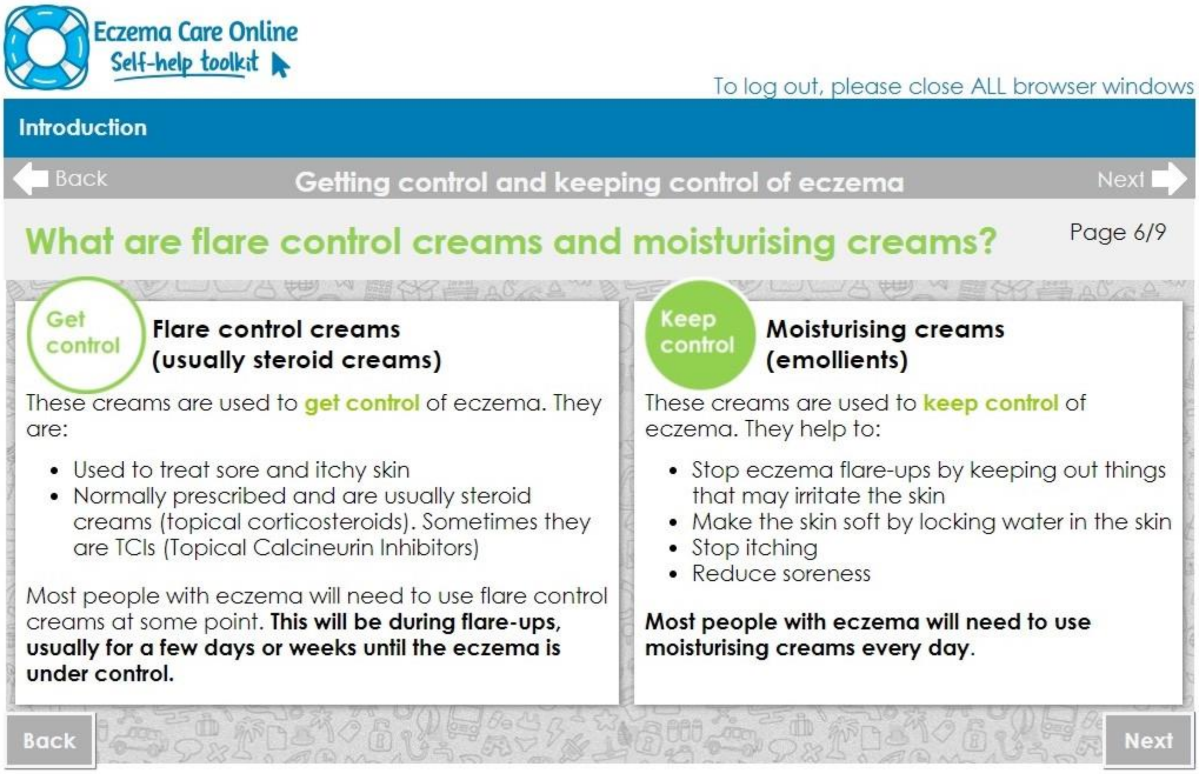

For peer review only - http://bmjopen.bmj.com/site/about/guidelines.xhtml

Supplementary Material 3: Screenshot of video explaining how eczema affects the skin barrier to provide a better understanding of eczema and a rationale for how topical treatments work

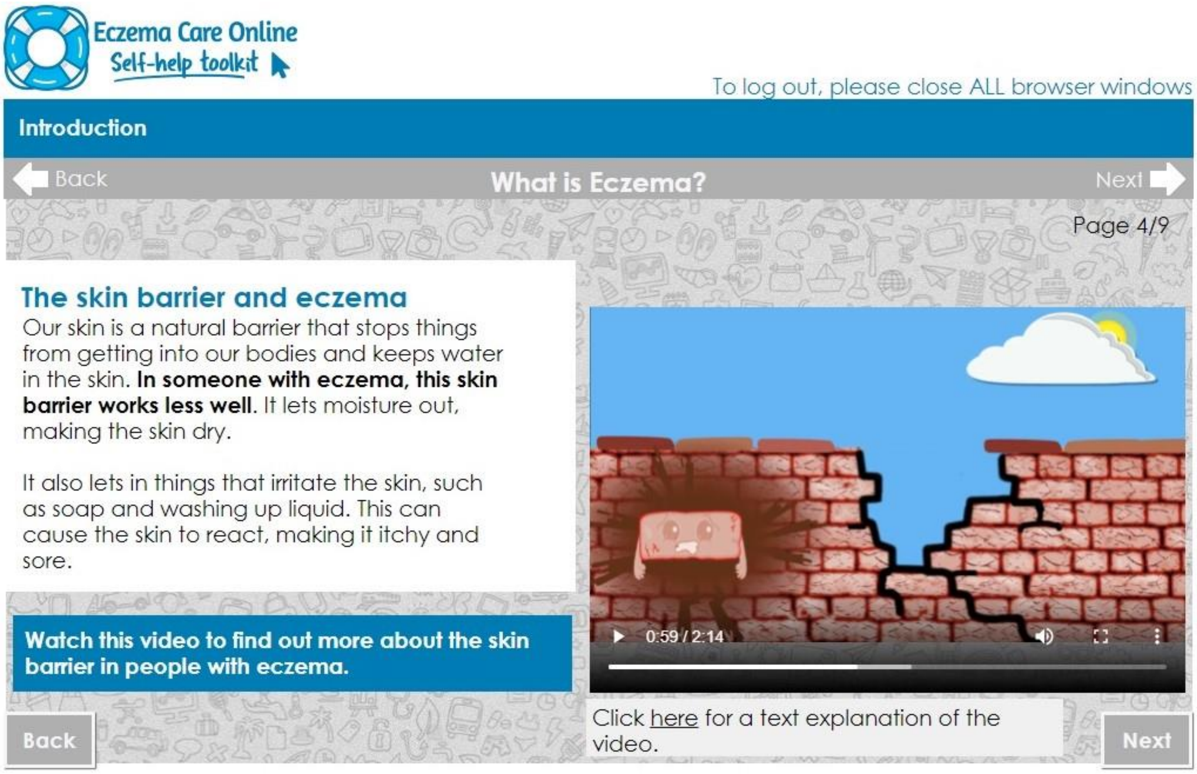

For peer review only

Supplementary Material 4: Screenshot of simple eczema assessment and subsequent feedback on which of the core treatment modules (emollients or topical corticosteroids) would be most relevant depending on whether they were currently experiencing an eczema flare-up

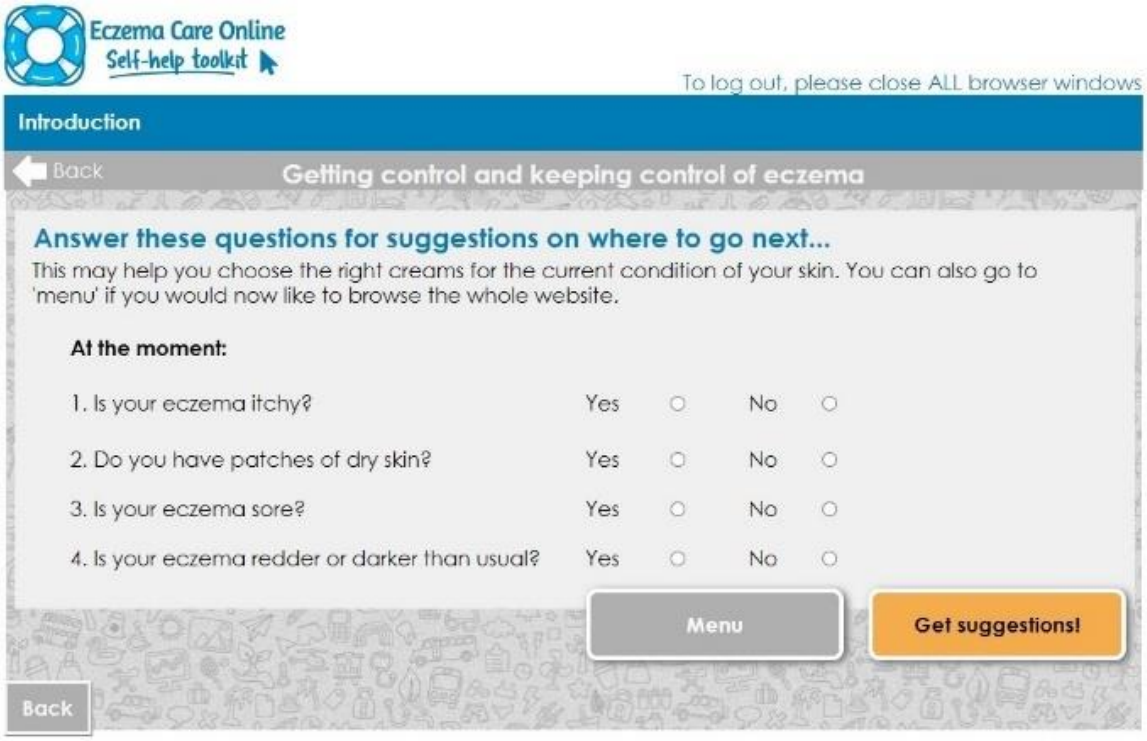

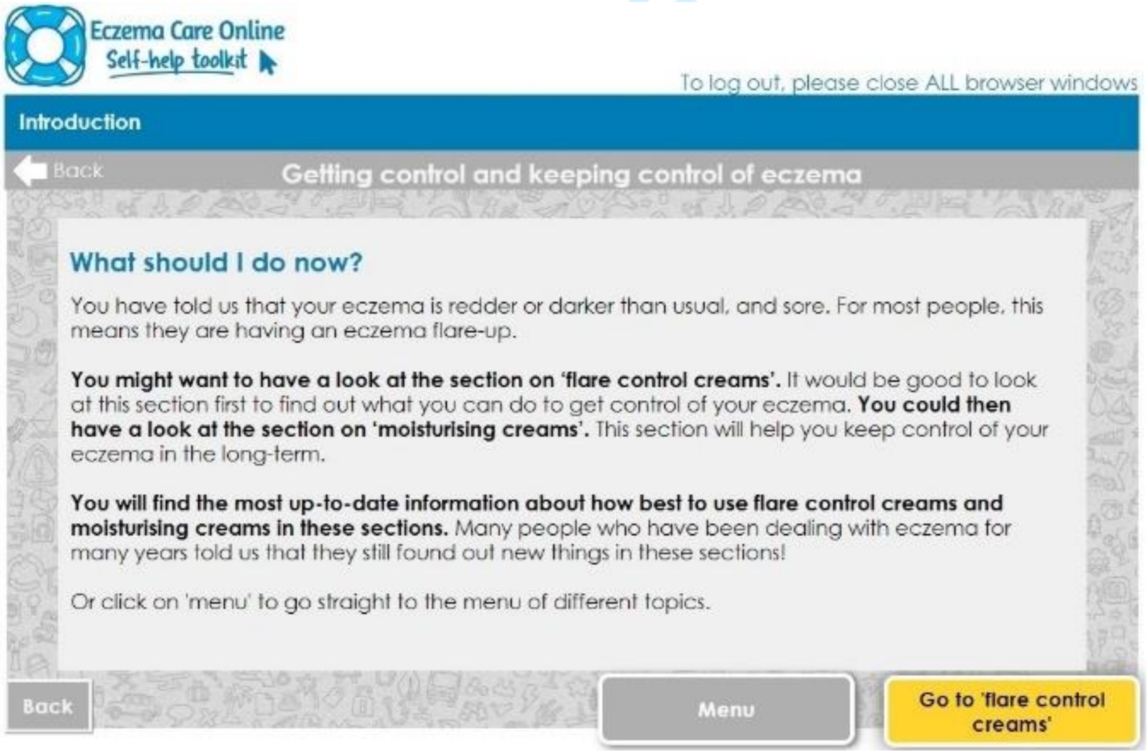

Supplementary Material 5: Screenshot of page explaining the menu of different information topics and quotes from other young people with eczema

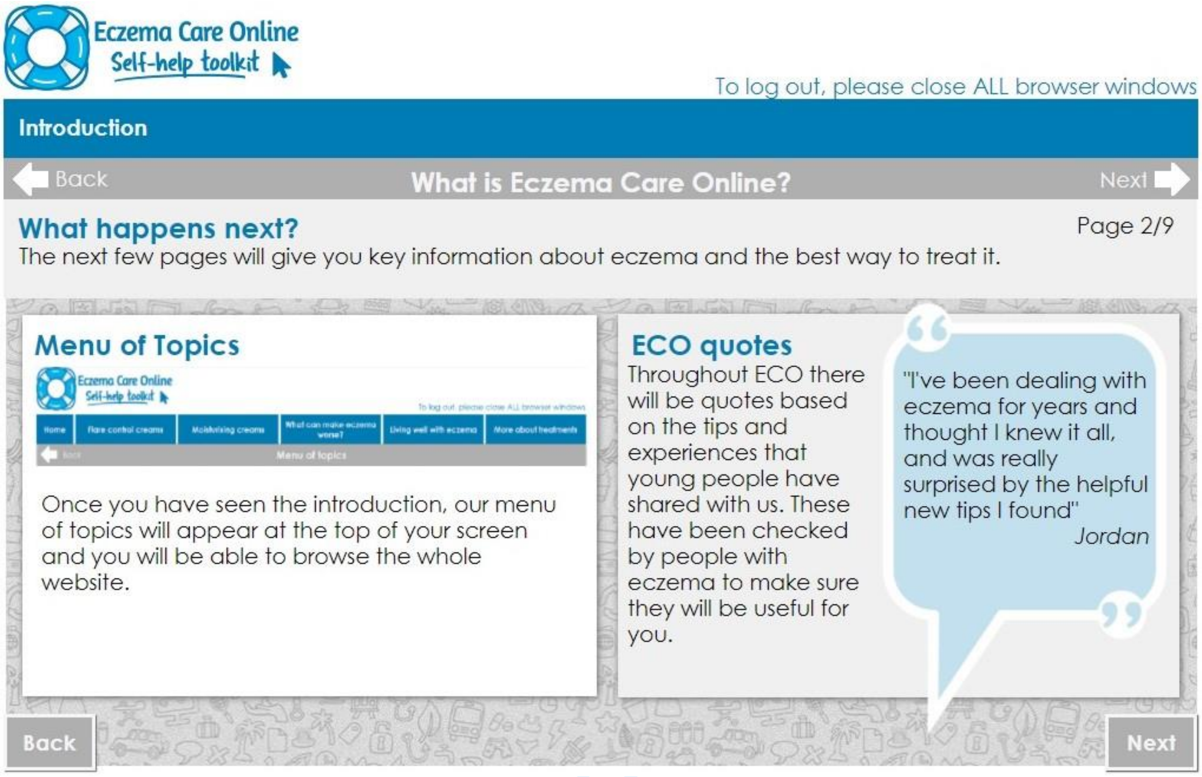

For peer review only - http://bmjopen.bmj.com/site/about/guidelines.xhtml

Supplementary Material 6: Description of Eczema Care Online (ECO) for Young People in accordance with the Template for Intervention Description and Replication (TIDieR) checklist

- 1. BRIEF NAME: Eczema Care Online (ECO) for Young People
- 2. WHY: Online interventions could potentially be used to support young people with eczema; however, research developing such interventions has been limited and systematic reviews show the need for systematically developed evidence-based interventions to support self-management of eczema.
- 3 & 4. WHAT (materials and procedures): ECO for Young People is an online behavioural intervention for young people with eczema aged 13-25 years with mild to severe eczema. It aims to reduce eczema severity, targeting 5 key behaviours: (1) Increase their use of emollients to maintain skin hydration and prevent flare-ups; (2) Improve their use of TCS or TCIs through reactive applications of these treatments in response to flare-ups or, where appropriate, regular intermittent ("weekend") preventative applications of TCS/TCI if emollients are insufficient as maintenance therapy; (3) Improve their management of irritants and triggers; (4) Improve their emotional management; and (5) Reduce scratching. See the table below for an overview of the modules included in ECO for Young People. For more detail about specific intervention techniques, see the behavioural analysis in Supplementary Material 1.

Table 1: Overview of the key intervention components of ECO for Young People

Menu	Modules	Description
Home Page	N/A	• Signposting to key modules that young people haven't accessed yet<br>• Quick links to interactive content e.g. videos
Flare control creams	• Flare control cream video<br>• What are they?<br>• Are they safe?<br>• Common questions<br>• When and how do I use them?<br>• How can I find the right cream?<br>• Golden rules	• Evidence-based education and messages to increase motivation and competence to use topical corticosteroids (TCS), including; evidence of safety and support for skills development e.g. videos/photos showing use of TCS
Moisturising creams	• Moisturising creams video<br>• What are they and how to they help?<br>• Are they safe?	• Evidence-based education and messages to increase motivation and competence to use emollients, including; evidence of safety and

For peer review only - http://bmjopen.bmj.com/site/about/guidelines.xhtml

	• Common Concerns<br>• When and how do I use them?<br>• How do I find the right cream?<br>• Take the two-week challenge<br>• Golden rules	support for skills development e.g. videos/photos showing how to use emollients
What can make eczema worse	• Living with eczema video<br>• Bathing, showering, and washing clothes<br>• Cosmetics, make-up and shaving<br>• Diet and allergies<br>• Weather and holidays<br>• Swimming and physical activity	• Evidence-based education and messages to increase motivation and competence in identifying key eczema triggers and managing them, including practical tips
Living well with eczema	• Beat the itch<br>• Sleep<br>• Stress and eczema<br>• Studying and work<br>• Eczema and money	• Evidence-based education and messages to increase motivation and competence to manage some of the symptoms and psychosocial impacts of eczema e.g. itching and scratching, disturbed sleep, stress, including techniques to manage them
More about treatments	• Talking to health professionals<br>• Infections<br>• Topical Calcineurin Inhibitors<br>• Other treatments<br>• Other resources	• Evidence-based education and messages to increase motivation and competence to manage other aspects of treatments e.g. consulting with health professionals and other treatments

- 5. WHO PROVIDED: ECO for Young People is provided direct to patients via their GP surgeries.
- 6. HOW: ECO for Young People is entirely online, although users are encouraged to contact their GP, pharmacist or nurse, for example, to obtain or change their treatments, or to treat potential infections.
- 7. WHERE: ECO for Young People is intended for any young person (aged 13-25) with mild to severe eczema. It can be implemented in primary or secondary care. The intervention is online and designed to be accessed using computers, tablets and phones via a web-browser.
- 8. WHEN and HOW MUCH: For the randomised controlled trial study, young people are given access to the intervention if their GP identifies that they have eczema that has required treatment in the last 12 months. Once registered young people can use the online intervention as much or as little as they like.

For peer review only - http://bmjopen.bmj.com/site/about/guidelines.xhtml

- 9. TAILORING: Young people have access to all the modules on ECO, however the website uses signposting based on four questions about the current state of their eczema. They will then be recommended either the module on emollients or flare control creams module depending on whether it appears they are currently experiencing an eczema flare-up. Key modules will also be recommended, one or two at a time, on the intervention home page if they haven't been viewed yet. These modules/menus are: 1) moisturising creams; 2) flare control creams, 3) bathing, showering and washing clothes; 4) beat the itch, 5) stress and eczema.
- 10. MODIFICATIONS: An additional email was circulated in response to COVID-19 that provided further advice about handwashing.
- 11. HOW WELL: The intervention is currently being tested in a randomised control trial with young people with eczema (ISRCTN79282252). Information about the results of the randomised controlled trial, including both quantitative and qualitative data, will be presented in future publications.

For peer review only - http://bmjopen.bmj.com/site/about/guidelines.xhtml

COREQ checklist

No	Item	Guide questions/description	Comments	Location in manuscript
**Domain 1: Research team and reflexivity**				
Personal Characteristics				
1.	Interviewer/facilitator	Which author/s conducted the interview or focus group?	DG, HJ, EW	Methods Section 2.2
2.	Credentials	What were the researcher's credentials? *E.g. PhD, MD*	Postdoctoral experienced qualitative researcher (DG) and two medical students (HJ, EW) supervised by DG and another postdoctoral researcher (ET)	Methods Section 2.2
3.	Occupation	What was their occupation at the time of the study?	See above	Methods Section 2.2
4.	Gender	Was the researcher male or female?	All female	Methods Section 2.2
5.	Experience and training	What experience or training did the researcher have?	See No 2	Methods Section 2.2
Relationship with participants				
6.	Relationship established	Was a relationship established prior to study commencement?	Some of the participants were known to the two medical student interviewers (HJ, EW).	-
7.	Participant knowledge of the interviewer	What did the participants know about the researcher? e.*g. personal goals, reasons for doing the research*	Participants were told that the interviews were to help improve the ECO intervention.	-
8.	Interviewer characteristics	What characteristics were reported about the interviewer/facilitator? e.g. *Bias, assumptions, reasons and interests in the research topic*	One of the interviewers was involved in intervention development (DG), which could be a potential source of bias.	-
**Domain 2: study design**				
Theoretical framework				
9.	Methodological orientation and Theory	What methodological orientation was stated to underpin the study? *e.g. grounded theory, discourse analysis, ethnography,*	Person-based approach to intervention development	Intervention development methodology section

For peer review only

No	Item	Guide questions/description	Comments	Location in manuscript
		phenomenology, content analysis		
Participant selection				
10.	Sampling	How were participants selected? *e.g. purposive, convenience, consecutive, snowball*	Purposive, opportunistic, and snowballing sampling	Methods Section 2.2
11.	Method of approach	How were participants approached? e.*g. face-to-face, telephone, mail, email*	Invitation letter from their GP practice or advertising, opportunistic and snowball sampling of students at the University of Southampton.	Methods Section 2.2
12.	Sample size	How many participants were in the study?	28	Methods Section 2.2
13.	Non-participation	How many people refused to participate or dropped out? Reasons?	Data not collected	-
Setting				
14.	Setting of data collection	Where was the data collected? e.*g. home, clinic, workplace*	At participants' homes or at a location of the participants' choice (e.g. at the university).	Methods Section 2.2
15.	Presence of non-participants	Was anyone else present besides the participants and researchers?	A family member was present for 9 of the interviews.	Methods Section 2.2
16.	Description of sample	What are the important characteristics of the sample? *e.g. demographic data, date*	Demographic information can be found in Table 2. Interviews were carried out during October 2018 to April 2019.	Methods Section 2.2
Data collection				
17.	Interview guide	Were questions, prompts, guides provided by the authors? Was it pilot tested?	Interview question guide not applicable for think aloud research	-
18.	Repeat interviews	Were repeat interviews carried out? If yes, how many?	Yes, 2 people took part in two interviews	Methods Section 2.2
19.	Audio/visual recording	Did the research use audio or visual recording to collect the data?	Audio recording.	Methods Section 2.2
20.	Field notes	Were field notes made during and/or after the	Field notes were made after all interviews.	Methods Section 2.2

No	Item	Guide questions/description	Comments	Location in manuscript
		interview or focus group?		
21.	Duration	What was the duration of the interviews or focus group?	45-90 minutes	Methods Section 2.2
22.	Data saturation	Was data saturation discussed?	We considered data saturation to be reached once no further important changes were required	Methods Section 2.2
23.	Transcripts returned	Were transcripts returned to participants for comment and/or correction?	No	-
**Domain 3: analysis and findings**				
Data analysis				
24.	Number of data coders	How many data coders coded the data?	Two (KG & DG), with input from 5 other researchers	Methods Section 2.2
25.	Description of the coding tree	Did authors provide a description of the coding tree?	No	-
26.	Derivation of themes	Were themes identified in advance or derived from the data?	Not applicable.	-
27.	Software	What software, if applicable, was used to manage the data?	Microsoft excel was used for Table of changes	-
28.	Participant checking	Did participants provide feedback on the findings?	No	-
Reporting				
29.	Participant checking	Were participant quotations presented to illustrate the themes / findings? Was each quotation identified? e.*g. participant number*	Participant quotations are presented and each quotation is identified by a pseudonym and their gender and age is noted.	Results Section 2.2
30.	Data and findings consistent	Was there consistency between the data presented and the findings?	Yes	Results Section 2.2
31.	Clarity of major themes	Were major themes clearly presented in the findings?	Not applicable.	-
32.	Clarity of minor themes	Is there a description of diverse cases or	Not applicable.	-

For peer review only

No	Item	Guide questions/description	Comments	Location in manuscript
		discussion of minor themes?		

For peer review only

BMJ Open

Eczema Care Online: development and qualitative optimisation of an online behavioural intervention to support self-management in young people with eczema

Journal:	*BMJ Open*
Manuscript ID	bmjopen-2021-056867.R1
Article Type:	Original research
Date Submitted by the Author:	24-Mar-2022
Complete List of Authors:	Greenwell, Kate; University of Southampton<br>Ghio, Daniela; The University of Manchester, Division of Psychology & Mental Health, School of Health Sciences, Faculty of Biology, Medicine and Health<br>Sivyer, Katy; University of Southampton School of Psychology; University of Portsmouth, Department of Psychology<br>Steele, Mary; University of Southampton School of Psychology<br>Teasdale, Emma; University of Southampton<br>Ridd, Matthew; University of Bristol Faculty of Health Sciences, Population Health Sciences<br>Roberts, Amanda; University of Nottingham<br>Chalmers, Joanne; University of Nottingham, Centre of Evidence Based Dermatology<br>Lawton, Sandra; Rotherham NHS Foundation Trust, Dermatology Department<br>Langan, Sinead; London School of Hygiene & Tropical Medicine<br>Cowdell, Fiona; Birmingham City University, Faculty of Health Education and Life Sciences<br>Le Roux, Emma; University of Hertfordshire<br>Wilczynska, Sylvia; University of Southampton, School of Primary Care, Population Health and Medical Education,<br>Jones, Hannah; University of Southampton<br>Whittaker, Emilia; University of Southampton<br>Williams, HC; University of Nottingham<br>Thomas, Kim; University of Nottingham, Dermatology<br>Yardley, Lucy; University of Southampton, Psychology; University of Bristol, School of Health Sciences<br>Santer, Miriam; University of Southampton, Primary Care & Population Sciences<br>Muller, Ingrid; University of Southampton, School of Primary Care, Population Health and Medical Education
**Primary Subject Heading**:	Dermatology
Secondary Subject Heading:	Qualitative research, Paediatrics, General practice / Family practice
Keywords:	Eczema < DERMATOLOGY, QUALITATIVE RESEARCH, Paediatric dermatology < DERMATOLOGY, PRIMARY CARE

SCHOLARONE™
Manuscripts

I, the Submitting Author has the right to grant and does grant on behalf of all authors of the Work (as defined in the below author licence), an exclusive licence and/or a non-exclusive licence for contributions from authors who are: i) UK Crown employees; ii) where BMJ has agreed a CC-BY licence shall apply, and/or iii) in accordance with the terms applicable for US Federal Government officers or employees acting as part of their official duties; on a worldwide, perpetual, irrevocable, royalty-free basis to BMJ Publishing Group Ltd ("BMJ") its licensees and where the relevant Journal is co-owned by BMJ to the co-owners of the Journal, to publish the Work in this journal and any other BMJ products and to exploit all rights, as set out in our licence.

The Submitting Author accepts and understands that any supply made under these terms is made by BMJ to the Submitting Author unless you are acting as an employee on behalf of your employer or a postgraduate student of an affiliated institution which is paying any applicable article publishing charge ("APC") for Open Access articles. Where the Submitting Author wishes to make the Work available on an Open Access basis (and intends to pay the relevant APC), the terms of reuse of such Open Access shall be governed by a Creative Commons licence – details of these licences and which Creative Commons licence will apply to this Work are set out in our licence referred to above.

Other than as permitted in any relevant BMJ Author's Self Archiving Policies, I confirm this Work has not been accepted for publication elsewhere, is not being considered for publication elsewhere and does not duplicate material already published. I confirm all authors consent to publication of this Work and authorise the granting of this licence.

For peer review only

Title: Eczema Care Online: development and qualitative optimisation of an online behavioural intervention to support self-management in young people with eczema

Kate Greenwell[1*], Daniela Ghio[2], Katy Sivyer[1,3], Mary Steele[1], Emma Teasdale[4], Matthew J. Ridd[5], Amanda Roberts[6], Joanne Chalmers[6], Sandra Lawton[7], Sinéad M Langan[8], Fiona Cowdell[9], Emma Le Roux[10], Sylvia Wilczynska[4], Hannah Jones[11], Emilia Whittaker[11], Hywel C Williams[6], Kim S Thomas[6], Lucy Yardley[1,12], Miriam Santer[4], Ingrid Muller[4]

[1]Centre for Clinical and Community Applications of Health Psychology, Faculty of Environmental and Life Sciences, University of Southampton, Southampton, UK; [2]Division of Psychology & Mental Health, School of Health Sciences, Faculty of Biology, Medicine and Health, University of Manchester, UK; [3]Department of Psychology, Faculty of Science and Health, University of Portsmouth, Portsmouth, UK; [4]School of Primary Care, Population Sciences and Medical Education, Faculty of Medicine, University of Southampton, Southampton, UK; [5]Population Health Sciences, University of Bristol, Bristol, UK; [6]Centre of Evidence Based Dermatology, School of Medicine, University of Nottingham, Nottingham, UK; [7]Department of Dermatology, Rotherham NHS Foundation Trust, Rotherham, UK; [8]Faculty of Epidemiology and Population Health, London School of Hygiene and Tropical Medicine, London, UK; [9]Faculty of Health, Education and Life Sciences, Birmingham City University, Birmingham, UK; [10]School of Life and Medical Sciences, University of Hertfordshire, Hatfield, UK; [11]Faculty of Medicine, University of Southampton, Southampton, UK; [12]School of Experimental Psychology, University of Bristol, Bristol, UK.

***Corresponding author**: Kate Greenwell, University of Southampton, Highfield Campus, Southampton, SO17 1BJ, UK; Email: K.Greenwell@soton.ac.uk

For peer review only - http://bmjopen.bmj.com/site/about/guidelines.xhtml

Abstract

Objectives: To describe the development of Eczema Care Online (ECO), an online behaviour change intervention for young people with eczema (Phase 1); and explore and optimise the acceptability of ECO among this target group using think-aloud interviews (Phase 2).

Methods: Theory-, evidence-, and person-based approaches to intervention development were used. In Phase 1, a qualitative systematic review and qualitative interviews developed an in-depth understanding of the needs and challenges of young people with eczema. Guiding principles highlighted key intervention design objectives and features to address needs of this target group to maximise user engagement. Behavioural analysis and logic modelling developed ECO's hypothesised programme theory. In Phase 2, qualitative think-aloud interviews were carried out with 28 young people with eczema and the intervention was optimised based on their feedback.

Results: The final intervention aimed to reduce eczema severity by supporting treatment use (emollients, topical corticosteroids/topical calcineurin inhibitors), management of irritants/triggers, emotional management; and reducing scratching. Generally, young people expressed positive views of intervention content and design in think-aloud interviews. Quotes and stories from other young people with eczema and ECO's focus on living with eczema (not just topical treatments) were valuable for normalising eczema. Young people believed ECO addressed knowledge gaps they had from childhood and the safety information about topical corticosteroids was reassuring. Negative feedback was used to modify ECO.

Conclusions: A prototype of the ECO intervention was developed using rigorous and complementary intervention development approaches. Subsequent think-aloud interviews helped optimise the intervention and demonstrated ECO is likely to be acceptable to this

For peer review only - http://bmjopen.bmj.com/site/about/guidelines.xhtml

target group and provided support for our guiding principles including key design objectives and features to consider when developing interventions for this population. A randomised controlled trial and process evaluation of the intervention is underway to assess effectiveness and explore user engagement with the intervention's behavioural goals.

Article Summary

Strengths and limitations of this study

- Our rigorous development using complementary theory-, evidence-, and person-based approaches to intervention development helped ensure the intervention was acceptable and engaging to our sample of young people with eczema.
- Our multidisciplinary intervention development group, including Patient and Public Involvement, ensured that the content was evidence-based, that advice was feasible, and that the perspectives of people living with eczema were considered throughout the whole development process.
- Although we were able to recruit a good range of ages, genders and eczema severities for the think-aloud interview study, a large proportion of the sample were university students, so were likely to have high levels of health and computer literacy.
- As think-aloud interviews explore participants' immediate reactions to the intervention, they cannot tell us how people would use the intervention over time or explore engagement with the wider behavioural goals.

Manuscript word count: 4269

For peer review only

Introduction

Atopic eczema (syn. atopic dermatitis) is the most common type of dermatitis/eczematous inflammation and will be referred to from here on as just "eczema" in accordance with the nomenclature of the World Allergy Organisation.[1] Eczema is a common skin condition that usually begins in childhood,[2] but for many the symptoms (dry, sore, itchy skin) can persist into adolescence and adulthood.[3]. Eczema management focuses on identification and avoidance of irritants/triggers that may exacerbate eczema symptoms; regular use of emollients to restore skin barrier function; and topical corticosteroids or topical calcineurin inhibitors (TCIs) to treat flare-ups.[4]

Eczema management can be particularly challenging in adolescence and early adulthood. Young people report a lack of knowledge regarding their eczema and treatments, and advice provided can often conflict with their own eczema experiences and a competing desire to maintain a 'normal' adolescent life.[5–9] These factors may explain why adherence to topical treatments presents challenges for this age group and many report not using their treatments as prescribed.[7]

Systematic reviews of self-management interventions for people with eczema[10,11] identified that, although a few studies evaluated interventions have been developed for parents/carers of children with eczema, only two studies evaluated interventions for children and adolescents.[12,13] Both interventions were delivered face-to-face and only one reported tailoring their intervention to this age group.[12] To address this research gap and the need to improve self-management support for people living with eczema, the Eczema Care Online (ECO) programme aimed to develop two online behavioural interventions: one for young people with eczema (13-25 years) and one for parents and carers of children (0-12 years) with eczema (www.nottingham.ac.uk/eco).[14,15]

For peer review only - http://bmjopen.bmj.com/site/about/guidelines.xhtml

First, we aimed to describe the development of the ECO intervention for young people with eczema (Phase 1). Second, we aimed to explore and optimise the acceptability of the ECO intervention among young people with eczema (Phase 2). This article highlights key psychosocial needs of young people with eczema and intervention features to consider when developing behavioural interventions for this group.

Intervention development methodology

We used theory-, evidence-, and person-based approaches to develop the online intervention.[16–18] Guidelines for developing complex interventions emphasise that interventions should be informed by reviews of the current evidence-base, appropriate theory, and an in-depth understanding of the context in which the intervention will be implemented.[19] The Person-Based Approach to intervention development uses iterative qualitative research to understand and accommodate the perspectives of the intervention's target group.[20]

Intervention development was carried out in two phases. In Phase 1, we collated and synthesised evidence relating to patient behaviours that are likely to reduce eczema severity and the perspectives of young people with eczema (person- and evidence-based approach). This evidence guided decisions regarding the intervention's target behaviours and provided us with an in-depth understanding of the key issues, needs, and behavioural challenges of this target group. Theory-based approaches (behavioural analysis and logic modelling) were used to develop and illustrate the intervention's hypothesised programme theory, that is, the hypothesised mechanisms of action by which the intervention components exerts their effects.[19,21] In Phase 2, we carried out iterative qualitative think-aloud interviews to gather user feedback on the intervention prototype and optimise it based on this feedback.[22] The methods and results for each phase are reported below.

For peer review only - http://bmjopen.bmj.com/site/about/guidelines.xhtml

Both phases were guided by a multidisciplinary intervention development group, which comprised 18 members including Patient and Public Involvement (PPI), dermatologists, a nurse consultant, researchers with an interest in eczema, GPs, health psychologists, and experts in intervention development, writing patient-friendly health information, and long-term conditions in adolescents. Through regular meetings and reviewing documents, this group guided the design of the research, helped with the interpretation of the research findings, and provided detailed feedback on the intervention plans, written content, website design, and prototypes for both online interventions (young people and parents and carers of children with eczema). The intervention development process is illustrated in Figure 1. Ethical approval for this research was granted by Wales REC 7 Ethics Committee (REC 17/WA/0329).

Patient and Public Involvement

Two mothers of children and young people with eczema (one of whom had eczema herself and helps run an eczema support group [AR]) were part of our multidisciplinary intervention development group. Both were co-applicants on the research grant application, helping to identify the research topic and develop research questions. We also sought additional PPI feedback on the intervention content and design from two young people with eczema and a panel of PPI contributors with an interest in skin research, most of whom had experience of eczema, and some were aged 18-25. We discuss the specific contributions of the PPI to intervention development throughout this manuscript. Two young people reviewed the study participant information sheet to check comprehension. One PPI member (AR) discussed and provided feedback on our interpretations of the findings and this manuscript. She continues to help us to disseminate our research findings among her wide-reaching patient networks and via social media.

For peer review only - http://bmjopen.bmj.com/site/about/guidelines.xhtml

Phase 1: Intervention planning

Phase 1 Methods

Phase 1 comprised of three steps: (1) Defining the intervention target behaviours; (2) Collating and synthesising evidence relating to the perspectives of young people with eczema; and (3) Creating an intervention plan.

1.1 Defining the intervention target behaviours

The multidisciplinary intervention development group agreed the intervention's key target behaviours through consideration of the evidence-base for effective eczema treatments, clinical guidance on eczema management and recommendations around what would be feasible and acceptable to implement through an online intervention.[4,23,24]

1.2 Collating and synthesising evidence relating to the perspectives of young people with eczema

First, we undertook a systematic review and thematic synthesis of the views and experiences of eczema and/or eczema self-care of people with eczema and parents or carers of children with eczema.[8] Qualitative studies focusing on the views and experiences of eczema and eczema treatments, and barriers and facilitators to eczema self-management were included. The review identified 39 papers (reporting 32 studies; 9 including young people in the sample).

Second, we carried out a secondary analysis of interview data from 25 young people (17-25 years) with eczema.[5,6,25] The data came from the SKINS project, which explored young people's experiences of living with common skin conditions (eczema, acne, psoriasis, alopecia). To gather additional views from younger adolescents, the ECO study added to this data set by carrying out interviews with five young people with eczema aged 13-16 years.

For peer review only - http://bmjopen.bmj.com/site/about/guidelines.xhtml

Both interview studies explored young people's views about eczema treatment and management, and information and support needs. Interviews were analysed using inductive thematic analysis.

The methods and findings of these studies have been described in detail elsewhere.[5,6,8,25] Only key findings relevant to intervention development are summarised in this paper, with the focus of this paper being on intervention development and the novel findings from Phase 2.

1.3 Developing an intervention plan

Consistent with the person-based approach, our in-depth understanding of young people with eczema informed the development of *guiding principles*, which outlined key intervention objectives and design features that will address these.[20] A list of potential barriers and facilitators to the target behaviours were also identified from this evidence-base and from consultation with the multidisciplinary intervention development group and additional PPI representatives. A *behavioural analysis* outlined the intervention components that were added to address each of the identified barriers and facilitators for each target behaviour. Consistent with the approach taken by Band et al. and Greenwell et al.,[17,18] these components were mapped onto behaviour change theoretical frameworks to describe the planned intervention content and identify hypothesised mechanisms of action. The Behaviour Change Techniques Taxonomy classifies intervention content by their behaviour change techniques, the smallest component for changing behaviour.[26] The Behaviour Change Wheel was used to classify the source (component of the COM-B model hypothesised to influence behaviour; Capability, Opportunity, Motivation) and function (e.g. 'Education', 'Persuasion') of each individual or group of behaviour change techniques.[27] We also mapped the behaviour change techniques

For peer review only - http://bmjopen.bmj.com/site/about/guidelines.xhtml

onto their theoretical constructs (e.g. ‘Knowledge’, ‘Skills’) using the Theoretical Domains Framework,[28] which is recommended for use alongside the Behaviour Change Wheel.

To illustrate key elements of the intervention’s programme theory, a *logic model* was developed to illustrate how the intervention components, theoretical constructs (intervention processes), and key behaviours (purported mediators) influence the intervention outcomes (eczema severity).

Phase 1 Results

1.1 Defining the intervention target behaviours

The multidisciplinary intervention development group agreed that ECO would aim to reduce eczema severity by supporting young people with eczema to: (1) Increase their use of emollients to maintain skin hydration and prevent flare-ups; (2) Improve their use of topical corticosteroids or topical calcineurin inhibitors through reactive applications of these treatments in response to flare-ups or, where appropriate, regular intermittent (“weekend”) preventative applications of topical corticosteroids or topical calcineurin inhibitors if emollients are insufficient as maintenance therapy; (3) Improve their management of irritants and triggers; (4) Improve their emotional management; and (5) Reduce scratching. Use of emollients and topical corticosteroids/topical calcineurin inhibitors were identified as core behaviours that would likely have the greatest effect on eczema severity. Therefore, intervention content relating to these behaviours was deemed the most important.

1.2 Collating and synthesising evidence relating to the perspectives of young people with eczema

The qualitative evidence helped us to develop the following understanding of our target group. Young people are developing a sense of independent identity and, specifically, young people with eczema are keen to take on more responsibility with their eczema

For peer review only - http://bmjopen.bmj.com/site/about/guidelines.xhtml

management.[25] However, these young people may find their new roles and responsibilities, such as interacting with health professionals and negotiating health care systems, daunting.[25] Young people do not have a comprehensive understanding of eczema, specifically some have little knowledge of the causes/triggers of eczema and the rationale behind their treatment (e.g. difference between emollients and topical corticosteroids/topical calcineurin inhibitors and how to use them).[5,8] Health professionals do not always revisit such information, assuming that young people were told this information in childhood.[5,8] In general, young people perceive topical treatments to be effective, but they also have doubts about their long-term effectiveness, and concerns around their safety and becoming over-reliant on topical corticosteroids.[8,25] This group report several perceived barriers to applying topical treatments, including using treatments when outside of the home (e.g. when in class or working in a public-facing job) and cost of treatments.[8,25] These treatment barriers are not unique to this age group,[8] but are nonetheless important to address for any eczema behavioural intervention.

Many young people were told in childhood that they would ‘grow out of’ eczema, but this information is often at odds with their own experiences.[5] Young people have a desire to ‘fit in’ with their peers and feel like a ‘normal’ young person.[6] They welcome the opportunity to share experiences with other young people with eczema to normalise their experience.[6]

The multidisciplinary intervention development group discussed what specific website design needs young people may have. Usability research has shown that this group are likely to dislike reading large amounts of text, like concepts to be illustrated visually, and relate to content created by peers (e.g. stories, images, examples from other young people).[29,30] This evidence was supported by discussions with our young people PPI who also suggested that young people may prefer videos over reading lots of text and recommended having brief ‘top tips’ with suggestions from other young people for how they manage eczema. They also

For peer review only - http://bmjopen.bmj.com/site/about/guidelines.xhtml

felt that it was important that the intervention could be accessed via a mobile device or computer.

1.3 Developing an intervention plan

We developed a set of guiding principles to address the issues identified in the evidence synthesis stage (1.2) (Table 1). The behavioural analysis table is presented in Supplementary Material 1 and the logic model in Figure 2.

Phase 2: Intervention optimisation

Phase 2 Methods

2.1 Creating the intervention prototype and videos

Creating the intervention prototype was done in several stages. First, guided by our target behaviours, guiding principles and qualitative research, the multidisciplinary intervention development group agreed the topics of the intervention modules and videos. Second, we wrote page content and video scripts, and circulated this to the multidisciplinary intervention development group for comment to ensure it was evidence-based and medically accurate, and the advice was clear and feasible. Positive and negative feedback was entered into the Person-Based Approach Table of Changes,[16] and potential changes were discussed, agreed, and prioritised.

We tested either the video scripts, audio recordings of the scripts, or a storyboard or prototype of the video with young people using think-aloud interviews, and these were also reviewed by a PPI panel. Once the written content and videos were finalised, we created a working prototype of the intervention using the LifeGuide software,[31] which was reviewed by our two young people PPI and further optimised through think-aloud interviews with young people with eczema. The final videos were created by an external video creator.

For peer review only - http://bmjopen.bmj.com/site/about/guidelines.xhtml

Table 1 Guiding principles for ECO for young people

User context	Intervention design objectives	Key intervention features
• Young people (YP) with eczema have an increasing desire for autonomy regarding their eczema management, but may feel apprehensive about their new roles and responsibilities.<br>• YP may have gaps in their understanding of eczema.<br>• YP may perceive barriers to using topical treatments.	To support YP to gain autonomy and competence in their eczema management.	• Ensure YP have a complete understanding of eczema and the rationale behind their treatment.<br>• To build YP's self-efficacy for the target behaviours (e.g. information on how to apply treatments, avoid irritants/triggers, reduce scratching).<br>• Stories and tips from other YP on what helped them take control of their own eczema and how to overcome barriers to treatments.<br>• Use autonomy-promoting language, provide choice wherever possible, and avoid condescending or 'child-like' language/graphics.<br>• Provide advice on how to communicate with health professionals and make the most out of appointments.
• YP have a desire to live as 'normal' a life as possible.<br>• YPs receive unhelpful messages that eczema is solely a childhood illness	To enable YP to maintain a sense of normalcy when managing their eczema	• Provide age-appropriate advice on living with eczema (e.g. shaving, wearing make-up, and managing eczema at work/university/school).<br>• Provide relatable stories and advice from other YP with eczema.<br>• Acknowledge that, for some, eczema persists into adolescence and adulthood.<br>• Provide images and descriptions of eczema for different skin-types.<br>• Avoid providing overly restrictive advice on irritants/triggers, instead offering advice on how to minimise the negative consequences of exposure irritants and triggers or provide alternatives (e.g. using emollients in place of soap).

For peer review only - http://bmjopen.bmj.com/site/about/guidelines.xhtml

• YPs may have doubts and safety concerns about topical treatments.<br>• YP may find topical treatments unpleasant in texture and/or smell and they worried about applying treatments in public in case others 'found out' that they had eczema.	To build YP's beliefs in the positive effects of their topical treatments	• Provide information to address topical treatment concerns and barriers, and persuade YPs of the long-term effectiveness of these treatments.
• YPs prefer content that is easy to scan, visual, and peer-created.<br>• YPs want interventions that are accessible on their mobile devices and computers.	To provide engaging and accessible intervention content	• Provide interactive content (e.g. quizzes), videos and pictures, and reduce reading burden by keeping the amount of text per page to a minimum.<br>• Break the content down into lots of short sections/modules.<br>• Intervention to be mobile-friendly.<br>• Provide peer-created content (e.g. stories, videos)

For peer review only

2.2 Think-aloud interviews

We carried out 30 think-aloud interviews with 28 young people with eczema (2 people took part in two interviews, viewing a later intervention version) who were purposively sampled based on age, gender, and eczema severity (Table 2). Twenty-three of these participants were newly recruited for this study and five participants were from the qualitative study in Phase 1. Participants were recruited via an invitation letter from their GP practice (n=20) or advertising, opportunistic and snowball sampling of students at the University of Southampton (n=8). During the think-aloud interviews, participants were asked to use sections of the intervention while sharing their thoughts and reactions to the content and design aloud. Interviews were facilitated by a researcher who observed the participant using the intervention and asked prompts when needed to elicit participant reactions. At the end of the think-aloud interview, participants were asked some open-ended questions to elicit their general views of the intervention content and design and how it compares to other websites they have used.

To maximise participant time during the interviews, participants testing optional modules were sent the core intervention content to look through before the interview and they were asked about it during the interview. Interviews were carried out at participants' preferred location (e.g. at home, the university) and relatives were present for nine interviews. Interviews lasted 45-90 minutes, took place between October 2018 to April 2019, and were carried out by DG and two medical students (HJ, EW) and one postdoctoral student were trained and supervised by DG and ET (postdoctoral experienced qualitative researchers) (all females). Interviews were audio-recorded and transcribed verbatim.

Optimisation was iterative, moving between data collection, analysis, and intervention modification. We considered data saturation to be reached once no further important changes

For peer review only - http://bmjopen.bmj.com/site/about/guidelines.xhtml

were required.[16] For each interview, DG completed a feedback table, highlighting positive and negative comments about the intervention, based on the interviewer field notes and listening back to the audio recordings.

Table 2 Think-aloud participant demographics

Variable	Statistic
**Age**	
Mean (SD)	17.82 (3.41)
Range	13-23
**Gender**	
Female N (%)	13 (46.43)
Male N (%)	15 (53.57)
**Eczema severity (self-defined)***	
Mild N (%)	10 (38.46)
Mild/Moderate N (%)	2 (7.69)
Moderate N (%)	7 (26.92)
Moderate/Severe N (%)	3 (11.54)
Severe N (%)	4 (15.38)

Key: **n*=26

MSa and IM read through the transcripts to ensure important issues were captured. The feedback table was reviewed by a sub-group of the intervention development group (KG, IM, MSa, KS, MSt, ET) at weekly meetings. KG transferred negative comments into the Table of Changes and potential changes were agreed and prioritised by this sub-group.[16]

Phase 2 Results

2.1 Creating the intervention prototype and videos

As the primary focus of the intervention was educational, a website that was also accessible via a mobile device was deemed the most appropriate delivery format. In the final intervention prototype, users first progressed through a brief (nine pages) introductory section

For peer review only - http://bmjopen.bmj.com/site/about/guidelines.xhtml
For peer review only

containing the key behavioural messages necessary for facilitating a basic understanding of eczema and its management (Supplementary Material 2). Some key behavioural messages were also summarised in short (2 minute) videos (Supplementary Material 3). Users then had the option of completing a simple eczema assessment that provided advice on which of the core treatment modules (emollients or topical corticosteroids) would be most relevant to them, depending on whether they were currently experiencing an eczema flare-up (Supplementary Material 4). Users had access to a menu where they could choose the topic modules that were most relevant to them (Supplementary Material 5). On each login, users were given suggestions for which modules to look at next, based on what they had looked at previously. Users could choose to have additional behaviour change content delivered by email or SMS text messages.

A key design feature highlighted in the guiding principles was the use of quotes from other young people with eczema sharing their experiences of eczema and eczema management advice (Supplementary Material 4). We also introduced new terminology to help young people better understand the function of each type of topical treatment and to mirror how this group already referred to these treatments (e.g. ‘creams’ for all treatments, regardless of whether they were actually a cream or an ointment). Emollients were termed ‘moisturising creams’ and topical corticosteroids/topical calcineurin inhibitors were termed ‘flare control creams’.

Our multidisciplinary intervention development group and PPI representatives felt that it was important that the intervention is accessible and relevant to all ethnic groups. Therefore, ECO provided images and descriptions of eczema for different skin-types and our videos included cartoon characters from different ethnic groups. The full intervention content is outlined in Supplementary Material 6 using the TIDieR checklist.[32]

For peer review only

For peer review only - http://bmjopen.bmj.com/site/about/guidelines.xhtml

2.2 Think-aloud interviews

Generally, young people expressed positive views of the intervention's content and design. They found the information and advice clear, easy to follow, helpful, and relatable, and they liked the videos and brief eczema assessment. Specifically, they found the quotes and advice from other young people with eczema, and the facts about how common it is to still have symptoms at their age reassuring, personal, and it made them feel less alone.

> *"I think it's quite comforting to actually know that it's normal for people with eczema. As in like this is, it's not just me, because even though you get told loads of people have eczema, it is just quite nice to be like, 'This is what you're going through, it's okay, this is what you're going to do about it,' and just have the information all there." (P10, 19-21 years old)*

> *"I like this page [content on prevalence of eczema among young people]…it's good to know that, like, I don't know, I always feel quite weird, because I'm 21 and still have eczema, so, it's good to know that." (P17, 19-21 years old)*

Young people valued that ECO provided advice on living well with eczema, rather than focusing solely on medical treatments.

> *"Sleep problem, emotion, diet and how much you eat - that's good because it's saying that it's not just eczema like using creams, it's also got other stuff, other aspects to it as well…I think you can't just control eczema with creams, you've got to control the whole lot. Yes, this is useful. (P13, 19-21 years old)*

> *"In lighter skin eczema may look red, in darker skin eczema may look grey, purple or brown'. I feel like saying that is actually good, because a lot of the time I'm just like, why does my eczema look grey? But I didn't even know that, now I can see that's a*

For peer review only - http://bmjopen.bmj.com/site/about/guidelines.xhtml

common thing in darker skin. I feel like having that is actually really good. (P22, 16-18 years old)

"I think that [module on itching] was the most helpful because it said apart from using the creams and things, other things you can do to help you. (P19, 13-15 years old)

Most young people explained how they learned something new about eczema and its management from the part of the website they used, with some explaining that ECO helped addressed the knowledge gaps from childhood.

"[ECO is] brilliant. It's given me a lot more information than I've ever had in the past…I've learnt a lot of new things today about eczema that I didn't know over the last 22 years of having eczema, it's amazing!...It's amazing, I love it." (P9, 22-25 years old)

"If I was prescribed stuff by my doctor I would be like, 'Well, it's probably good for me.'...I'd be like why am I even using this and then not bother. I think if you knew that, okay this one's going to stop you itching and reduce soreness then you might be more likely to carry on using it and also, yes if you're someone that doesn't really ask questions it's nice to have a place that will tell you the information anyway without you having to ask someone. (P18, 16-18 years old)

Young people also talked about how they found the information about the safety of topical corticosteroids reassuring:

"That's good to know, that it [topical corticosteroids] doesn't affect growth or development…because I've been using it for so many years now. It's always been on my mind, and not being able to find out any information about it, it's worried me…but

For peer review only - http://bmjopen.bmj.com/site/about/guidelines.xhtml

this helps a lot. It kind of puts your mind at ease, knowing that it doesn't do anything to your growth or development." (P9, 22-25 years old)

The positive feedback provided support for our guiding principles, which emphasised the importance of maintaining a sense of normalcy when managing eczema, addressing knowledge gaps to support young people to gain autonomy and competence in eczema management, and providing engaging and accessible intervention content (Table 1). The negative feedback was used to modify the intervention (Table 3 outlines some example negative comments and the changes implemented).

Discussion

This manuscript provides a description of the rigorous development process for a behavioural intervention for young people with eczema; a target group that has been largely ignored in eczema research.[8,10,11] It is essential to provide self-management support to this age group as they begin to take on a more active role in their eczema management; a role which was previously taken on by their families.[7] The person-based approach to intervention development allowed us to understand and accommodate the perspectives of young people with eczema, resulting in an online intervention that was engaging and acceptable to our sample of this target group.[20]

The behavioural analysis maintained a focus on the behaviours most likely to influence eczema severity (topical treatment use, managing irritants/triggers, scratching, emotional management) and suggested acceptable behaviour change techniques that can help young people with eczema overcome behavioural barriers relevant to them. Logic modelling offered a programme theory that can be tested and refined in future process analyses. Our final guiding principles outlined some of the key behavioural issues, intervention design objectives, and design features that those developing behavioural interventions for young

For peer review only - http://bmjopen.bmj.com/site/about/guidelines.xhtml

people with eczema, or other long-term conditions, may wish to consider to maximise user engagement. The think-aloud interviews provided support for the relevance of these guiding principles to this target group and the acceptability of the design features we implemented to engage this group. Specifically, the ECO intervention's use of quotes and tips from other young people with eczema and its focus on living with eczema (not just topical treatments) were valuable for normalising eczema.

Table 3 Example issues identified from the think-aloud interviews and the changes implemented to address these

**Summary of issue identified**	**Example quote**	**Change implemented**
Some found the questions in the brief eczema assessment confusing.	*"'Does your skin feel dry?' Well, everywhere or in general where you've got...eczema? ...I don't know if that's [the question] very clear." (P12, 22-25 year old)*<br><br>*"'Is your skin or redder or darker than usual?'...to me it sounds like 'is your eczema darker or less dark than your eczema normally is?', but I think it's a bit ambiguous whether it means that or their usual skin?...you could just say 'is your eczema red or darker than usual?'" (P18, 16-18 years old)*<br><br>*'Is your skin redder or darker [than usual]?'...maybe it's the 'than usual' bit that needs to be more clearer, because I could interpret that as this being my usual skin, rather than the usual colour of the eczema...maybe, 'Is your eczema redder or darker than usual?' or something (P5, 19-21 years old)*	The assessment questions were reworded to clarify that we are asking about their eczema at present (rather than in the past) and their eczema skin (rather than skin in general).

For peer review only - http://bmjopen.bmj.com/site/about/guidelines.xhtml

Summary of issue identified	Example quote	Change implemented
The feedback for the brief eczema assessment did not always match their experience (e.g. feedback suggested they may have an eczema flare-up when they didn't).	*"[Is your skin itchy or sore?] it's not really sore but it is itchy…I can have itchy dry skin but I only have sore skin when it's red and inflamed…[recommended the flare control creams module]… it said that I'm having a flare-up now, but I wouldn't class this as a flare-up. This is kind of just my - how my eczema kind of bobbles along, as in my flare-ups would be much more aggressive than what they were suggesting it is." (P10, 19-21 years old)*	We separately asked whether eczema is 'itchy' and 'sore' so that people do not receive feedback that they are having a flare-up if they just have itchy and not sore skin. Soften the feedback on current eczema severity to avoid disengaging those who don't agree that they are experiencing an eczema flare-up ('For most people, this means they are having an eczema flare-up').
Some thought the introductory section was too long.	*"Personally, if I see there's 21 pages, I'm just going to try and get through them." (P18, 16-18 years old)*	Reduce the introductory section significantly with optional click-outs to additional information if needed.
Some young people commented that they already knew a lot of the information as they have had eczema for a long time.	*"[ECO] was really good…because I've had it for so long I feel like a lot of the information there I've already had drilled into me my whole life, but I feel like for people that either have children with eczema or people with eczema, I don't know, I think it would be really helpful... personally, I would probably want the more information about the lifestyle stuff and diet" (P10, 19-21 years old)*<br><br>*"I think a lot of stuff I probably kind of already knew from having eczema but it's good to get a few things that help yes" (P19, 13-15 years old)*	Added in a quote from another young person who had eczema for a long time saying how they were surprised that the website contained new helpful tips. Emphasise that the information is based on the most up-to-date research evidence (so they may find new information) and that there is information on 'living with eczema' (e.g. diet advice).

For peer review only

The value of behaviour change interventions that make individuals feel they are ‘not alone’ is supported by other qualitative research with people with long-term conditions and parents/carers of children with eczema.[23,33] Although this intervention focused mainly on treatment adherence as this was deemed to have the biggest influence on eczema severity, providing age-appropriate advice and support on how young people can better live with eczema was valuable for ensuring the content was engaging and relatable to this target group. Young people explained how valuable the explanations of eczema and rationale behind the topical treatments for addressing gaps in their knowledge, with most participants reporting that they learned something new from ECO. This provided further support for the need for health services to revisit eczema education with young people, avoiding assumptions that they have already been told this information.[5] Online information can be confusing and of variable quality,[34,35] therefore, it is important to signpost young people to high quality evidence-based online information so they are empowered to take an active role in their own healthcare.[36]

A strength of this research was our rigorous development using complementary theory-, evidence-, and person-based approaches to ensure the intervention is acceptable and engaging to its target group. Our multidisciplinary intervention development group and PPI ensured that the content was evidence-based, that advice was feasible, and that the perspectives of people living with eczema were considered throughout the whole development process.[37] It was helpful to gain iterative feedback from PPI and young people with eczema on early versions of the videos (i.e. scripts, audio recordings of the video voiceover, prototype) to ensure that they were as acceptable as possible to the target group before it was finalised by the external video creator. One limitation of the think-aloud interview study is that we did not collect information on participant ethnicity, socioeconomic status, or educational level. It will be important to purposively sample based on these demographics in future evaluations of this

For peer review only - http://bmjopen.bmj.com/site/about/guidelines.xhtml

intervention to ensure it is engaging and effective for all participant groups and ensure digital interventions do not further facilitate healthcare inequalities. Furthermore, as think-aloud interviews explore participants' immediate reactions to the intervention, they cannot tell us how people would use the intervention over time or explore engagement with the target behaviours.

In conclusion, the think-aloud interview study demonstrated ECO is likely to be acceptable to young people with eczema and provided support for our guiding principles, including key design objectives and features to consider when developing interventions for this population. A randomised controlled trial of ECO to explore effectiveness and cost-effectiveness is underway (ISRCTN79282252).[14] This includes a nested mixed-methods process evaluation to explore young people's experiences of using the intervention over time and how the intervention was used, and test and further refine the intervention's programme theory. For the qualitative interview study, we purposively recruited participants across a range of ages, ethnicities, socioeconomic status, eczema severities, and intervention usage.

Acknowledgments

We would like to thank our young people PPI Taheeya and Tahmid Ahmed and the panel of public contributors for their valuable feedback on the Eczema Care Online intervention.

Funding

This article presents independent research funded by the National Institute for Health Research (NIHR) under its Programme Grants for Applied Research (PGfAR) Programme (Grant Reference Number RP-PG-0216-20007). Eczema Care Online (ECO) was developed using the LifeGuide software, which was partly funded by the NIHR Biomedical Research Centre (BRC), Southampton. The views expressed are those of the authors and not

For peer review only - http://bmjopen.bmj.com/site/about/guidelines.xhtml

necessarily those of the NIHR or the Department of Health and Social Care. SML is funded by a Wellcome Senior Clinical Research fellowship [205039/Z/16/Z]. This research was funded in whole or in part by the Wellcome Trust [205039/Z/16/Z]. For the purpose of Open Access, the author has applied a CC BY public copyright licence to any Author Accepted Manuscript (AAM) version arising from this submission.

Competing interests

SML is a co-investigator on the IMI Horizon 2020 project BIOMAP, but is not in receipt of industry funding. The other authors have no competing interests to declare.

Author Contributions

KG, DG, KS, MSt, ET, MJR, AR, JC, SL, SML, FC, SW, HCW, KST, LY, MSa and IM designed the study. KG, KS, DG, MSt, IM, MSa led the intervention development with input from ET, MJR, AR, JC, SL, SML, FC, ELR, SW, HJ, EW, HCW, KST, LY, MSa and IM. DG, ET, SW, HJ, EW were responsible for recruitment and data collection. KG and DG led on the data analysis, with support from MSa and IM. KG drafted the manuscript, with initial support from DG, MSa and IM. All authors critically reviewed the manuscript, contributing important intellectual content and approved the final manuscript.

Data sharing statement

The data that support the findings of this study are available from the corresponding author upon reasonable request.

References

1 Johansson SG., Bieber T, Dahl R, *et al.* Revised nomenclature for allergy for global use: Report of the Nomenclature Review Committee of the World Allergy

For peer review only

Organization, October 2003. *J Allergy Clin Immunol* 2004;**113**:832–6. doi:10.1016/j.jaci.2003.12.591

2 Bylund S, Von Kobyletzki LB, Svalstedt M, *et al.* Prevalence and incidence of atopic dermatitis: A systematic review. *Acta Derm Venereol* 2020;**100**:320–9. doi:10.2340/00015555-3510

3 Margolis JS, Abuabara K, Bilker W, *et al.* Persistence of mild to moderate atopic dermatitis. *JAMA Dermatology* 2014;**150**:593–600. doi:10.1001/jamadermatol.2013.10271

4 National Institute for Health and Care Excellence (NICE). Atopic eczema in under 12s: diagnosis and management (Clinical guideline 57). 2007.https://www.nice.org.uk/guidance/cg57 (accessed 27 Aug 2021).

5 Ghio D, Muller I, Greenwell K, *et al.* 'It's like the bad guy in a movie who just doesn't die': A qualitative exploration of young people's adaptation to eczema and implications for self-care. *Br J Dermatol* 2020;**182**:112–8. doi:10.1111/bjd.18046

6 Ghio D, Greenwell K, Muller I, *et al.* Psychosocial needs of adolescents and young adults with eczema: A secondary analysis of qualitative data to inform a behaviour change intervention. *Br J Health Psychol* 2020;**26**:214–23. doi:10.1111/bjhp.12467

7 Kosse RC, Bouvy ML, Daanen M, *et al.* Adolescents' perspectives on atopic dermatitis treatment-experiences, preferences, and beliefs. *JAMA Dermatology* 2018;**154**:824–7. doi:10.1001/jamadermatol.2018.1096

8 Teasdale E, Muller I, Sivyer K, *et al.* Views and experiences of managing eczema: systematic review and thematic synthesis of qualitative studies. *Br J Dermatol* 2021;**184**:627–37. doi:10.1111/bjd.19299

For peer review only

9 De Vere Hunt IJ, McNiven A, Roberts A, *et al.* 'Not just a piece of skin in front of you' - A qualitative exploration of the experiences of adolescents with eczema and psoriasis with healthcare professionals. *BMJ Open* 2021;**11**. doi:10.1136/bmjopen-2020-041108

10 Ridd MJ, King AJL, Le Roux E, *et al.* Systematic review of self-management interventions for people with eczema. *Br J Dermatol* 2017;**177**:719–34. doi:10.1111/bjd.15601

11 Pickett K, Frampton G, Loveman E. Education to improve quality of life of people with chronic inflammatory skin conditions: a systematic review of the evidence. *Br J Dermatol* 2016;**174**:1228–41. doi:10.1111/bjd.14435

12 Staab D, Diepgen TL, Fartasch M, *et al.* Age related, structured educational programmes for the management of atopic dermatitis in children and adolescents: Multicentre, randomised controlled trial. *Br Med J* 2006;**332**:933–6. doi:10.1136/bmj.332.7547.933

13 Moore EJ, Williams A, Manias E, *et al.* Eczema workshops reduce severity of childhood atopic eczema. *Australas J Dermatol* 2009;**50**:100–6. doi:10.1111/j.1440-0960.2009.00515.x

14 Muller I, Stuart B, Sach T, *et al.* Supporting self-care for eczema: Protocol for two randomised controlled trials of ECO (Eczema Care Online) interventions for young people and parents/carers. *BMJ Open* 2021;**11**. doi:10.1136/bmjopen-2020-045583

15 Sivyer K, Teasdale E, Greenwell K, *et al.* Supporting parents and carers to manage and co-manage their child's eczema: Intervention planning and optimisation for Eczema Care Online for Families.

For peer review only

For peer review only - http://bmjopen.bmj.com/site/about/guidelines.xhtml

16 Bradbury K, Morton K, Band R, *et al.* Using the person-based approach to optimise a digital intervention for the management of hypertension. *PLoS One* 2018;**13**:1–18. doi:10.1371/journal.pone.0196868

17 Band R, Bradbury K, Morton K, *et al.* Intervention planning for a digital intervention for self-management of hypertension: a theory-, evidence- and person-based approach. *Implement Sci* 2017;**12**:25. doi:10.1186/s13012-017-0553-4

18 Greenwell K, Sivyer K, Vedhara K, *et al.* Intervention planning for the REDUCE maintenance intervention: a digital intervention to reduce reulceration risk among patients with a history of diabetic foot ulcers. *BMJ Open* 2018;**8**:e019865. doi:10.1136/bmjopen-2017-019865

19 O'Cathain A, Croot L, Duncan E, *et al.* Guidance on how to develop complex interventions to improve health and healthcare. *BMJ Open* 2019;**9**:e029954. doi:10.1136/bmjopen-2019-029954

20 Yardley L, Morrison L, Bradbury K, *et al.* The person-based approach to intervention development: Application to digital health-related behavior change interventions. *J Med Internet Res* 2015;**17**:e30. doi:10.2196/jmir.4055

21 Moore G, Audrey S, Barker M, *et al.* Process evaluation of complex interventions: UK medical research council (MRC) guidance. MRC 2014. https://www.mrc.ac.uk/documents/pdf/mrc-phsrn-process-evaluation-guidance-final/ (accessed 27 Aug 2021).

22 Yardley L, Ainsworth B, Arden-Close E, *et al.* The person-based approach to enhancing the acceptability and feasibility of interventions. *Pilot Feasibility Stud* 2015;**1**:37. doi:10.1186/s40814-015-0033-z

For peer review only - http://bmjopen.bmj.com/site/about/guidelines.xhtml

23 Santer M, Muller I, Yardley L, *et al.* Supporting self-care for families of children with eczema with a web-based intervention plus health care professional support: Pilot randomized controlled trial. *J Med Internet Res* 2014;**16**:1–27. doi:10.2196/jmir.3035

24 van Zuuren EJ, Fedorowicz Z, Christensen R, *et al.* Emollients and moisturisers for eczema. *Cochrane Database Syst Rev* Published Online First: 6 February 2017. doi:10.1002/14651858.CD012119.pub2

25 Greenwell K, Ghio D, Muller I, *et al.* Taking charge of eczema self-management: a qualitative interview study with young people with eczema. *BMJ Open* 2021;**11**:e044005. doi:10.1136/bmjopen-2020-044005

26 Michie S, Richardson M, Johnston M, *et al.* The Behavior Change Technique Taxonomy (v1) of 93 Hierarchically Clustered Techniques: Building an International Consensus for the Reporting of Behavior Change Interventions. *Ann Behav Med* 2013;**46**:81–95. doi:10.1007/s12160-013-9486-6

27 Michie S, Atkins L, West R. *The Behaviour Change Wheel: A Guide to Designing Interventions*. London: : Silverback Publishing 2014.

28 Cane J, O'Connor D, Michie S. Validation of the theoretical domains framework for use in behaviour change and implementation research. *Implement Sci* 2012;**7**:37. doi:10.1186/1748-5908-7-37

29 Mccloskey BM, Loranger H, Nielsen J. *Teenagers (Ages 13-17) on the Web*. Nielsen Norman Group 2013.

30 Nielsen J. *Designing for Young Adults (Ages 18-25)*. Nielsen Norman Group 2016.

31 Williams S, Yardley L, Weal M. Introduction to LifeGuide: opensource software for creating online interventions for health care, health promotion and training. *Knowl*

For peer review only - http://bmjopen.bmj.com/site/about/guidelines.xhtml

Resour 2010;**187**:90.

32 Hoffmann TC, Glasziou PP, Boutron I, *et al.* Better reporting of interventions: template for intervention description and replication (TIDieR) checklist and guide. *BMJ* 2014;**348**:g1687. doi:10.1136/bmj.g1687

33 Greenwell K, Sereda M, Coulson NS, *et al.* 'That's just how I am': a qualitative interview study to identify factors influencing engagement with a digital intervention for tinnitus self-management. *Br J Health Psychol* 2020;:bjhp.12486. doi:10.1111/bjhp.12486

34 Halls A, Nunes D, Muller I, *et al.* 'Hope you find your "eureka" moment soon': A qualitative study of parents/carers' online discussions around allergy, allergy tests and eczema. *BMJ Open* 2018;**8**. doi:10.1136/bmjopen-2018-022861

35 Santer M, Chandler D, Lown M, *et al.* Views of oral antibiotics and advice seeking about acne: a qualitative study of online discussion forums. *Br J Dermatol* 2017;**177**:751–7. doi:10.1111/bjd.15398

36 National Institute for Health and Care Excellence (NICE). Babies, children and young people's experience of healthcare [NG204]. 2021.https://www.nice.org.uk/guidance/indevelopment/gid-ng10119 (accessed 27 Aug 2021).

37 Muller I, Santer M, Morrison L, *et al.* Combining qualitative research with PPI: reflections on using the person-based approach for developing behavioural interventions. *Res Involv Engagem* 2019;**5**:1–8. doi:10.1186/s40900-019-0169-8

For peer review only

Figure legends

Figure 1 Intervention development process for ECO intervention. ***Key:*** *PBA = Person-Based Approach; EBA = Evidence-based approach; TBA = Theory-based approach.*

Figure 2 Logic model for Eczema Care Online (ECO) for young people

For peer review only

For peer review only - http://bmjopen.bmj.com/site/about/guidelines.xhtml

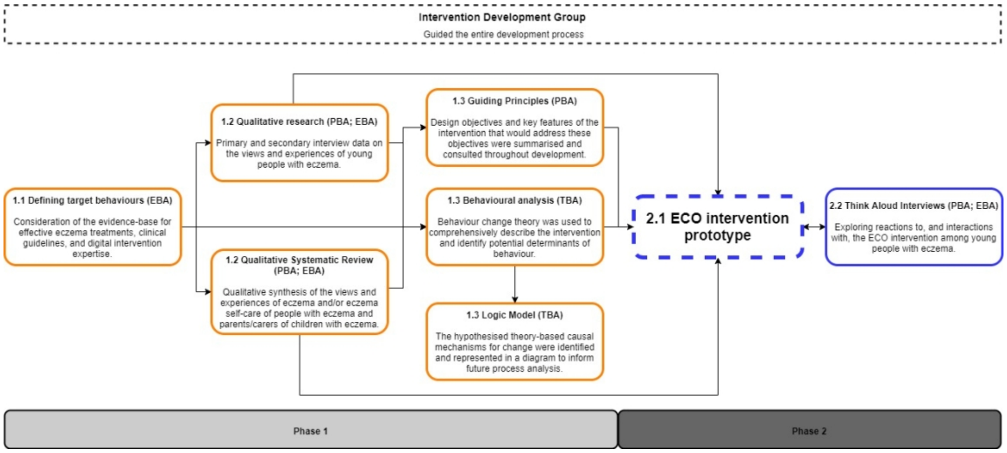

Intervention development process for ECO intervention. Key: PBA = Person-Based Approach; EBA = Evidence-based approach; TBA = Theory-based approach.

55x24mm (600 x 600 DPI)

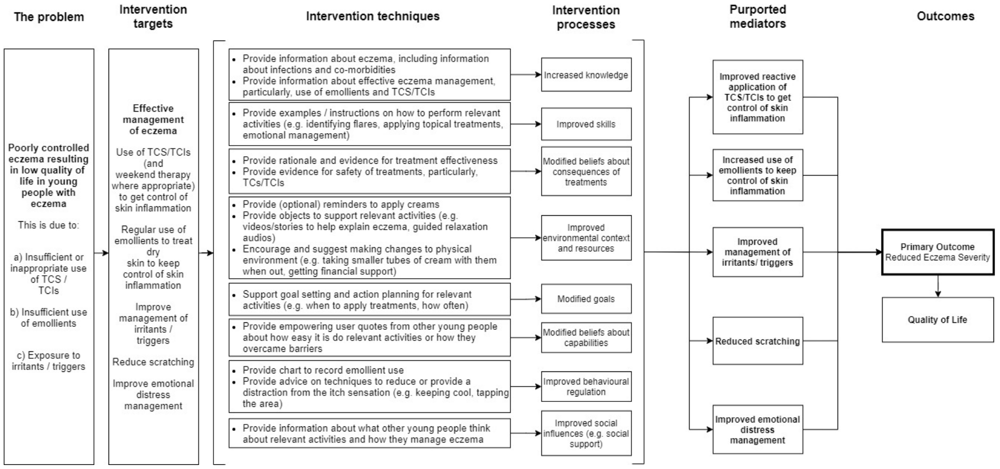

Logic model for Eczema Care Online (ECO) for young people

57x26mm (600 x 600 DPI)

Supplementary Material 1: Behavioural analysis for the Eczema Care Online (ECO) intervention for young people using the Behaviour Change Wheel (BCW), Theoretical Domains Framework (TDF) and Behaviour Change Techniques Taxonomy (BCTv1)

Key: TCS = Topical Corticosteroids; YP = Young People; EO = Barrier emerged from expert opinion; QSR = Barrier/facilitator emerged from systematic review of qualitative literature with adults with eczema and parents/carers of children with eczema; QI = Barrier/facilitator emerged from qualitative interview research with young people.

**Barriers/*facilitator* to target behaviour**	**Intervention components**	**Target construct (BCW)**	**Theoretical domain (TDF)**	**Intervention function (BCW)**	**Behaviour Change Technique (using BCTv1)**
**Target behaviour: Increased emollient use**					
Belief that emollients (in general or a specific brand) do little to control eczema [QSR; QI]<br><br>*Belief that emollients are useful for preventing dry/cracking skin [QI]*	• Provide persuasive and credible information about the effectiveness of emollients, including scientific evidence, user quotes, and videos<br>• Provide rationale for how emollients control eczema	Psychological capability; Reflective motivation; Social opportunity	Beliefs about consequences; Knowledge; Social Influences	Education; Persuasion; Modelling	5.1 Information about health consequences<br>6.2 Social comparison<br>6.3 Information about others' approval<br>9.1 Credible source
	The 2-week challenge:<br>• Provide an emollient chart to allow YP to record how their skin is after applying emollients	Reflective Motivation	Beliefs about consequences	Education; Persuasion	5.1 Information about health consequences<br>2.4 Self-monitoring of outcomes of behaviour
	• Provide advice on how to choose an effective emollient (e.g. list of available emollients, disadvantages of using cosmetic moisturisers)	Psychological capability	Skills	Training	4.1 Instructions on how to perform the behaviour
Concerns about the safety and side effects (e.g. stinging, dependency) of emollients smell, feel, and appearance of emollients [QSR; QI QI]	• Provide persuasive and credible information about the safety of emollients and risk of side effects (including their flammability), including scientific evidence, user quotes and videos	Psychological capability; Reflective motivation; Social opportunity	Beliefs about consequences; Knowledge; Social Influences	Education; Persuasion; Modelling	5.1 Information about health consequences<br>6.2 Social comparison<br>6.3 Information about others' approval<br>9.1 Credible source
	• Provide advice on how to choose the right emollient (e.g. using different emollients at different times of the day, highlighting that different	Psychological capability	Skills	Training	4.1 Instructions on how to perform the behaviour

For peer review only - http://bmjopen.bmj.com/site/about/guidelines.xhtml

**Barriers/*facilitator* to target behaviour**	**Intervention components**	**Target construct (BCW)**	**Theoretical domain (TDF)**	**Intervention function (BCW)**	**Behaviour Change Technique (using BCTv1)**
	emollients have different constituents, smells, and feel)<br>• Provide advice on disguising emollients when around others/away from home (e.g. putting emollient in smaller containers)				
	The 2-week challenge:<br>• Provide an emollient chart to allow YP to record how their skin is each day after applying emollients and how they have found the emollients (e.g. side effects, texture)	Reflective Motivation	Beliefs about consequences	Education; Persuasion	5.1 Information about health consequences<br>2.4 Self-monitoring of outcomes of behaviour
Concerns about the psychosocial impact of emollients (e.g. feeling self-conscious) [QSR; QI]	• Provide strategies to reduce the psychosocial impact of emollients (e.g. feeling less self-conscious)<br>• Provide quotes from YPs explaining how they overcame some of the psychosocial consequences of emollients	Psychological capability; Reflective Motivation; Social opportunity	Beliefs about consequences; Beliefs about capabilities; Knowledge; Skills; Social influences	Education; Persuasion; Training; Modelling	5.3 Information about social and environmental consequences<br>5.6 Information about emotional consequences<br>6.2 Social comparison<br>6.3 Information about others' approval<br>9.1 Credible source
Inconvenience of using emollients when away from home [QSR; QI]	• Provide advice on using emollients when away from home (e.g. requesting smaller tubes from health professional)	Physical Opportunity	Environmental context and resources	Environmental restructuring	12.1 Restructuring the physical environment
Inconvenience of emollients rubbing off (e.g. on clothes and bed sheets) [QSR; QI]	• Provide advice on how to choose the right emollient and avoiding them rubbing off (e.g. put on loose clothing, allowing adequate time for absorption, leaving it to dry in a warm room)	Psychological capability	Skills	Training	4.1 Instructions on how to perform the behaviour

For peer review only - http://bmjopen.bmj.com/site/about/guidelines.xhtml

Barriers/*facilitator* to target behaviour	Intervention components	Target construct (BCW)	Theoretical domain (TDF)	Intervention function (BCW)	Behaviour Change Technique (using BCTv1)
Belief that emollients are time-consuming to apply and to find the right one/competing time pressures [QSR; QI]<br><br>Forgetting to apply emollients [QI]<br><br>*Having an emollient routine [QI]*	• Provide information on how to integrate emollient use into everyday life<br>• Reassure YP that applying emollients should not be time-consuming<br>• Advise YP to plan when they will apply their emollients (i.e. ensure they allocate time)	Psychological capability; Reflective Motivation	Knowledge; Skills; Goals	Education; Training; Enablement	1.4 Action planning<br>4.1 Instructions on how to perform the behaviour<br>5.3 Information about social and environmental consequences
	The 2-week challenge:<br>• Suggest YP to apply their emollient daily<br>• Suggest YP plan when they will apply their emollients<br>• Suggest YP apply their creams at the same time in the same context each day<br>• Allow YP to choose how many times per day they aim to apply their emollients and suggest they choose a particular time of the day or situation (e.g. after showering) to apply.<br>• Provide an emollient chart to record whether they have used their emollients at their agreed times.	Reflective motivation; Psychological capability	Goals; Skills; Behavioural regulation	Enablement; Training	1.1 Goal setting (behaviour)<br>1.4 Action planning<br>2.3 Self-monitoring of behaviour<br>8.1 Behavioural practice/rehearsal<br>8.3 Habit formation
	• Allow users to set up regular reminders to apply emollients by email or text messages and decide on the frequency of these reminders<br>• Provide an emollient chart that acts as a reminder by suggesting YP record whether they have used their	Physical opportunity	Environmental context and resources	Environmental structuring	7.1 Prompts/cues

For peer review only - http://bmjopen.bmj.com/site/about/guidelines.xhtml

**Barriers/*facilitator* to target behaviour**	**Intervention components**	**Target construct (BCW)**	**Theoretical domain (TDF)**	**Intervention function (BCW)**	**Behaviour Change Technique (using BCTv1)**
	emollients at their agreed times [2-week challenge]				
Belief that finding an emollient that works best for you is inconvenient, confusing and time-consuming [QSR; QI]	• Acknowledge how frustrating and time-consuming this process can be<br>• Provide quotes from YP emphasising the importance of finding the right emollient	Psychological capability; Reflective motivation; Social opportunity	Beliefs about consequences; Beliefs about capabilities; Knowledge; Social Influences	Education; Persuasion; Modelling	5.1 Information about health consequences<br>6.2 Social comparison<br>6.3 Information about others' approval<br>9.1 Credible source
Belief that you don't need to apply the emollient every day or only when you need it/eczema is bad [QI]	• Provide information on how often to apply emollients and the rationale for doing this	Psychological capability; Reflective Motivation	Knowledge; Skills	Education; Training	4.1 Instructions on how to perform the behaviour<br>5.1 Information about health consequences
Lack of skills regarding how to apply emollients/ low self-efficacy [QSR]	• Provide instructions on how to correctly applying emollients, including how much emollient to apply	Psychological capability	Skills	Training	4.1 Instructions on how to perform the behaviour
Ran out of emollients [EO]<br><br>*Stocking up on emollient [QI]*	• Provide advice on how to obtain more emollients/avoid running out	Physical Opportunity	Environmental context and resources	Environmental structuring	12.5 Adding objects to the environment
Cost of emollients [QI]	• Provide advice about financial benefits YP can apply for or strategies for reducing the cost of emollients	Physical Opportunity; Psychological Capability; Reflective Motivation	Environmental context and resources; Knowledge; Beliefs about consequences	Environmental structuring; Education; Persuasion	5.3 Information about social and environmental consequences<br>12.5 Adding objects to the environment
Difficulties in getting health professionals to	• Provide advice on how to prepare for appointments with health professionals	Psychological Capability	Skills	Training	4.1 Instructions on how to perform the behaviour

For peer review only - http://bmjopen.bmj.com/site/about/guidelines.xhtml

**Barriers/*facilitator* to target behaviour**	**Intervention components**	**Target construct (BCW)**	**Theoretical domain (TDF)**	**Intervention function (BCW)**	**Behaviour Change Technique (using BCTv1)**
prescribe different emollients [QI]<br><br>*Preparing and researching for consultations [QI]*					
**Target behaviour: Improved use of topical corticosteroids (TCS) or Topical Calcineurin Inhibitors (TCI)**					
Belief that TCSs/TCIs are not effective enough for managing flare-ups (e.g. provide only temporary relief before their eczema returned) [QI]	• Provide persuasive and credible information about the effectiveness of TCS/TCIs, including scientific evidence, user quotes and videos<br>• Provide rationale for how TCS/TCIs control eczema<br>• Provide advice on whether emollients are also needed	Psychological capability; Reflective motivation; Social opportunity	Beliefs about consequences; Knowledge; Social Influences	Education; Persuasion; Modelling	5.1 Information about health consequences<br>6.2 Social comparison<br>6.3 Information about others' approval<br>9.1 Credible source
	• Provide advice on how to choose an effective TCS/TCIs	Psychological capability	Skills	Training	4.1 Instructions on how to perform the behaviour
Concerns about the long-term safety of TCSs [QSR; QI; EO]	• Provide persuasive and credible information about the safety of TCSs/TCIs (e.g. skin thinning, wrinkling, dependency), including scientific evidence, user quotes and videos	Psychological capability; Reflective motivation; Social opportunity	Beliefs about consequences; Knowledge; Social Influences	Education; Persuasion; Modelling	5.1 Information about health consequences<br>6.2 Social comparison<br>6.3 Information about others' approval<br>9.1 Credible source
Concerns about the immediate side effects (e.g. stinging) of TCSs/TCIs [QI]	• Provide reassuring advice about the temporary nature of side effects (e.g. stinging) and how to choose the right TCS/TCIs (i.e. no side effects)	Psychological capability; Reflective motivation;	Skills; Beliefs about consequences; Knowledge	Education; Persuasion; Training	4.1 Instructions on how to perform the behaviour<br>5.1 Information about health consequences
Uncertainty regarding when to start and finish TCSs/TCIs [QI]	• Provide information on when to apply TCSs/TCIs and for how long	Psychological capability	Skills	Training	4.1 Instructions on how to perform the behaviour

For peer review only

For peer review only - http://bmjopen.bmj.com/site/about/guidelines.xhtml

**Barriers/*facilitator* to target behaviour**	**Intervention components**	**Target construct (BCW)**	**Theoretical domain (TDF)**	**Intervention function (BCW)**	**Behaviour Change Technique (using BCTv1)**
Lack of skills regarding how to apply TCSs/TCIs / low self-efficacy [EO]	• Provide instructions on how to correctly apply TCSs/TCIs, including how much to apply	Psychological capability	Skills	Training	4.1 Instructions on how to perform the behaviour
Belief that using more TCS than prescribed will conceal their eczema or make their eczema flare-up pass quicker [QI]	• Provide information on how much TCS/TCIs to apply	Psychological capability	Skills	Training	4.1 Instructions on how to perform the behaviour
Uncertainty regarding the difference between steroids [QI]	• Provide information on what type of steroids are available and which ones they should use on different body parts and why	Psychological capability	Skills	Training	4.1 Instructions on how to perform the behaviour
Ran out of TCS/TCIs /not being able to get hold of their preferred TCS (e.g. out of stock) [QI]<br><br>*Stocking up on TCS/TCIs [QI]*	• Provide information on how to obtain more TCS/TCIs and avoid running out	Physical Opportunity	Environmental context and resources	Environmental structuring	12.5 Adding objects to the environment
Belief that health professionals (including pharmacists) are reluctant or hesitant to prescribe TCSs/the right potency [QI]	• Provide advice on how to talk to health professionals<br>• Provide advice on how to choose an effective TC/TCIs S (e.g. list of available TCSs/TCIs)	Psychological capability	Skills	Training	4.1 Instructions on how to perform the behaviour

For peer review only - http://bmjopen.bmj.com/site/about/guidelines.xhtml

**Barriers/*facilitator* to target behaviour**	**Intervention components**	**Target construct (BCW)**	**Theoretical domain (TDF)**	**Intervention function (BCW)**	**Behaviour Change Technique (using BCTv1)**
	• Provide explanation of how the health professional decides on the TCS/TCI prescription				
Cost of TCSs/TCIs [QI]	• Provide advice about financial benefits YP can apply for or strategies for reducing the cost of TCSs/TCIs	Psychological capability; Reflective motivation	Beliefs about consequences; Knowledge	Education; Persuasion	5.3 Information about social and environmental consequences
**Target behaviour: Management of irritants and triggers**					
Lack of knowledge regarding common irritants and triggers [EO]	• Provide information on common irritants and triggers<br>• Provide information on misconceptions (e.g. food allergies)	Psychological capability; Reflective Motivation	Beliefs about consequences; Knowledge; Skills	Education; Persuasion; Training	4.1 Instructions on how to perform the behaviour<br>5.1 Information about health consequences
Belief that you can't avoid some triggers (e.g. stress)/Belief that the benefits of avoidance (e.g. avoiding a brief flare-up) do not outweigh the costs (e.g. not being able to go swimming) [QI]	• Where appropriate, provide advice on how to minimise the effects of irritants/triggers (e.g. apply emollients before and/or afterwards)<br>• Where appropriate, provide advice on how to avoid or reduce contact with certain irritants/triggers (e.g. soaps, high temperatures, sweat), including when away from home	Psychological capability; Reflective Motivation	Beliefs about consequences; Knowledge; Skills	Education; Persuasion; Training	4.1 Instructions on how to perform the behaviour<br>5.1 Information about health consequences

For peer review only - http://bmjopen.bmj.com/site/about/guidelines.xhtml

**Barriers/*facilitator* to target behaviour**	**Intervention components**	**Target construct (BCW)**	**Theoretical domain (TDF)**	**Intervention function (BCW)**	**Behaviour Change Technique (using BCTv1)**
**Target behaviour: Reduce scratching**					
Belief that scratching is not that bad [EO]	• Explain itch-scratch cycle and that scratching makes itch/eczema worse	Psychological capability; Reflective Motivation	Beliefs about consequences; Knowledge	Education; Persuasion	5.1 Information about health consequences<br>5.6 Information about emotional consequences
Feeling of itchiness and desire for instant relief [QI]<br><br>*Wearing appropriate clothes [QI]; Keeping cool [QI]; Emollient use [QI]; Antihistamine [QI]; Tapping [QI]; Keeping nails short/wear gloves [QI]*	• Provide information on the factors that make scratching more likely (e.g. hot temperature, lack of sleep)<br>• Provide advice on reducing itchiness (e.g. keeping cool, emollient use, tapping the area, clench fist, wearing clothes with long arms and legs, put emollient in fridge to keep cool).<br>• Provide advice on sleep management.<br>• Provide techniques to prevent the negative consequences of scratching (e.g. keep nails short, wear gloves) and how to deal with the annoyance of others telling them to stop scratching (e.g. ask people to suggest things they can do, like tap skin, instead).	Physical Capability; Psychological Capability	Environmental context and resources; Skills; Behavioural Regulation	Training; Education; Enablement; Environmental restructuring	4.1 Information on how to perform a behaviour<br>4.2 Information about antecedents<br>12.3 Avoidance/reducing exposure to cues for behaviour<br>12.5 Adding objects to the environment<br>12.6 Body changes
Scratching is a habit [QI]<br><br>*Distraction techniques [QI]*	• Provide techniques to raise awareness of scratching (e.g. record instances of scratching)<br>• Provide techniques to stop scratching (e.g. distraction, relaxation techniques, replace scratching with an alternative behaviour such as clenching fists).	Psychological Capability; Automatic Motivation; Physical Opportunity	Environmental context and resources; Skills; Behavioural Regulation	Training; Enablement; Environmental restructuring	2.3 Self-monitoring of behaviour<br>4.1 Information on how to perform a behaviour<br>8.2 Behaviour substitution<br>8.4 Habit reversal<br>12.4 Distraction<br>12.5 Adding objects to the environment
**Target behaviour: Engaging in emotional management techniques**					

For peer review only - http://bmjopen.bmj.com/site/about/guidelines.xhtml

**Barriers/*facilitator* to target behaviour**	**Intervention components**	**Target construct (BCW)**	**Theoretical domain (TDF)**	**Intervention function (BCW)**	**Behaviour Change Technique (using BCTv1)**
Belief that emotional management techniques will do little to control their eczema or help with difficult emotions [EO]/ Belief that stress (e.g. during exam time) can't be avoided [QI]<br><br>*Belief that emotions affect eczema [QI]*	• Explain the necessity of emotional management techniques for promoting engagement with the other behaviours and provide evidence that they are effective for dealing with difficult emotions	Psychological capability; Reflective Motivation	Beliefs about consequences; Knowledge	Education; Persuasion	5.1 Information about health consequences<br>5.6 Information about emotional consequences
	• Provide user quotes demonstrating how emotional management techniques helped other users to take control of their eczema	Psychological capability; Reflective motivation; Social opportunity	Beliefs about consequences; Knowledge; Social Influences	Education; Persuasion; Modelling	5.1 Information about health consequences<br>6.2 Social comparison<br>6.3 Information about others' approval<br>9.1 Credible source
Lack of understanding regarding how to do the emotional management techniques [EO]	• Provide guidance on how to do the emotional management techniques	Psychological capability	Skills	Training	4.1 Instructions on how to perform the behaviour
	• Provide guided audio recordings of emotional management exercises	Physical opportunity	Environmental context and resources	Environmental structuring	12.5 Adding objects to the environment
Lack of confidence in ability to practice emotional management techniques [EO]	• Provide YP quotes demonstrating how easy it was for other users to practice the emotional management techniques	Psychological capability; Reflective motivation; Social opportunity	Beliefs about consequences; Beliefs about capabilities; Knowledge; Social Influences	Education; Persuasion; Modelling	6.2 Social comparison<br>6.3 Information about others' approval<br>9.1 Credible source

For peer review only

For peer review only - http://bmjopen.bmj.com/site/about/guidelines.xhtml

Supplementary Material 2: Screenshot of page in introductory module providing key behavioural messages on topical treatments

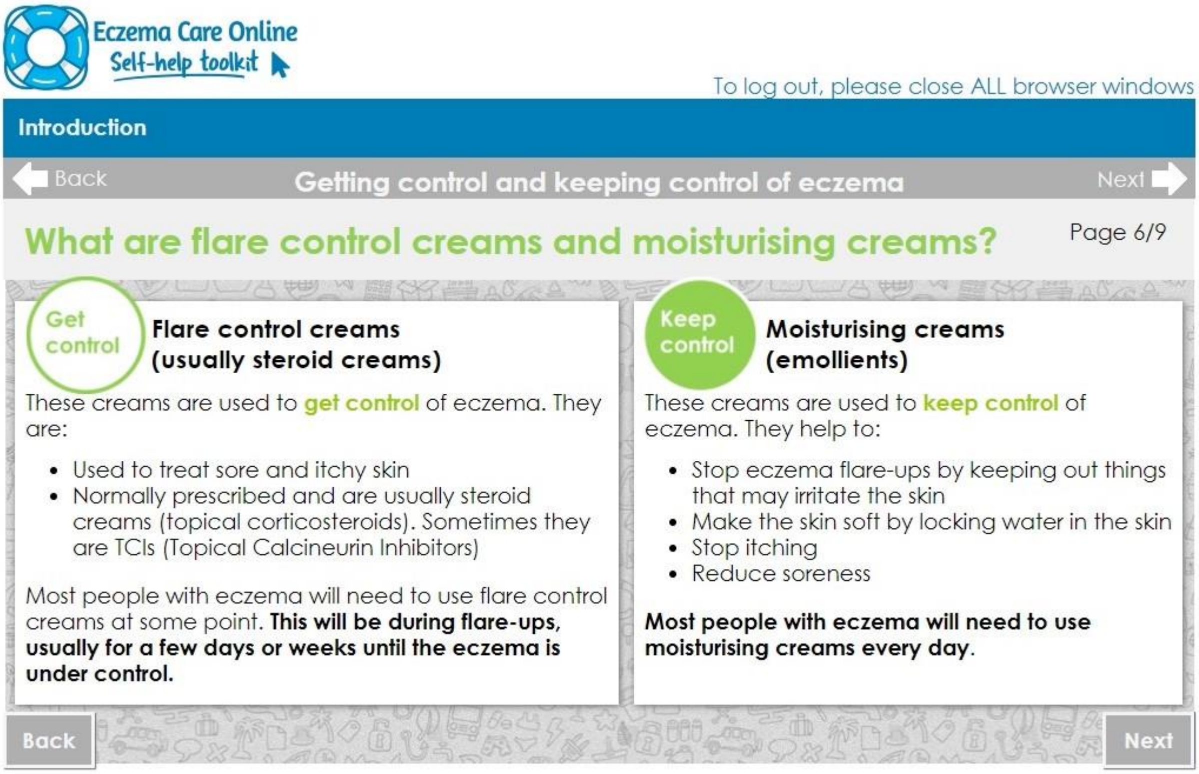

view only

For peer review only - http://bmjopen.bmj.com/site/about/guidelines.xhtml

Supplementary Material 3: Screenshot of video explaining how eczema affects the skin barrier to provide a better understanding of eczema and a rationale for how topical treatments work

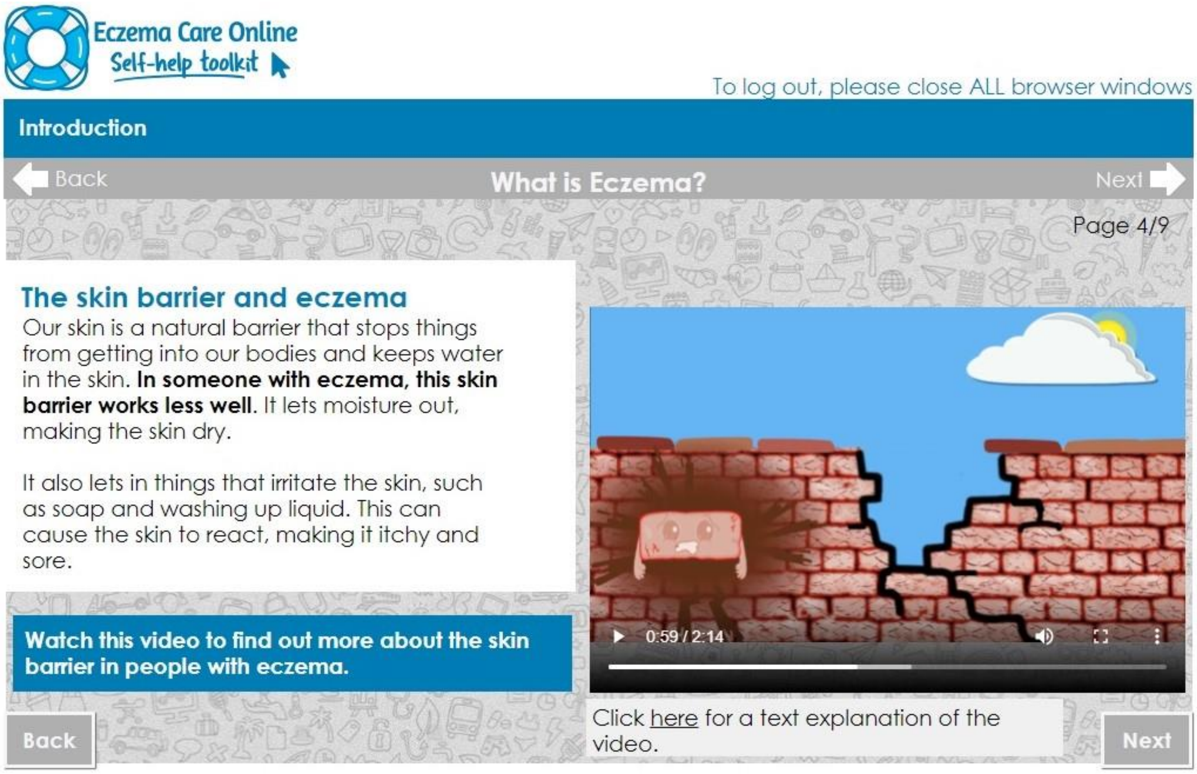

For peer review only - http://bmjopen.bmj.com/site/about/guidelines.xhtml

Supplementary Material 4: Screenshot of simple eczema assessment and subsequent feedback on which of the core treatment modules (emollients or topical corticosteroids) would be most relevant depending on whether they were currently experiencing an eczema flare-up

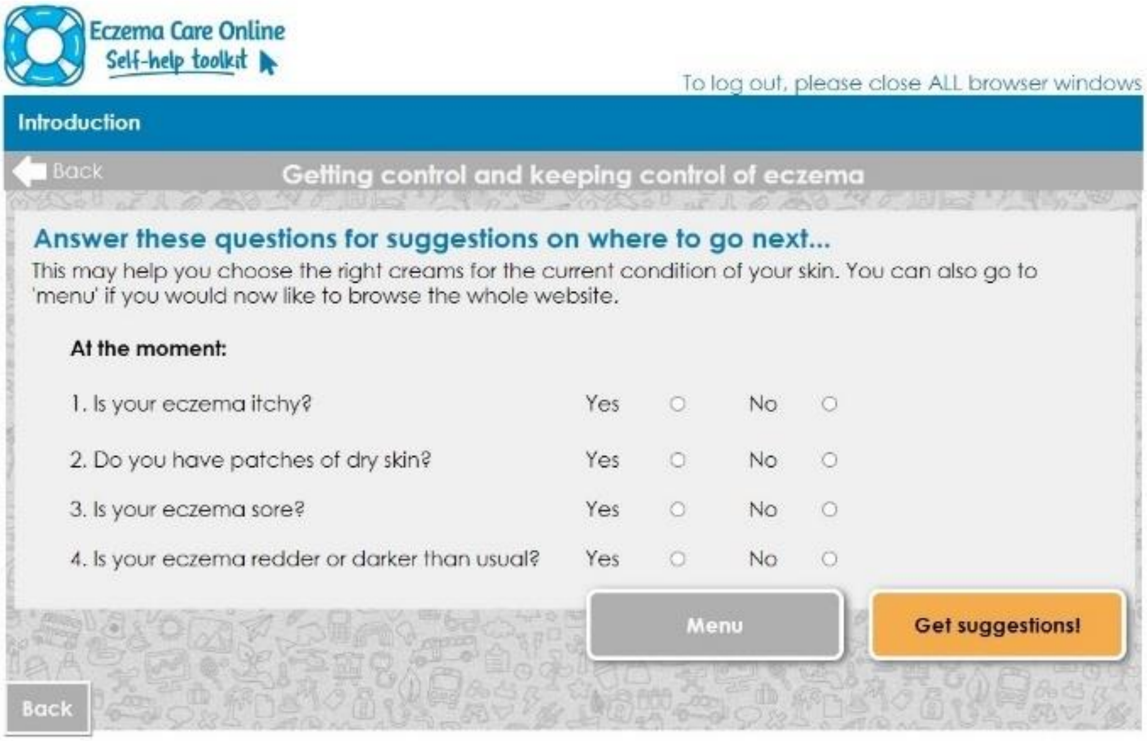

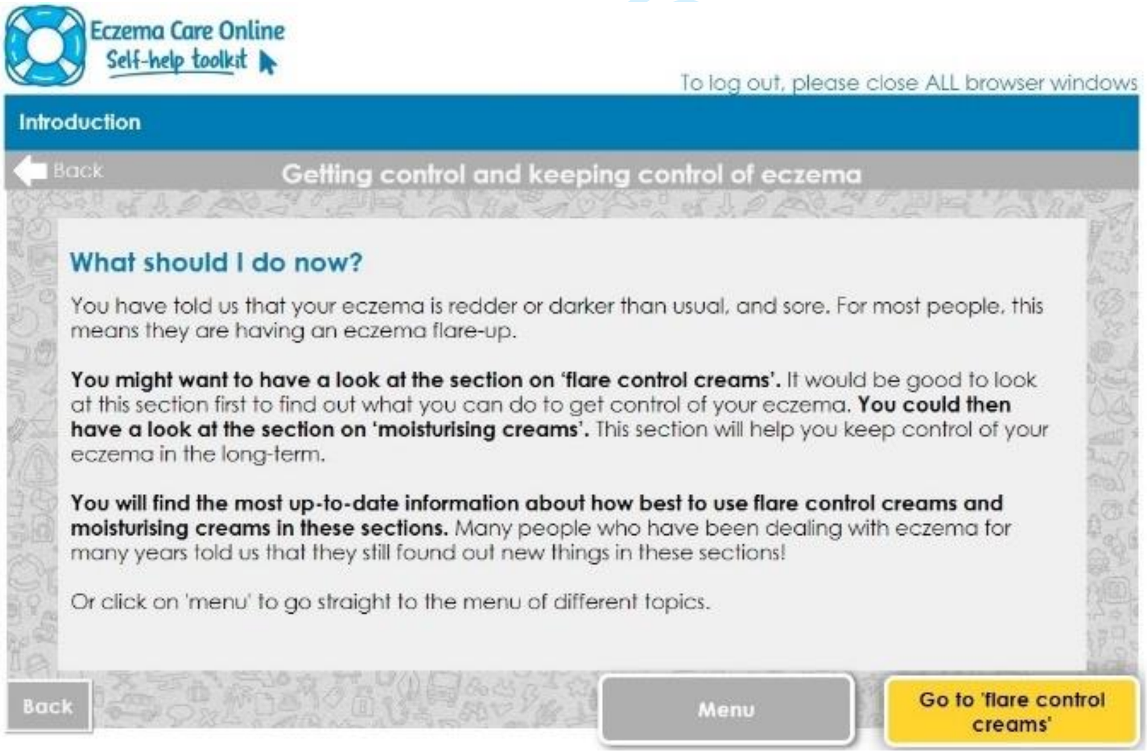

Supplementary Material 5: Screenshot of page explaining the menu of different information topics and quotes from other young people with eczema

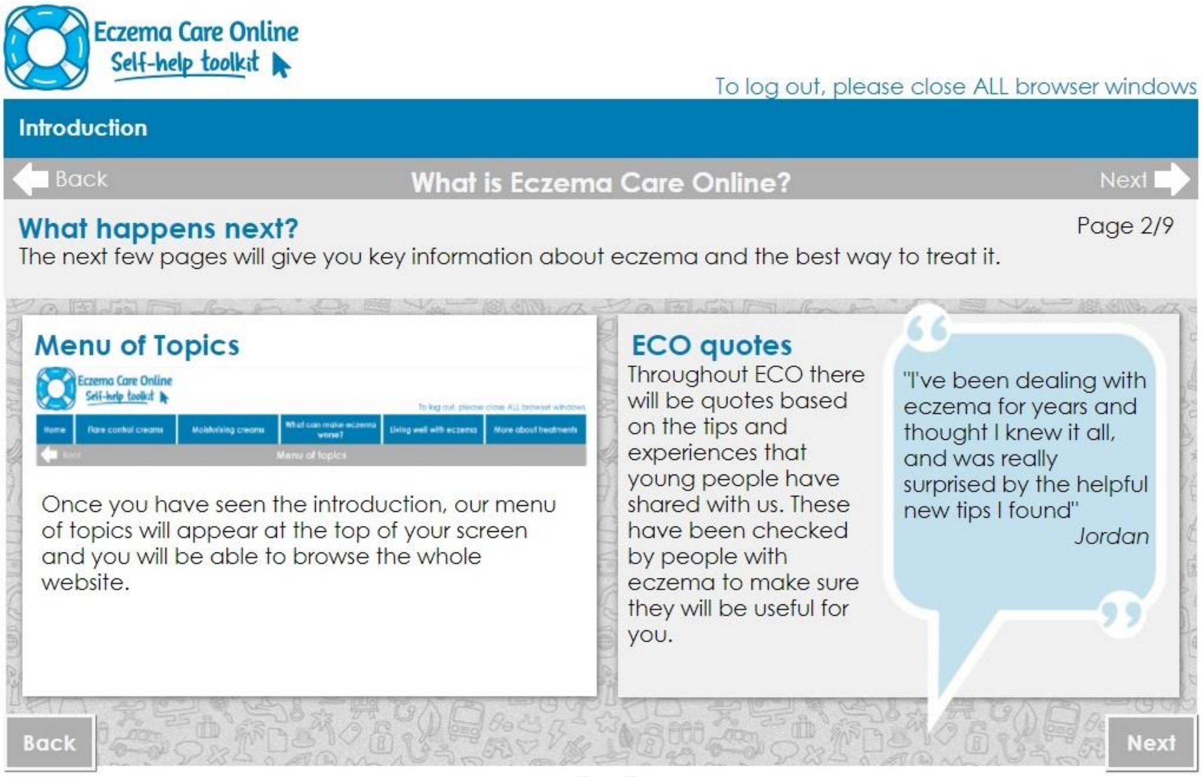

For peer review only - http://bmjopen.bmj.com/site/about/guidelines.xhtml

Supplementary Material 6: Description of Eczema Care Online (ECO) for Young People in accordance with the Template for Intervention Description and Replication (TIDieR) checklist

- 1. BRIEF NAME: Eczema Care Online (ECO) for Young People
- 2. WHY: Online interventions could potentially be used to support young people with eczema; however, research developing such interventions has been limited and systematic reviews show the need for systematically developed evidence-based interventions to support self-management of eczema.
- 3 & 4. WHAT (materials and procedures): ECO for Young People is an online behavioural intervention for young people with eczema aged 13-25 years with mild to severe eczema. It aims to reduce eczema severity, targeting 5 key behaviours: (1) Increase their use of emollients to maintain skin hydration and prevent flare-ups; (2) Improve their use of TCS or TCIs through reactive applications of these treatments in response to flare-ups or, where appropriate, regular intermittent ("weekend") preventative applications of TCS/TCI if emollients are insufficient as maintenance therapy; (3) Improve their management of irritants and triggers; (4) Improve their emotional management; and (5) Reduce scratching. See the table below for an overview of the modules included in ECO for Young People. For more detail about specific intervention techniques, see the behavioural analysis in Supplementary Material 1.

Table 1: Overview of the key intervention components of ECO for Young People

Menu	Modules	Description
Home Page	N/A	• Signposting to key modules that young people haven't accessed yet<br>• Quick links to interactive content e.g. videos
Flare control creams	• Flare control cream video<br>• What are they?<br>• Are they safe?<br>• Common questions<br>• When and how do I use them?<br>• How can I find the right cream?<br>• Golden rules	• Evidence-based education and messages to increase motivation and competence to use topical corticosteroids (TCS), including; evidence of safety and support for skills development e.g. videos/photos showing use of TCS
Moisturising creams	• Moisturising creams video<br>• What are they and how to they help?<br>• Are they safe?	• Evidence-based education and messages to increase motivation and competence to use emollients, including; evidence of safety and

For peer review only - http://bmjopen.bmj.com/site/about/guidelines.xhtml

	• Common Concerns<br>• When and how do I use them?<br>• How do I find the right cream?<br>• Take the two-week challenge<br>• Golden rules	support for skills development e.g. videos/photos showing how to use emollients
What can make eczema worse	• Living with eczema video<br>• Bathing, showering, and washing clothes<br>• Cosmetics, make-up and shaving<br>• Diet and allergies<br>• Weather and holidays<br>• Swimming and physical activity	• Evidence-based education and messages to increase motivation and competence in identifying key eczema triggers and managing them, including practical tips
Living well with eczema	• Beat the itch<br>• Sleep<br>• Stress and eczema<br>• Studying and work<br>• Eczema and money	• Evidence-based education and messages to increase motivation and competence to manage some of the symptoms and psychosocial impacts of eczema e.g. itching and scratching, disturbed sleep, stress, including techniques to manage them
More about treatments	• Talking to health professionals<br>• Infections<br>• Topical Calcineurin Inhibitors<br>• Other treatments<br>• Other resources	• Evidence-based education and messages to increase motivation and competence to manage other aspects of treatments e.g. consulting with health professionals and other treatments

- 5. WHO PROVIDED: ECO for Young People is provided direct to patients via their GP surgeries.
- 6. HOW: ECO for Young People is entirely online, although users are encouraged to contact their GP, pharmacist or nurse, for example, to obtain or change their treatments, or to treat potential infections.
- 7. WHERE: ECO for Young People is intended for any young person (aged 13-25) with mild to severe eczema. It can be implemented in primary or secondary care. The intervention is online and designed to be accessed using computers, tablets and phones via a web-browser.
- 8. WHEN and HOW MUCH: For the randomised controlled trial study, young people are given access to the intervention if their GP identifies that they have eczema that has required treatment in the last 12 months. Once registered young people can use the online intervention as much or as little as they like.

For peer review only - http://bmjopen.bmj.com/site/about/guidelines.xhtml

- 9. TAILORING: Young people have access to all the modules on ECO, however the website uses signposting based on four questions about the current state of their eczema. They will then be recommended either the module on emollients or flare control creams module depending on whether it appears they are currently experiencing an eczema flare-up. Key modules will also be recommended, one or two at a time, on the intervention home page if they haven't been viewed yet. These modules/menus are: 1) moisturising creams; 2) flare control creams, 3) bathing, showering and washing clothes; 4) beat the itch, 5) stress and eczema.
- 10. MODIFICATIONS: An additional email was circulated in response to COVID-19 that provided further advice about handwashing.
- 11. HOW WELL: The intervention is currently being tested in a randomised control trial with young people with eczema (ISRCTN79282252). Information about the results of the randomised controlled trial, including both quantitative and qualitative data, will be presented in future publications.

For peer review only

COREQ checklist

No	Item	Guide questions/description	Comments	Location in manuscript
**Domain 1: Research team and reflexivity**				
Personal Characteristics				
1.	Interviewer/facilitator	Which author/s conducted the interview or focus group?	DG, HJ, EW	Methods Section 2.2
2.	Credentials	What were the researcher's credentials? *E.g. PhD, MD*	Postdoctoral experienced qualitative researcher (DG) and two medical students (HJ, EW) supervised by DG and another postdoctoral researcher (ET)	Methods Section 2.2
3.	Occupation	What was their occupation at the time of the study?	See above	Methods Section 2.2
4.	Gender	Was the researcher male or female?	All female	Methods Section 2.2
5.	Experience and training	What experience or training did the researcher have?	See No 2	Methods Section 2.2
Relationship with participants				
6.	Relationship established	Was a relationship established prior to study commencement?	Some of the participants were known to the two medical student interviewers (HJ, EW).	-
7.	Participant knowledge of the interviewer	What did the participants know about the researcher? e.*g. personal goals, reasons for doing the research*	Participants were told that the interviews were to help improve the ECO intervention.	-
8.	Interviewer characteristics	What characteristics were reported about the interviewer/facilitator? e.g. *Bias, assumptions, reasons and interests in the research topic*	One of the interviewers was involved in intervention development (DG), which could be a potential source of bias.	-
**Domain 2: study design**				
Theoretical framework				
9.	Methodological orientation and Theory	What methodological orientation was stated to underpin the study? *e.g. grounded theory, discourse analysis, ethnography,*	Person-based approach to intervention development	Intervention development methodology section

For peer review only

No	Item	Guide questions/description	Comments	Location in manuscript
		phenomenology, content analysis		
Participant selection				
10.	Sampling	How were participants selected? *e.g. purposive, convenience, consecutive, snowball*	Purposive, opportunistic, and snowballing sampling	Methods Section 2.2
11.	Method of approach	How were participants approached? e.*g. face-to-face, telephone, mail, email*	Invitation letter from their GP practice or advertising, opportunistic and snowball sampling of students at the University of Southampton.	Methods Section 2.2
12.	Sample size	How many participants were in the study?	28	Methods Section 2.2
13.	Non-participation	How many people refused to participate or dropped out? Reasons?	Data not collected	-
Setting				
14.	Setting of data collection	Where was the data collected? e.*g. home, clinic, workplace*	At participants' homes or at a location of the participants' choice (e.g. at the university).	Methods Section 2.2
15.	Presence of non-participants	Was anyone else present besides the participants and researchers?	A family member was present for 9 of the interviews.	Methods Section 2.2
16.	Description of sample	What are the important characteristics of the sample? *e.g. demographic data, date*	Demographic information can be found in Table 2. Interviews were carried out during October 2018 to April 2019.	Methods Section 2.2
Data collection				
17.	Interview guide	Were questions, prompts, guides provided by the authors? Was it pilot tested?	Interview question guide not applicable for think aloud research	-
18.	Repeat interviews	Were repeat interviews carried out? If yes, how many?	Yes, 2 people took part in two interviews	Methods Section 2.2
19.	Audio/visual recording	Did the research use audio or visual recording to collect the data?	Audio recording.	Methods Section 2.2
20.	Field notes	Were field notes made during and/or after the	Field notes were made after all interviews.	Methods Section 2.2

For peer review only

No	Item	Guide questions/description	Comments	Location in manuscript
		interview or focus group?		
21.	Duration	What was the duration of the interviews or focus group?	45-90 minutes	Methods Section 2.2
22.	Data saturation	Was data saturation discussed?	We considered data saturation to be reached once no further important changes were required	Methods Section 2.2
23.	Transcripts returned	Were transcripts returned to participants for comment and/or correction?	No	-
**Domain 3: analysis and findings**				
Data analysis				
24.	Number of data coders	How many data coders coded the data?	Two (KG & DG), with input from 5 other researchers	Methods Section 2.2
25.	Description of the coding tree	Did authors provide a description of the coding tree?	No	-
26.	Derivation of themes	Were themes identified in advance or derived from the data?	Not applicable.	-
27.	Software	What software, if applicable, was used to manage the data?	Microsoft excel was used for Table of changes	-
28.	Participant checking	Did participants provide feedback on the findings?	No	-
Reporting				
29.	Participant checking	Were participant quotations presented to illustrate the themes / findings? Was each quotation identified? e*.g. participant number*	Participant quotations are presented and each quotation is identified by a pseudonym and their gender and age is noted.	Results Section 2.2
30.	Data and findings consistent	Was there consistency between the data presented and the findings?	Yes	Results Section 2.2
31.	Clarity of major themes	Were major themes clearly presented in the findings?	Not applicable.	-
32.	Clarity of minor themes	Is there a description of diverse cases or	Not applicable.	-

For peer review only

No	Item	Guide questions/description	Comments	Location in manuscript
		discussion of minor themes?		

For peer review only
